# Dopamine encodes deep network teaching signals for individual learning trajectories

**DOI:** 10.1016/j.cell.2025.05.025

**Published:** 2025-06-11

**Authors:** Samuel Liebana, Aeron Laffere, Chiara Toschi, Louisa Schilling, Jessica Moretti, Jacek Podlaski, Matthias Fritsche, Peter Zatka-Haas, Yulong Li, Rafal Bogacz, Andrew Saxe, Armin Lak

**Affiliations:** 1 Department of Physiology, Anatomy & Genetics, University of Oxford, Oxford OX1 3PT, UK; 2 School of Life Sciences, Peking University, Beijing 100871, China; 3 MRC Brain Network Dynamics Unit, University of Oxford, Oxford OX1 3TH, UK; 4 Gatsby Computational Neuroscience Unit & Sainsbury Wellcome Centre, University College London, London W1T 4JG, UK

## Abstract

Striatal dopamine plays fundamental roles in fine-tuning learned decisions. However, when learning from naive to expert, individuals often exhibit diverse learning trajectories, defying understanding of its underlying dopaminergic mechanisms. Here, we longitudinally measure and manipulate dorsal striatal dopamine signals in mice learning a decision task from naive to expert. Mice learning trajectories transitioned through sequences of strategies, showing substantial individual diversity. Remarkably, the transitions were systematic; each mouse’s early strategy determined its strategy weeks later. Dopamine signals reflected strategies each animal transitioned through, encoding a subset of stimulus-choice associations. Optogenetic manipulations selectively updated these associations, leading to learning effects distinct from that of reward. A deep neural network using heterogeneous teaching signals, each updating a subset of network association weights, captured our results. Analyzing the model’s fixed points explained learning diversity and systematicity. Altogether, this work provides insights into the biological and mathematical principles underlying individual long-term learning trajectories.

## Graphical abstract

**Graphical abstract:**
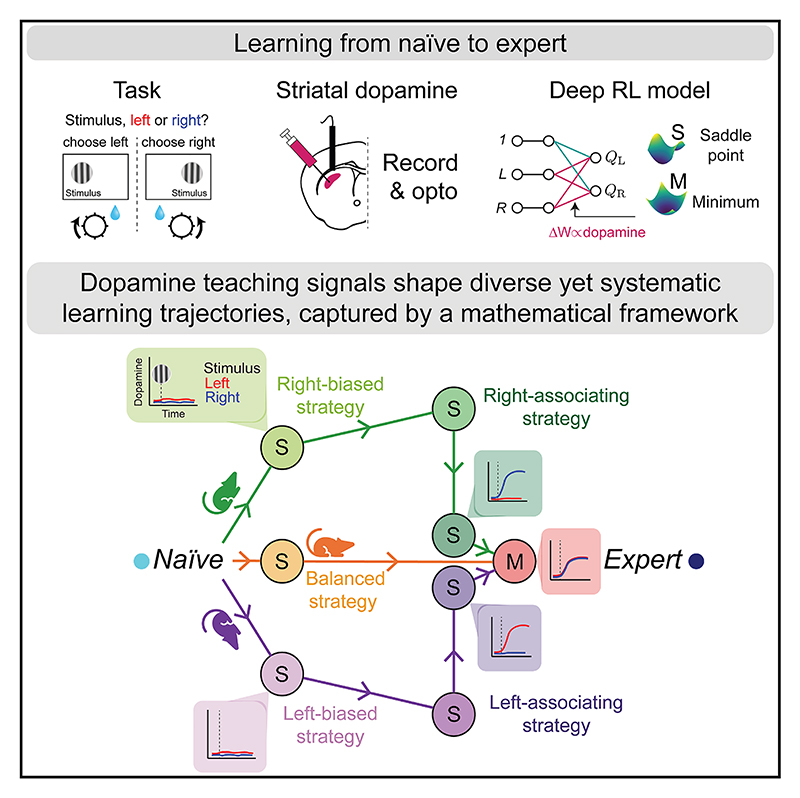

## Introduction

Many abilities are learned over long time periods spanning weeks or months. For instance, a novice can take many months to learn how to play tennis. Such long-term learning entails substantial individual variability. Individuals tend to follow diverse trajectories from naive to expert, discovering different strategies at different times, just as tennis players often develop distinct playing styles. Decades of experimental and theoretical work have provided fundamental insights into the neuronal circuits and computations underlying learning.^1–12^ However, these studies have mostly examined learning over short timescales. Thus, the behavioral correlates, neuronal underpinning, and computational principles governing long-term learning trajectories and their individual diversity are not understood.

Past neuroscientific studies have often operationalized learning as the fine tuning of an already learned task in expert animals. Expert animals tend to exploit their knowledge of the task, leading to only small changes in behavior and negligible individual diversity. The results from these studies have been broadly captured by standard reinforcement learning (RL) models that (re-)learn the value of predetermined states through reward prediction error (RPE) updates.^3,13,14^ Long-term learning, however, involves discovering state representations that enable the adoption of increasingly effective strategies. This often leads to substantial diversity in strategy transitions across individuals through learning. It has been difficult to determine the degree of systematicity in what appears to be an inherently stochastic long-term learning process—does behavior early in learning determine future behavior? Moreover, the principles governing individual diversity are not understood. Thus, it remains an open question whether the RL framework can account for long-term learning trajectories within and across individuals.

Neuronal signals across several brain areas are important for learning.^15,16^ Many studies have shown that dopamine (DA) neurons and brain areas receiving DA signals, such as the striatum, are essential for learning to make decisions.^17–30^ These DA signals encode RPE and have been shown to drive plasticity in cortico-striatal synapses.^31–36^ In particular, for learning to make perceptual decisions (i.e., decisions guided by incoming sensory information) dorsal regions of striatum, and their cortical and dopaminergic inputs, play critical roles.^4^ However, the role of dorsal striatal DA signals in long-term learning to make perceptual decisions remains unknown. Crucially, if DA signals underlie long-term learning trajectories, these teaching signals should reflect and shape the diverse intermediate strategies employed by different animals throughout learning. Testing this hypothesis requires probing DA signals during learning of a decision task that admits different strategies, ensuring sufficient yet tractable individual diversity. However, past studies have often recorded DA signals in expert animals, instead of recording throughout learning from naive to expert.

Here, we address these questions by examining learning trajectories and dorsal striatal DA signals in tens of mice learning a visual decision-making task from naive to expert. Mice learned transitioning through sequences of strategies that varied widely across individuals. However, the strategy transitions were remarkably systematic; each animal’s future behavior could be predicted weeks in advance. Dorsal striatal DA signals reflected the diverse yet systematic transitions of learning trajectories. These signals encoded RPEs suitable to shape the intermediate strategies from naive to expert. Optogenetic experiments provided causal evidence for the specific learning effects of these DA signals, which were distinct from the effect of reward on learning. We demonstrate that mice learning trajectories, the DA signals and their optogenetic effects cannot be explained by standard “shallow” RL models with predetermined state representations. Instead, our results suggest that mice refine their state representations throughout learning, a process well captured by a *deep* neural network that employs gradient-based RL. Further, we demonstrate that networks require heterogeneous teaching signals to capture the DA signals and their optogenetic effects. Our deep RL model reproduces the mice’s diverse yet systematic stage-like transitions between behavioral strategies and shows that they emerge from a hierarchy of saddle points. We conclude that the fixed point structure of a deep neural network model provides a general framework for understanding long-term learning within and across individuals.

## Results

### Mice form diverse yet systematic learning trajectories from naive to expert

**Figure 1:**
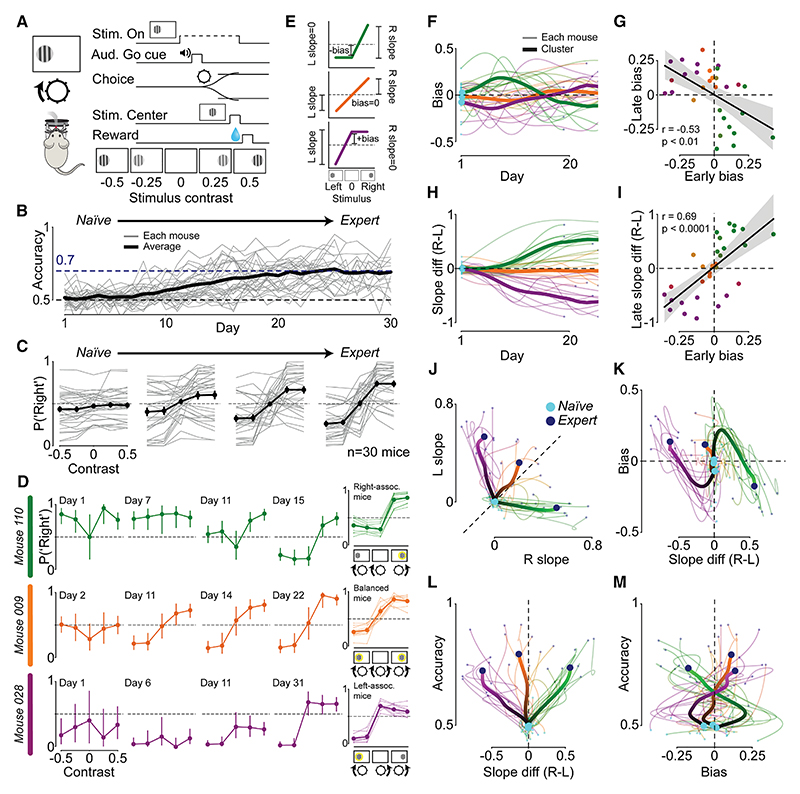
Mice form diverse yet systematic learning trajectories from naive to expert (A) The visual decision task for head-fixed mice (STAR Methods). (B) Accuracy over days per mouse (gray) and averaged across all mice (black, *n =* 30). (C) Psychometric curve over quartiles. Quartiles are defined per mouse by dividing days into 4 groups, with any remainder added to the last group. Negative (positive) contrast values indicate stimuli presented on the left (right) side of the screen. P(“Right”) indicates the probability of reporting “right” side stimulus position. Unless specified differently, error bars indicate ± SEM across mice. (D) First four columns, psychometric curves from 3 example mice on 4 example days throughout learning. Error bars indicate the 95% confidence interval of a two-sided binomial test on P(“Right”). See Figure S1G for chronometric curves. Last column, per mouse (thin) and average expert psychometric curves clustered by trajectory type (thick): right-associating (green), balanced (orange), and left-associating (purple). See Figure S1H for corresponding chronometric curves. Cluster labels for each mouse were obtained from (J), and colors from Figure S1L. (E) Schematic explaining behavioral metrics. Left (right) slope is defined as the absolute difference between P(“Right”) for left (right) stimulus and zero-contrast trials. Bias is defined as the difference between zero-contrast P(“Right”) and balanced choice probability (i.e., 0.5). (F) Bias over days per mouse (thin) and for the 3 clusters from (J) (thick). In right- and left-associating mice, biases increase before reversing (*p* < 0.05; two-sided paired t test first 2 days vs. days 5–6). (G) Regression of early bias (days 4–8) against late bias (final 5 days). Each point represents a mouse. *p* value calculated from the exact distribution of r. Shaded regions indicate 95% confidence interval across mice. (H) Difference between right and left psychometric slopes over days per mouse (thin) and for 3 clusters from (J) (thick). (I) Regression of early bias against late slope difference. (J) Right vs. left slope across days per mouse (thin) and for 3 clusters (thick). The hue of the cluster lines indicate progress through learning. Left-associating (purple), balanced (orange), and right-associating (green) clusters are obtained from dynamic time warping clustering (STAR Methods). (K–M) In order, difference in right and left (R-L) slope vs. bias; R-L slope vs. accuracy and bias vs. accuracy across days per mouse (thin) and for the 3 clusters from (J) (thick). See also Figures S1 and S2.

To study long-term learning of perceptual decision making, we further developed an established visual decision task for head-fixed mice.^37^ In each trial, we presented a visual stimulus (a grating) on the left or right side of a screen. The mouse, head-fixed in front of the screen, reported the stimulus position (left or right) by steering a wheel with its forepaws to bring the stimulus to the center of the screen (Figures 1A and S1A; STAR Methods). The mouse received a drop of water reward for each correct choice (Figure 1A). The contrast of the grating stimulus changed across trials, making the trials easier or harder. Some trials did not have a stimulus (i.e., zero-contrast trials), here, animals were rewarded randomly (50/50) regardless of the wheel movement direction (Figure 1A). We trained the mice over multiple days with a single session each day. Importantly, we kept the task unchanged throughout the entire experiment, presenting the full set of stimuli, task contingencies, and trial timing from day 1 until expert performance (STAR Methods). This ensured that any changes in behavior are a consequence of the animals’ internal learning mechanisms, rather than experimentally imposed changes to the task. We trained 40 mice, 30 of which learned the task reaching accuracies of at least 70% (Figures 1B, S1B, and S1C; median days and trials to reach 70% were 19 days and 3,376 trials, respectively), showing progressively steeper psychometric curves (Figure 1C) and faster choice response times (RTs, Figure S1D).

In early days of learning, mice favored left or right choices to varying degrees, exhibiting flat, but often biased, psychometric curves (Figures 1D, first column, 1F, and 1G). The biases usually increased in initial days (Figures 1F and S1F), and RTs averaged across all trial types decreased (Figures S1D–S1F). This decrease in RTs led to an increase in reward rate, i.e., reward per unit time (Figures S1E and S1F). RTs then started to depend on visual stimuli, i.e., mice showed faster RTs for trials with visual stimuli compared to zero-contrast trials, resulting in chronometric curves that were no longer flat (Figures S1D–S1F). These changes in biases, RTs, and reward rate occurred while choice accuracies were still at chance level, i.e., flat psychometric curves (Figures S1E and S1F). Thus, early days were marked by strategies where the mice ignored the position of the stimulus for making choices, showing decreasing RTs and increasing biases with different directions across animals.

During later days of learning, mice’s choices began to depend on the location of visual stimuli, resulting in psychometric curves with increasing slopes. Importantly, slopes often developed asymmetrically for left and right stimuli, with a vast diversity across mice (Figures 1H–1L). To visualize this diversity, we colored each mouse’s learning trajectory based on the asymmetry of its psychometric slopes (Figure S1L; STAR Methods). While the diversity of slopes formed a continuum across mice, to better visualize the main trends, we clustered slope trajectories over learning (Figure 1J; STAR Methods). In some mice, the slopes increased similarly on both sides, resulting in more balanced psychometric curves (Figure 1J). This indicates that their strategy involved associating both left and right stimuli with their corresponding rewarded choice directions (Figure 1D, middle row). However, in other mice, the slope primarily increased on one side while the other side remained flat, forming a one-sided psychometric curve (Figures 1J and 1D, top and bottom rows). These mice therefore solved the task by forming a single stimulus-choice association: they associated stimuli on the left (right) side of the screen with their corresponding choice direction and made the alternative choice in trials in which the associated stimulus was absent (left-associating and right-associating mice; Figure 1D, rightmost column). Consistent with this strategy, choices in zero-contrast trials and trials with non-associated stimuli were indistinguishable, i.e., the psychometric curve was flat on one side (Figure S1J). These slope asymmetries often persisted as learning progressed (Figures 1L, S1K, and S1N), and their corresponding signatures were observed in RTs (Figures S1H and S1M). Importantly, despite asymmetries in slopes, one-sided and balanced mice reached similar levels of accuracy (Figure 1L). This is because both the presence and absence of stimuli can inform correct choices, as there is only one stimulus per trial. Mice that did not learn despite performing enough trials (i.e., 4,300, well above the median of learners), showed early choice biases but failed to develop psychometric slopes (Figures S1O and S1P). Overall, we observed that in later days mice transitioned to more stimulus-dependent strategies, while exhibiting diversity in their use of stimuli to make choices.

Behavioral transitions throughout learning were systematic. The bias in early days strongly predicted the biases and psychometric curves in later stages of learning (Figures 1G and 1I). Mice with early left bias developed a larger slope on the left side of their psychometric curve, whereas mice with early right bias developed a larger slope on the right side (Figure S1N). To achieve high accuracy with asymmetric slopes, mice with more one-sided strategies reversed their early bias during learning (Figures 1G, 1K, and 1M). A model comparison analysis showed that early biases are likely due to a combination of unequal reward history for left and right choices caused by randomness in the task (e.g., rewards on zero-contrast trials or random sequence of stimuli), together with an initial “innate” bias for one of the choices (e.g., handedness; Figure S1Q). However, these early biases did not appear to depend on imbalances in initial sensory processing. Biases were evident even prior to animals’ use of stimuli (Figure S1F). Further, although pupil and lick rate measures changed in response to stimuli and rewards (Figure S2A), these measures were similar for left and right stimuli in initial days (Figure S2B). Thus, naive mice developed varying levels of bias shaped by their reward history, and this early bias determined which stimuli were associated with choices later in learning.

Taken together, the results show that in learning to make visual decisions from naive to expert, mice exhibited diverse learning trajectories involving systematic transitions through behavioral strategies.

### Dorsal striatal dopamine reflects individual long-term learning trajectories

**Figure 2:**
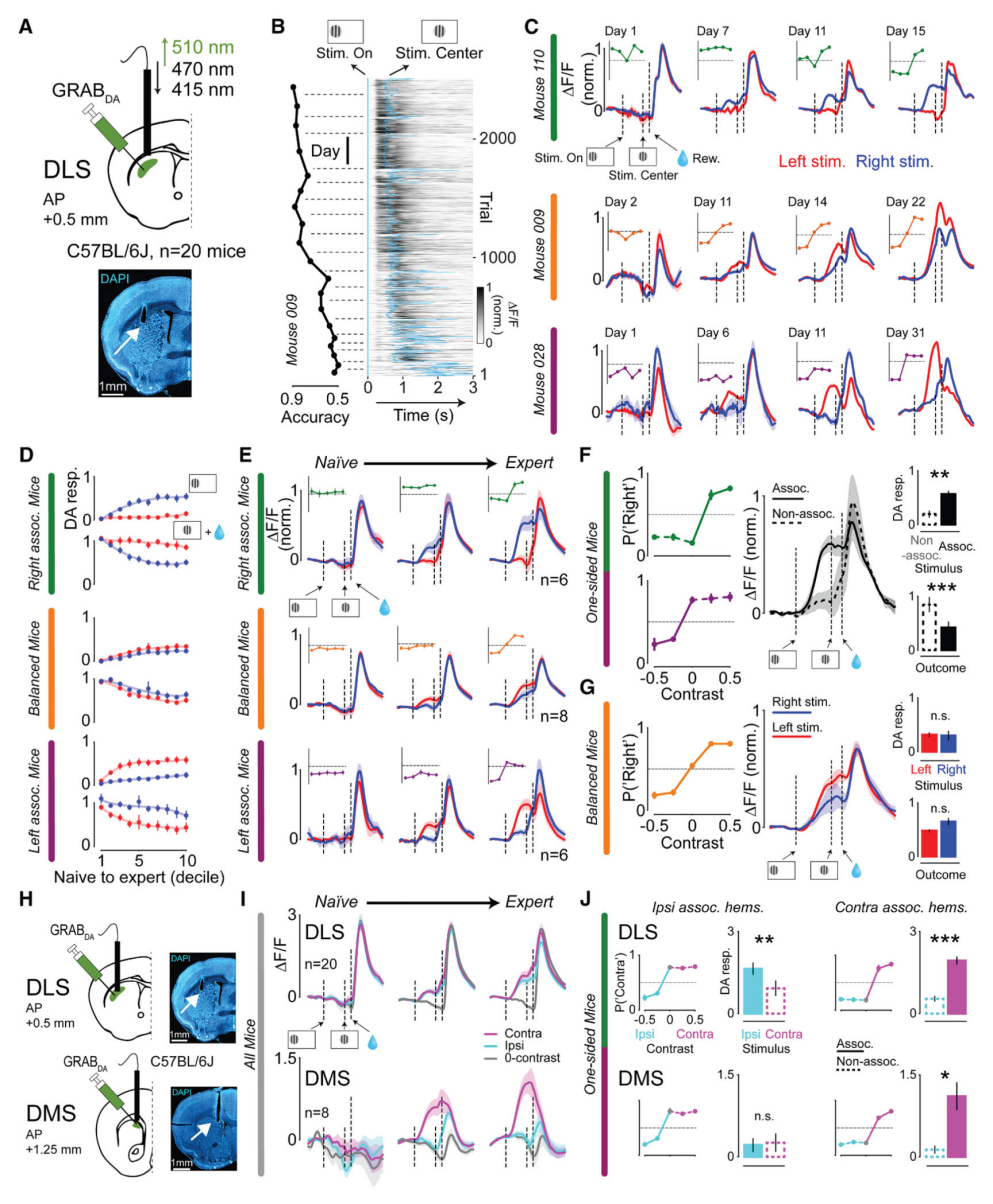
Dorsal striatal DA signals develop over learning, encoding stimulus-choice associations (A) Fiber photometry setup for recording DLS DA release (*n* = 20; STAR Methods). (B) Accuracy over days and simultaneous trial-wise stimulus-aligned DLS DA signals from an example mouse (only correct trials). Blue lines indicate the time of stimulus onset (left) and when the stimulus is brought to the center (i.e., choice completion, right). When indicated, recorded DA levels were normalized to correct for non-task-relevant day-by-day variations in fluorescence. (C) Average time warped DLS DA signals in correct trials with stimulus on the left (red) and right (blue) for the same days and mice as in Figure 1D. Vertical dashed lines indicate stim. onset, stim. center, and reward delivery time. Error bars indicate ± SEM across trials. (D) Average stimulus and outcome DLS DA responses over deciles in correct trials for clusters from Figure 1J. Unless specified differently, error bars indicate ± SEM across mice. (E) Average time warped DLS DA signals in correct trials and average psychometric curves for the three clusters from Figure 1J in initial days (days 1–3, left), early days (days > 3 with accuracy n.s. greater than 0.5, middle), and expert days (accuracy n.s. smaller than 0.7, right). (F) Analysis of left- and right-associating mice DLS DA responses in expert “matched accuracy” days (difference in left and right stimulus trial choice accuracy is <0.1). Left, average psychometric curves in matched accuracy days. Middle, average time warped DLS DA signals in correct trials. Right, DA responses to stimuli and outcome in correct trials. *p* values calculated using two-sided paired t test. (G) Same matched accuracy analysis as in (F) but applied to balanced mice. (H) Fiber photometry setup for recording DA release in DLS and DMS. (I) Average time warped DA signals in correct trials with stimulus ipsilateral (cyan), contralateral (fuchsia), or 0-contrast trials (gray) in initial days (left), early days (middle), and expert days (right). (J) Same matched accuracy analysis as in (F) for one-sided mice, but now with recordings separated for DLS, DMS, and laterality of the associated stimulus (ipsilateral/contralateral). Error bars indicate ± SEM across matched accuracy days. *p* values calculated using two-sided paired t test. n.s., *p* > 0.05, *p < 0.05, **p < 0.005, and ***p < 0.0005, respectively. See also Figures S3, S4, S5, S6, and S12.

We recorded DA release from day 1 until expert behavior in the dorsolateral striatum (DLS). We injected GRAB-DA^38^ in the DLS of wild-type mice and imaged DLS DA release every day during learning through implanted optic fibers (Figures 2A, 2B, and S3A; STAR Methods).

DA release occurred in response to specific events within a trial and changed throughout learning. A linear deconvolution analysis revealed that visual stimulus onset, arrival of the stimulus to the center of the screen in correct trials (i.e., “stim. center”), and water reward (or its absence in incorrect trials) were the main events modulating DA release (Figure S3B). Consistently, wheel movements had negligible representation in DA responses (Figures S3C–S3E). We defined a DA “stimulus” and “outcome” response to examine changes over learning (STAR Methods). The stimulus response quantified DA release in a short time window after stimulus onset. The outcome response was defined as the sum of DA responses to completion of choice (the stimulus arriving to the center of the screen in correct choices, or the stimulus leaving the screen in incorrect choices) and water delivery (or its absence). We summed these responses because the final stimulus position determines whether water will be delivered. In initial days, DLS DA release mostly occurred in response to rewarded outcomes but not visual stimuli. As learning progressed, DA responses to visual stimuli grew and DA responses to water rewards diminished (Figures 2B–2E, S3B, S4A, and S4B). In incorrect trials, DA signals transiently decreased when reward was not delivered (Figure S3B).

The development of DA responses during learning reflected mice’s diverse learning trajectories. DA responses in individual mice matched the development of their psychometric curves (Figures 2C and S4A). In mice developing more one-sided psychometric curves, DA stimulus responses emerged most strongly for stimuli presented on the associated side (top and bottom rows in Figures 2C, 2D, S4A, and S4B). However, in mice developing more balanced psychometric curves, DA responses to stimuli presented on left and right sides were similar (middle rows in Figures 2C, 2D, S4A, and S4B). The diverse behavioral trajectories were also reflected in the rewarded outcome DLS DA response; these DA outcome responses were small after associated stimuli and large after non-associated stimuli (Figures 2C, 2D, S4A, and S4B).

In initial days, when both psychometric and chronometric curves were still flat, there were no DA responses to visual stimuli (Figures 2E, first column, S4C, and S4E). Subsequently, as RTs started to show signatures of mice using visual stimuli (i.e., non-flat chronometric curves), DA responses to stimuli emerged while psychometric curves remained flat (Figures 2E, middle column, S4D, and S4E). These DA responses were strongest for stimuli with faster RTs, which appeared on the same side as the choice bias in one-sided mice (Figures 2E, middle column, S4D, and S4E). We asked whether these signals reflected associations animals were forming between stimuli and choices, or whether they reflected each animal’s bias. To address this, we inspected trials without visual stimuli (zero-contrast trials). We did not observe any significant DA responses before outcome delivery in these trials, despite decreasing RTs over learning and significant choice biases in one-sided mice (Figures S4F and S4G). Thus, the DLS DA stimulus responses emerged as soon as animals started to form an association between visual stimuli and choices, the first signature of this appearing in RTs (Figure S4H). DA responses to stimuli were not evident on day 1 (Figure S4I) and thus did not reflect stimulus novelty.^21,39,40^ DA signatures of associations being formed between stimuli and choices were also evident in DA outcome responses (Figures 2D and S4B). Thus, during early days, DA responses emerged, reflecting the first signatures of learning to associate stimuli and choices.

In later days, DLS DA responses strongly reflected the growing psychometric slopes observed across animals (Figures 2C–2E). In right-associating mice DA responses to stimuli were evident in response to right but not left stimuli (Figure 2E, top row, right). This pattern was opposite in left-associating mice (Figure 2E, bottom row, right). In balanced mice, both left and right stimuli elicited strong DA responses (Figure 2E, middle row, right). The relationship between psychometric slopes and DA responses to stimuli was maintained even after controlling for differences in the choice accuracy for left and right stimuli, i.e., selecting days where the accuracies were matched (Figures 2F, 2G, S4J, and S4K). Thus, in left- and right-associating mice, DA responses to associated stimuli were significantly larger than to non-associated stimuli, despite equally high choice accuracy (Figure 2F). These observations held independent of stimulus laterality with respect to the recorded brain hemisphere (Figures S5A and S5B). Moreover, the encoding of psychometric slope was largely invariant to RTs (Figures S5C and S5D) and the accuracy of pending choice (i.e., correct/incorrect; Figure S5E). These observations held in another cohort of animals (*n* = 6) trained with more graded levels of stimulus contrast to high performance (accuracy 80%–95%; Figure S6A). DLS DA responded to the outcome of correct and incorrect trials, reflecting the difference between outcome value and the learned stimulus-choice association, i.e., the reward prediction that animals formed using visual stimuli. Hence, these DA responses decreased as stimulus-choice associations increased (Figures 2D and S5E, middle row), instead of reflecting choice accuracy (Figures 2F and 2G). Taken together, in later days of learning, DLS DA signals developed substantial responses to visual stimuli, and a corresponding decrease in responses to trial outcome, only if those stimuli were associated with a choice. Thus, while DLS DA signals resemble a classic RPE, depending on each animal’s strategy, they did not always reflect choice accuracy.

The DA signals we observed are specific to DLS; we did not observe them in DA release recordings from the dorsomedial striatum (DMS; Figures 2H and S3F). DMS DA release showed four main characteristics. First, DMS DA responses to reward were negligible from the start of learning, unlike DLS DA (Figures 2I, left column, and S6B–S6D). Second, DMS DA signals, unlike DLS DA, depended on the recorded brain hemisphere, showing particularly strong responses to contralateral and near zero responses to ipsilateral stimuli (Figures 2I, right column, and S6D–S6F). Third, these DMS DA contralateral stimulus responses were modulated by the animal’s strategy; they were only present if the contralateral stimulus was associated with choice (Figures 2J and S6E). Lastly, similar to DLS, DMS DA stimulus responses emerged over learning: they were absent in the initial days of the experiment (Figures 2I, left column, and S6G). We confirmed these observations using a linear deconvolution (Figure S3G). The negligible DMS DA responses to rewards in initial days indicate that these signals are not encoding the RPEs that we observed in DLS DA signals. Therefore, in the following sections, we primarily focus on DLS DA signals.

**Figure 3:**
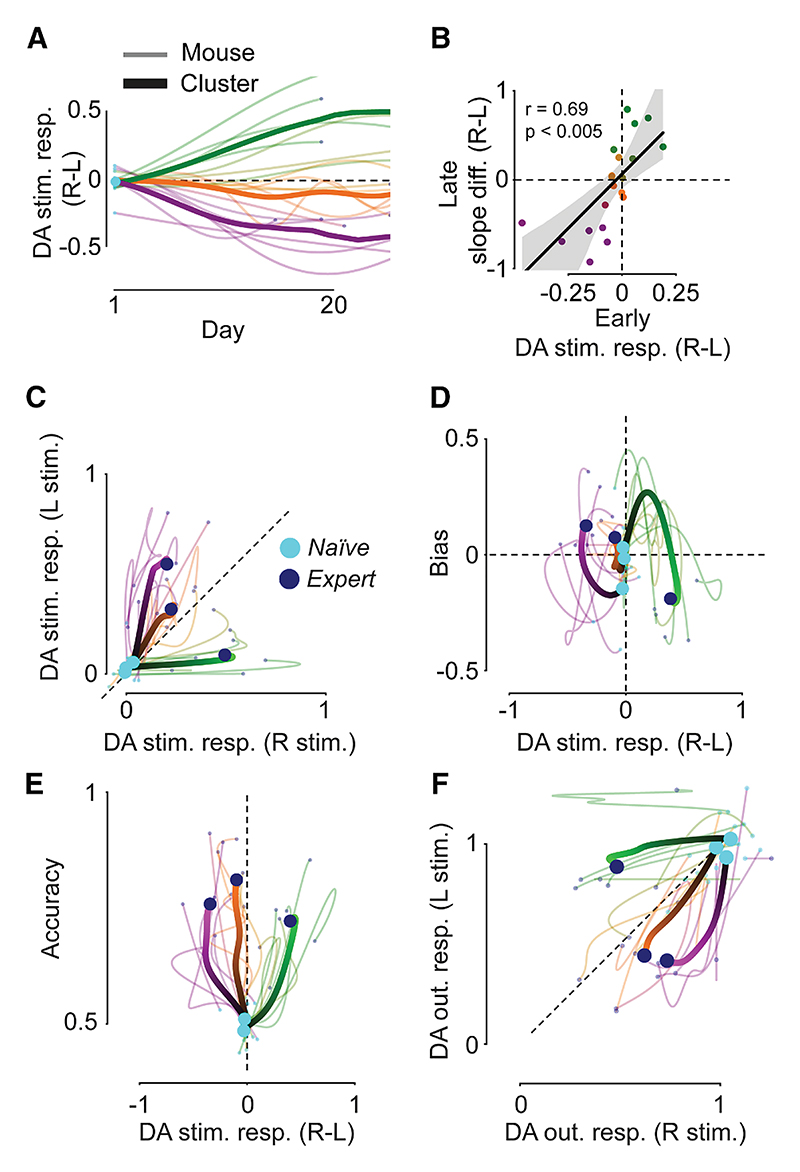
DLS DA signals reflect learning trajectories from naive to expert (A) Difference in DLS DA responses to right and left stimuli (R-L) over days per mouse (thin) and for the clusters from Figure 1J (thick). (B) Regression of early difference in DLS DA responses to R-L stimuli (average across days 4–8) against late R-L slope difference (average across final 5 days of training). Each point represents a mouse. *p* value is calculated from the exact distribution of r. Shaded region indicates 95% confidence interval across mice. (C–F) In order, right vs. left DLS DA stimulus responses; difference in DLS DA responses to right and left stimuli (R-L) vs. bias; difference in DLS DA responses to right and left stimuli (R-L) vs. accuracy; and DLS DA rewarded outcome responses in right-stimulus trials vs. left-stimulus trials across days per mouse (thin) and for the clusters from Figure 1J (thick). The hue of the cluster lines indicates progress through learning. See also Figure S6.

The dynamics of DLS DA signals showed striking similarities to the behavioral learning trajectories (Figure 3). Similar to behavior, DLS DA signals developed systematically. As such, from DA signals throughout the experiment, it was possible to infer the animals’ past and future DA signals (Figures 3A, S6I, and S6J; neural trajectories plotted with colors and clusters obtained from behavior) and behavioral strategies (Figure 3B). DA responses to stimuli developed across days reflecting the associations each animal formed between visual stimuli and choices (Figures 3C–3E). Similarly, DA responses to rewards developed mirroring the evolving stimulus responses (Figures 3F and S6J–S6M). These results demonstrate that DA signals during learning evolve reflecting the intermediate strategies mice employed, suggesting a crucial role for DLS DA in shaping learning trajectories.

### DLS dopamine plays causal roles in driving learning, distinct from classic RPE

**Figure 4:**
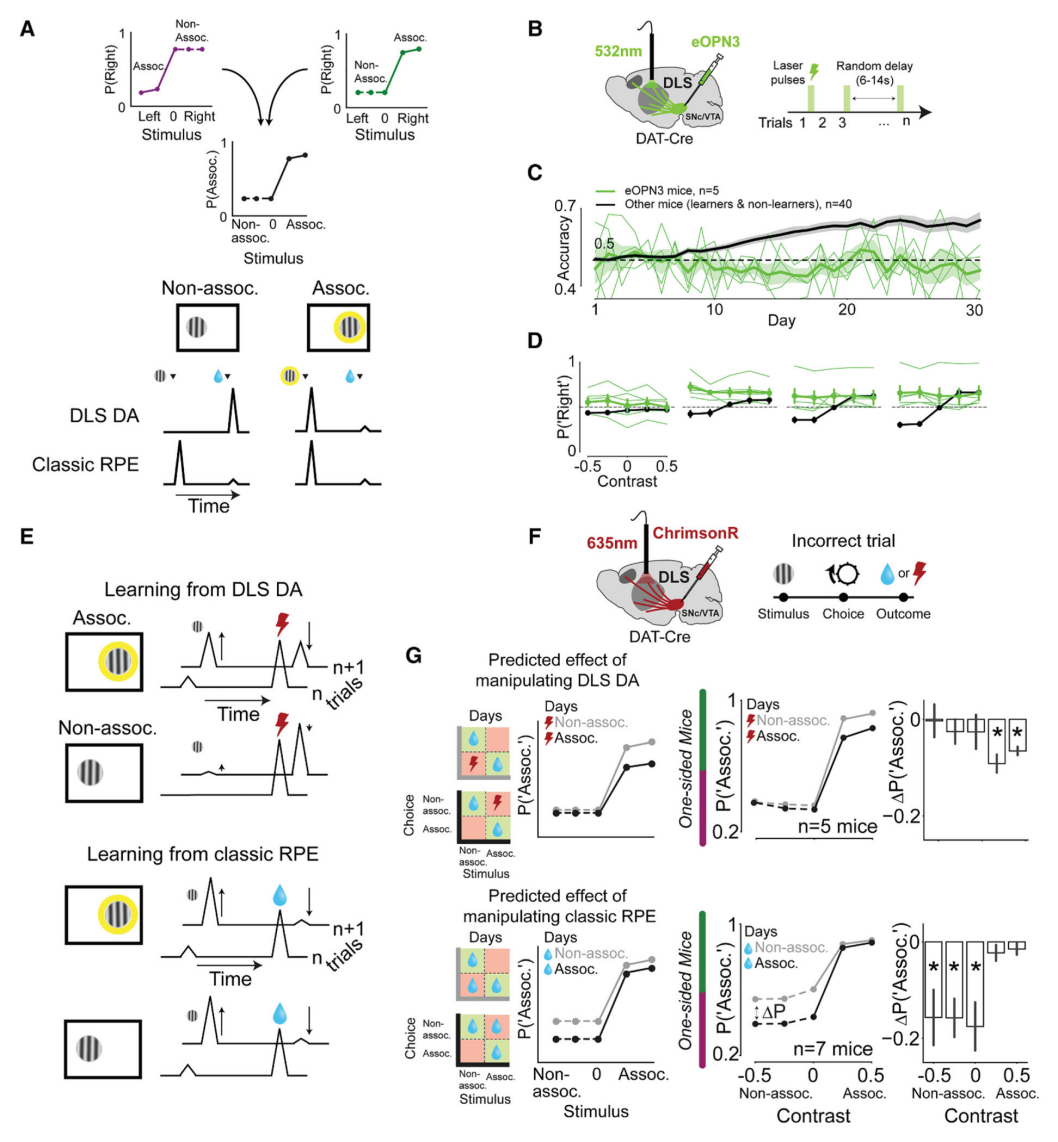
DLS DA is required for learning and updates behavior distinct from a classic RPE (A) Top, schematic explaining the relabeling of the axes from left/right to non-associated/associated. Bottom, schematic showing the observed patterns of DLS DA release in expert one-sided animals, alongside the signals expected from a classic RPE. (B) Schematic for optogenetic inhibition experiment (STAR Methods). Laser pulses were delivered in random intervals of 6–14 s, independent of task trials. (C) Accuracy over days for mice with DLS DA inhibition (green; thin: each mouse, thick: average), and averaged across all the other mice that were trained on the task for at least 4,300 trials (black). In all panels, error bars around averages indicate ± SEM over mice. (D) Psychometric curve over quartiles for DLS DA inhibition mice (green) and all other mice (black). (E) Schematic comparing the predicted learning effect of stimulating DLS DA with that expected from a classic RPE. (F) Schematic for optogenetic excitation experiment (STAR Methods). (G) Left, predicted results of stimulating DLS DA (top) and manipulating classic RPEs (i.e., delivery of water, bottom) at the outcome of error trials, switching in alternating days of experimentation. Right, average psychometric curves, and their difference, on days with DLS DA stimulation (top) and reward delivery (bottom) on error trials with non-associated (gray) and associated (black) stimuli. *p* values calculated using two-sided one-sample t test. See Figure S7E for corresponding chronometric curves. Figure S7F shows the same analysis applied selectively for mice in which both experiments were performed. *p < 0.05. See also Figures S7 and S12.

The DLS DA responses we observed have two main characteristics. First, one-sided animals did not learn to associate one of the visual stimuli with a choice (Figure 4A), despite strong DLS DA reward signals (Figure 2E). This suggests that these animals might not have a neuronal representation of the non-associated stimulus which can be trained using DLS DA signals. Second, in one-sided animals DLS DA did not respond during decisions made based on the absence of visual stimuli (e.g., trials with non-associated stimuli), even after matching the accuracy with those for associated stimuli (Figures 2F and 4A). These characteristics cannot be explained by a classic RPE estimated using all task-relevant cues (e.g., the visual stimulus or its absence). Such RPEs would reflect the expected value (proportional to accuracy) of each task-relevant cue, not only that of visual stimuli (Figure 4A). In contrast, the DLS DA responses reflect predictions and RPEs estimated only using a subset of the task-relevant cues, i.e., the visual stimuli. Based on these characteristics, we predicted distinct learning effects for DLS DA and classic RPEs. DLS DA reward responses should only update associations between choices and associated visual stimuli, whereas classic RPEs update associations between choices and all task-relevant cues. To test this, we performed two optogenetic experiments: inhibiting DLS DA release throughout learning and stimulating DLS DA release in animals after learning.

Longitudinal inhibition of DLS DA substantially impaired learning. To inhibit DLS DA release, we injected the inhibitory opsin eOPN3^41^ into the substantia nigra pars compacta (SNc) of DAT-Cre mice (*n* = 5) and implanted optic fibers above DLS (Figures 4B and S7A). We then illuminated DLS DA axons through the optic fiber with 532-nm laser pulses at random intervals, not locked to any task event (Figure 4B; STAR Methods). These animals showed significantly lower accuracy compared with all other mice that performed a similar number of trials (*n* = 40, learners and non-learners; Figure 4C). The accuracy remained around 50% and psychometric slopes did not develop, indicating that these mice did not learn to use the visual stimuli for decisions (Figures 4C and 4D). Moreover, RTs developed a dependence on visual stimuli much later in training (chronometric curves remained flat; Figures S7B and S7C). However, DLS DA inhibition largely spared other signatures of learning that did not rely on visual stimuli, i.e., developing bias and an overall decrease in RT (Figures S7B and S7C). The inhibition also largely spared gross motor functions; mice decreased RTs over learning and performed a similar number of trials per session as naive mice. These results indicate that DLS DA is necessary for learning the task and forming stimulus-choice associations.

Stimulation of DLS DA provided causal evidence for our hypothesized role of DLS DA in learning. We hypothesized that stimulation of DLS DA signals at outcome time should only update the association between stimuli used for decisions and choices, leading to a reduction of DLS DA reward signals over trials and the emergence of DLS DA responses to these stimuli (Figure 4E). However, this stimulation should not update the association between choice and the stimuli that the animal does not use. Importantly, this is distinct from the learning effect of a classic RPE, e.g., triggered by extra water reward (Figure 4E). A classic RPE would update the association between all reward predictive cues and choices, having a learning effect on all trial types. To test this, we injected FLEX-ChrimsonR into the SNc of DAT-Cre mice (*n* = 5) and implanted optic fibers above DLS (Figures 4F and S7D). We first trained animals and selected mice that developed one-sided strategies. We then stimulated DLS DA axons at the outcome time of *incorrect* choices using 635 nm laser pulses (Figure 4G). We stimulated in incorrect choices because the range of psychometric changes for correct choices in expert animals is small due to ceiling effects. We performed this stimulation in trials with associated or non-associated stimuli in alternating days (Figure 4G, left column). The stimulation selectively influenced the associated side of the psychometric curve, decreasing the accuracy of choices in these trials (Figure 4G, upper row). In contrast, delivering water reward after incorrect choices in a similar experiment shifted the entire psychometric curve, decreasing the accuracy particularly on the flat side (Figure 4G, lower row). While DLS DA stimulation did not influence choices in zero-contrast trials, the reward delivery significantly affected those trials, lending further support for our conclusion. Therefore, distinct from the effect of water reward, the learning effect of DLS DA relies on the animal using visual stimuli for decision-making.

### A deep RL model with heterogeneous RPEs captures learning trajectories and dopamine signals

To understand the computational principles underlying the learning trajectories, DA signals and optogenetic effects, we designed several neural network models with different architectures and learning rules. We first outline a simple tractable deep network model that captured the data. We show that the model requires depth, i.e., multiple layers of tunable weights, and heterogeneous teaching signals to account for the data. We then demonstrate that the key features of our model generalize to larger networks.

**Figure 5:**
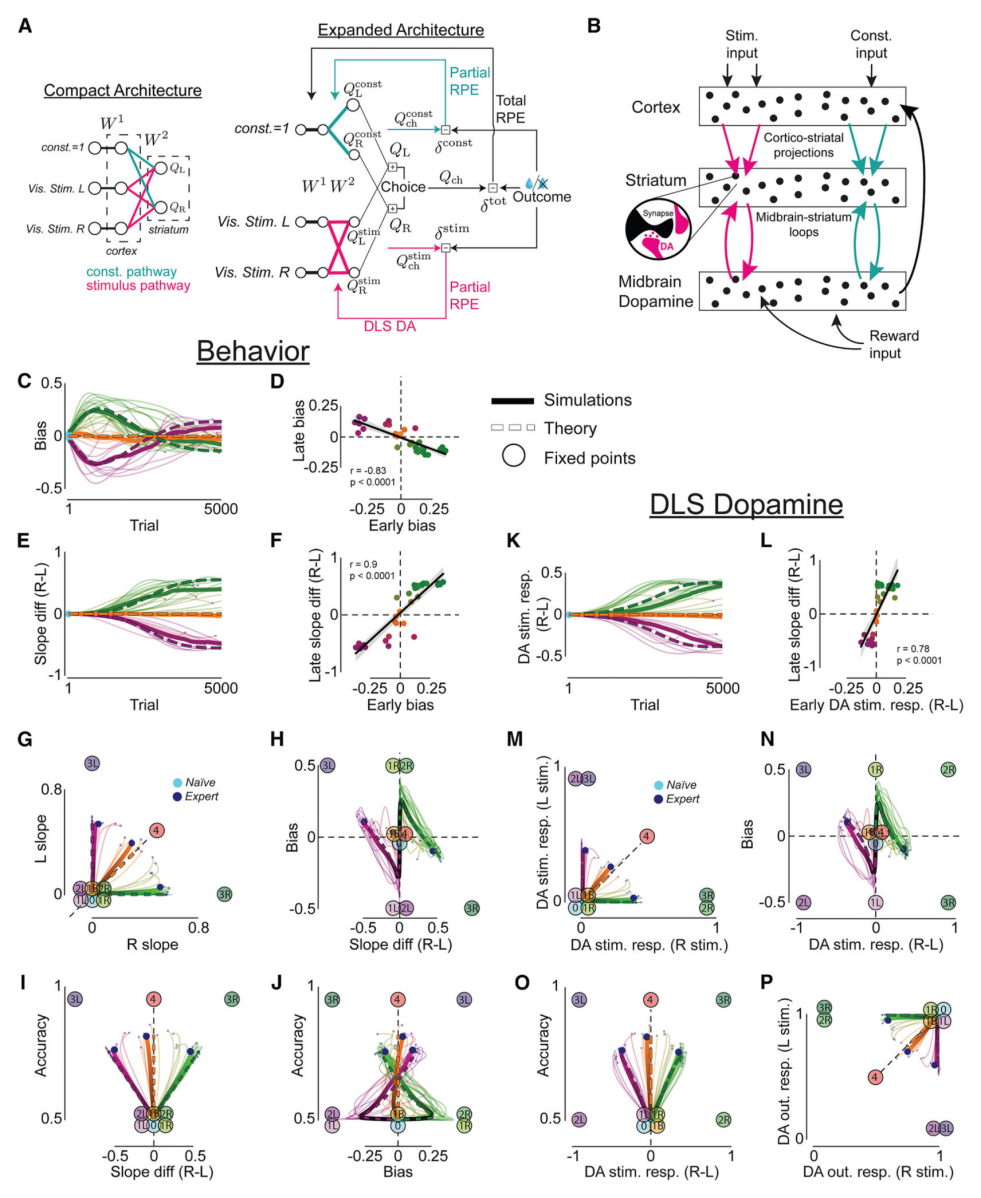
A deep RL tutor-executor network captures learning trajectories and DA signals (A) Compact (left) and expanded (right) schematic of the tutor-executor deep RL network and learning rule. (B) Diagram showing the proposed mapping between the model and brain anatomy. (C) Cf. Figure 1F, bias over trials per simulation (thin), for 3 clusters from (G) (thick), and for the average dynamics (thick dashed). Thick dashed lines in all panels indicate trajectories derived from the average dynamics (see STAR Methods). (D) Cf. Figure 1G, regression of early bias (trials 1,000–2,000) against late bias (final 1,000 trials). Each point represents a simulation. *p* value is calculated from the exact distribution of r. In all panels, shaded regions indicate 95% confidence interval across mice. (E) Cf. Figure 1H, difference between right and left psychometric slopes over trials per simulation (thin) and for 3 clusters from (G) (thick). (F) Cf. Figure 1I, regression of early bias against late slope difference. Each point represents a simulation. *p* value is calculated from the exact distribution of r. (G) Cf. Figure 1J, right vs. left slope over trials per simulation (thin) and for 3 clusters (thick). Clusters and colors obtained using the same procedure as for the behavioral data in Figure 1J. The clusters from this analysis are used in all other panels. Here and in (H)–(J), numbered circles represent fixed points of the learning dynamics (see Figure 7) plotted using the average behavior arising from their corresponding weight configurations. (H–J) Cf. Figures 1K–1M, in order, difference in right and left (R-L) slope vs. bias, R-L slope vs. accuracy, and bias vs. accuracy over trials per simulation (thin) and for 3 clusters from (G) (thick). (K) Cf. Figure 3A, difference in DLS DA responses to right and left stimuli (R-L) over trials per simulation (thin) and for 3 clusters from (G) (thick). (L) Cf. Figure 3B, regression of early difference in DLS DA responses to R-L stimuli against late slope difference. Each point represents a simulation. *p* value is calculated from the exact distribution of r. (M–P) Cf. Figures 3C–3F, in order, right vs. left DLS DA stimulus responses; difference in DLS DA responses to right and left stimuli (R-L) vs. bias; difference in DLS DA responses to right and left stimuli (R-L) vs. accuracy; and DLS DA rewarded outcome responses on right-stimulus vs. left-stimulus trials per simulation (thin) and for 3 clusters from (G) (thick). Fixed points are plotted using the average DLS DA responses arising from their weight configurations. See also Figures S8, S10, and S11.

The deep network model contains multiple layers of neurons that learn to predict the reward associated with each stimulus and choice. The network has an input layer, a hidden layer, and an output layer (Figure 5A; STAR Methods). To capture our DLS DA signals, and their learning effects in the optogenetic experiments, the network is organized into parallel pathways: the visual stimulus pathway (Figure 5A, pink) and the “constant” pathway encompassing all other inputs that do not vary across trials, e.g., auditory go cue (Figure 5A, aqua). A matrix of nonnegative weights denoted by *W*^1^ connects the input layer to the hidden layer, and similarly nonnegative weights in *W*^2^ connect the hidden layer to the output layer. The weights in *W*^1^ form one-to-one connections between input layer neurons and the hidden layer (“cortical”) neurons, resembling the anatomical segregation of visual inputs in each brain hemisphere. The weights in *W*^2^ are divided into two pathways, those connecting hidden layer neurons receiving visual inputs with the output layer, and those connecting the hidden layer neuron receiving constant input with the output layer. These resemble the brain’s segregated “cortico-striatal” projections (Figure 5B). The outputs of the network are two action values, *Q*_*L*_ and *Q*_*R*_, reflecting the learned value (i.e., reward prediction) of making each choice as a function of the inputs.

On each trial, the model receives inputs, makes a choice, and learns from the outcome. Two binary inputs “Vis. stim. L” and “Vis. stim. R” (represented as 0/1) indicate which of the stimuli is presented on a particular trial. The third input “constant” is always set to 1 to reflect stimulus-independent input, capturing environmental features that do not change trial-by-trial (e.g., auditory go cue). The model makes choices by comparing *Q*_*L*_ and *Q*_*R*_ using a softmax rule. The model then compares the outcome of the choice (reward/no reward) with its corresponding reward prediction to calculate RPEs used to update the weights *W*^1^ and *W*^2^. The model uses three different RPEs to update its weights. For *W*^1^, the RPE is calculated using a “total” reward prediction using all the inputs (*Q*_*ch*_). For *W*^2^, the updates differ for the two pathways (Figure 5A, pink and aqua arrows, respectively). The RPE in the stimulus pathway is calculated using a partial reward prediction $\left(Q_{ch}^{stim\;}\right)$ based on the stimulus inputs (*Vis stim. L* and *R*), whereas the RPE in the constant pathway is calculated using a prediction based only on the constant input $\left(Q_{ch}^{const\;}\right)$. The stimulus pathway’s reward prediction and RPE is the model’s account of DLS DA stimulus and outcome responses respectively (Figures 5A and 5B). This learning rule yields updates that minimize three different losses through gradient descent: the cortical loss equal to the total RPE^2^, and the two “cortico-striatal” losses equal to the pathway-specific RPE^2^s (STAR Methods). We term this model the “tutor-executor” network because the cortical learning (*W*^1^) tutors downstream cortico-striatal learning (*W*^2^) by determining the relative salience of the inputs and balancing updates in the executor pathways to minimize its total loss.

The model captured the diverse learning trajectories across mice. Similar to mice, the model started learning by developing varying degrees of left/right bias (Figures 5C and 5D cf. Figures 1F and 1G). Subsequently, the model’s biases reversed as its psychometric slopes grew, reproducing the diversity of left/right slope differences seen across mice (Figures 5E and 5F cf. Figures 1H and 1I). Further, the model’s bias early in learning predicted bias and psychometric slopes later in learning (Figures 5D and 5F cf. Figures 1G and 1I; Figure S8A cf. Figure S1N). Thus, the model’s entire learning trajectories resembled the diverse yet systematic trajectories of mice and exhibited similar sigmoidal accuracy curves over comparable timescales (Figures 5G–5J cf. Figures 1J–1M; Figure S8B cf. Figure S1C; Figures S8C–S8Ecf. Figures 1B–1D). The model’s simplicity allowed us to derive expressions for its average learning dynamics (STAR Methods), which showed close proximity to our behavioral data and model simulations (Figures 5C, 5E, and 5G–5J, thick dashed lines).

DLS DA responses over learning were also well captured by the model. We derived expressions for the trial-by-trial DA responses to stimuli and outcomes using reward predictions and RPEs from the stimulus pathway (STAR Methods). The partial prediction and RPE of the stimulus pathway best captured the DLS DA responses, compared with the constant and total predictions and RPEs (Figure S8F cf. Figure 2D). Analogous to the data, model-derived DA responses to stimuli grew over learning, reflecting the stimulus-choice associations that each animal formed (Figure 5K cf. Figure 3A; Figure S8F cf. Figure 2D). The model-derived DA signals early in training predicted both the model’s slope difference and DA signals late in training (Figures 5L, S8G, and S8H), akin to DLS DA data (Figures 3B, S6I, and S6J). Thus, model-derived DA responses to stimuli across learning exhibited the diverse yet systematic progression of empirical DA signals (Figures 5M–5O cf. Figures 3C–3E). Finally, model-derived DA responses to outcome also showed strong similarity to our data, encoding the difference between the outcome value and the reward predicted by the stimulus, i.e., RPEs in the stimulus pathway (Figure 5P cf. Figure 3F; Figures S8I–S8K cf. Figures S6K–S6M).

**Figure 6:**
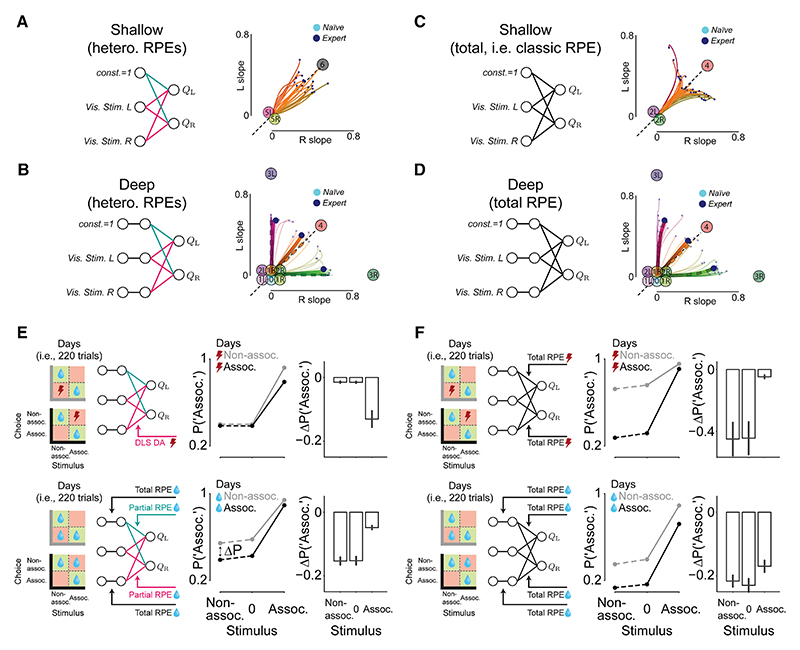
Shallow networks and models without heterogeneous RPEs do not capture learning trajectories and optogenetic results (A) Compact architecture of a shallow version of the tutor-executor network (left), and right vs. left psychometric slopes over trials for simulations of this network (right). Trajectory colors obtained from the average slope asymmetry as in Figure S1L. Numbered circles represent fixed points of the network learning dynamics (see Figure S8L). (B) Compact architecture of the deep tutor-executor network (left), and right vs. left psychometric slopes over trials for simulations of this network (reproduced from Figure 5G). (C and D) Compact architecture and right vs. left psychometric slopes over trials for shallow and deep versions of the single-loss gradient descent network, including the fixed points of each model (see Figures S8N and S9). (E) Schematic and behavioral results of simulating the DLS ChrimsonR optogenetic experiment with the tutor-executor network. Left, schematic showing the alternating block structure and effect of DLS DA stimulation (top) and total (i.e., classic) RPE manipulation on the model’s learning rule (bottom). Right, cf. Figure 4G, average psychometric curves, and their difference, on blocks with DLS DA stimulation (top) and reward delivery (bottom) on error trials with non-associated (gray) and associated (black) stimuli. Error bars indicate ± SEM across simulations. (F) Schematic and behavioral results of simulating the DLS ChrimsonR optogenetic experiment with the single-loss gradient descent network. See also Figures S8 and S9.

Without a deep architecture, standard shallow RL models with fixed state representations do not capture the mice’s diverse learning trajectories. We simulated learning in a shallow version of the tutor-executor network and found that its behavioral and neural trajectories did not display the characteristic one-sided strategies of mice, instead displaying trajectories similar to those of balanced mice (Figure 6A). The accuracy curve over learning of the shallow model also differed significantly from that of mice, lacking the initial plateau around 50% accuracy (Figure S8M). On the other hand, trajectories of the deep model reproduced the stages and diversity of mice learning trajectories and DLS DA signals (Figure 6B).

Explaining our optogenetic results requires the deep RL model to learn from heterogeneous RPEs. We tested two different learning rules for the model: one that included different RPEs for different pathways (Figures 6A and 6B) and another that only had a single total RPE (Figures 6C and 6D). For deep networks, both learning rules capture behavioral learning trajectories (Figures 6B, 6D, S8, and S9). However, only the model with heterogeneous RPEs could capture the differential effects of DLS DA and water manipulations (Figures 6E and 6F cf. Figure 4G).

Having established that our tutor-executor deep network model with *W*^1^ one-to-one connectivity accounts for our data (Figure 5), we then explored the constraints of the network and its generalization to larger networks. First, we explored the effects of network connectivity constraints. A fully connected network could not replicate the data. However, a model in which the visual stimulus pathway was fully connected captured the results (Figures S10A–S10C). Second, we relaxed the non-negativity constraint on network weights, showing that while the one-to-one network can still capture the trajectories, a network with full connections in its stimulus pathway develops only balanced trajectories (Figures S10A and S10B). Exploring the effect of these constraints on a network trained using a single total RPE gave similar results (Figures S10D–S10F). Third, we examined the effects of network initialization. Initializing *W*^1^ of the one-to-one tutor-executor network with the weights of trained one-sided model simulations did not capture the learning trajectories, i.e., the late slope difference did not depend on the side of the early bias (Figures S11A–S11D). Further, manipulating the initialization of a network with full connections in its stimulus pathway gave rise to diverse learning trajectories, with larger connectivity between stimulus inputs leading to more one-sided trajectories. However, this initialization effect did not fully replicate the bias reversal (Figure S11G cf. Figure 1G), nor the relation between early bias and late psychometric slopes (Figure S11H cf. Figure 1I) or DLS DA signals (Figure S11I cf. Figure 3C). Lastly, a larger network trained using pixel-level visual stimuli captured diverse mouse learning trajectories (Figures S10G–S10I), indicating that our results generalize to larger models commonly used in machine learning.

### Saddle points of the deep RL model explain learning trajectories and DA signals

We analyzed the deep tutor-executor network model to discover the mechanism that allows it to capture behavioral and neuronal data. To do so, we treated the deep RL model as a dynamical system and studied its average learning dynamics across inputs and choices.

**Figure 7:**
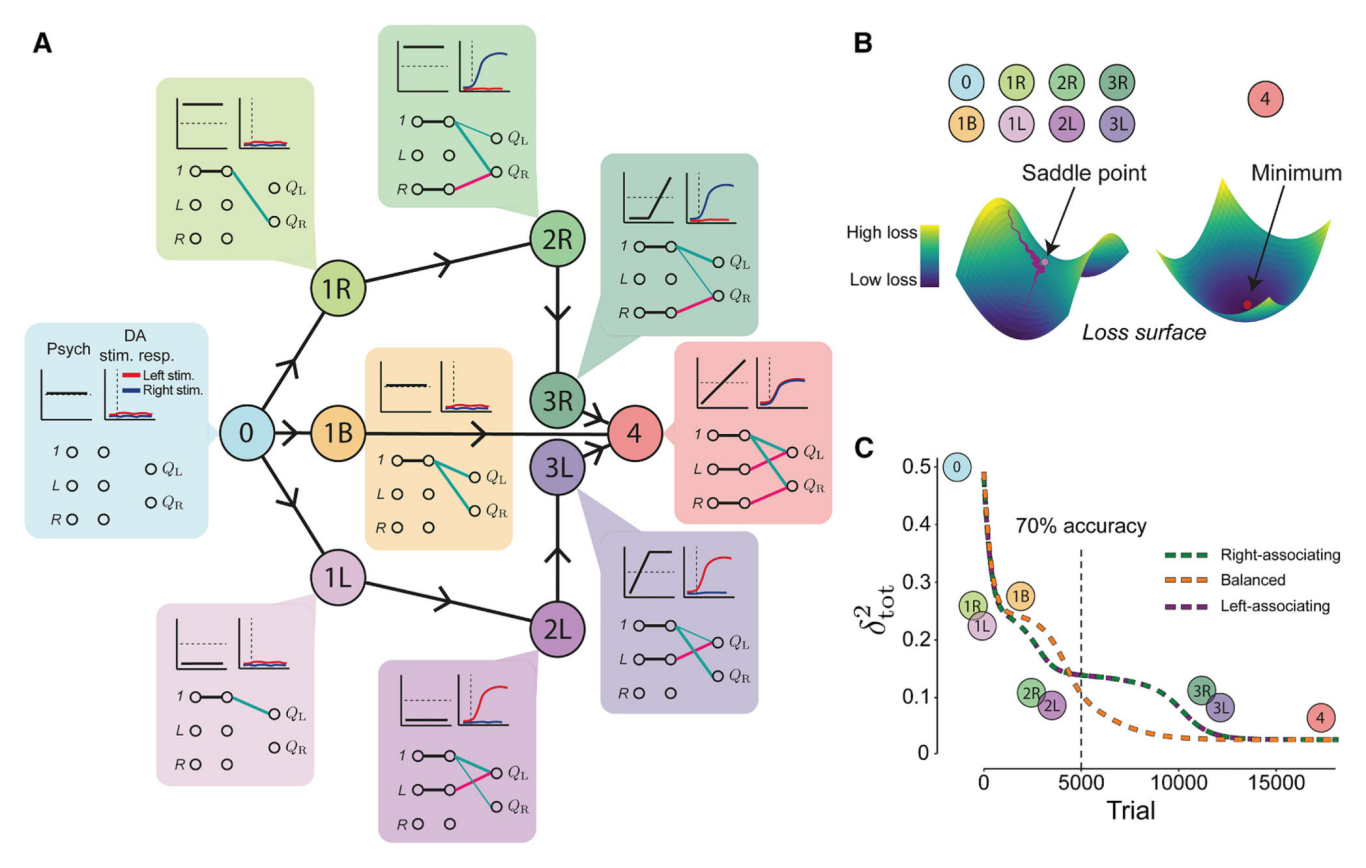
Saddle points of the deep RL model explain learning trajectories and DA signals (A) Schematic of the fixed point structure including behavioral and neural predictions, and corresponding network weight configurations. The connecting lines with arrows represent the steepest heteroclinic orbits into/out of each fixed point (see STAR Methods). All the fixed points are saddle points except for 4, which is the global minimum. (B) Schematic of the loss surface around a saddle point and a minimum, along with the classification of each model fixed point. (C) Average dynamics of the total RPE^2^ over learning for each of the clusters from Figure 5. Fixed points are plotted at approximate positions to depict their influence on the dynamics. See Figure S9 for a fixed point analysis of the single-loss gradient descent deep network. See also Figures S9, S11, and S13.

Analysis of the model’s average dynamics revealed a hierarchy of saddle points that explained the behavioral and neuronal trajectories (Figure 7A). We found these saddle points by deriving the fixed points of the average dynamics (i.e., weight configurations where the average update across inputs and choices goes to zero; STAR Methods). Saddle points have both stable and unstable manifolds, and in their vicinity learning momentarily slows down (Figures 7B and 7C). These points span the entire learning process, starting from a “naive” weight configuration (0) and converging on a final “expert” global minimum (4), and reflect the diversity of trajectories across mice. The saddle points establish a systematic flow through the parameter space, whereby points approached early in learning influence those approached later in learning.

Each fixed point has a characteristic behavioral and dopaminergic signature, similar to mouse behavioral strategies and DLS DA responses. Simulations start close to the first saddle point (0 in Figure 7A), corresponding to a network configuration with all weights set to zero. Next, simulations that, for example, develop an early right-side bias learn in the direction of the 1R saddle point, developing a strong association between the constant input and *Q*_*R*_, evident in its corresponding weight configuration diagram (1R in Figure 7A). These simulations then move preferentially toward the next saddle point, 2R, developing an association between the right stimulus and *Q*_*R*_ while maintaining a strong right bias. This happens as the simulation is still making mostly right choices (weight between constant and *Q*_*R*_ is larger than between constant and *Q*_*L*_) but starts to learn the correlation between right stimuli and reward after right choices. As this correlation is learned, the association between the constant input and *Q*_*R*_ weakens through small negative total RPEs that correct overpredictions of the reward on right-stimulus trials. This encourages left choices in the absence of the right stimulus, such that simulations approaching 2R move toward 3R, where psychometric slopes emerge, and the bias starts to reverse. Here, the simulation maintains its association between the right stimulus and *Q*_*R*_ while developing a strong weight between constant and *Q*_*L*_, without using the left stimulus. Thus, in the vicinity of this saddle point the simulation infers correct left choices from the *absence* of the right stimulus, showing psychometric slopes and DA signals similar to expert “right-associating” mice (green cluster in Figures 1, 2, and 3). A mirror image of this trajectory is observed in simulations that develop an early left bias (follow 1L, 2L, and 3L), whereas more balanced simulations move from 1B toward 4, i.e., the global minimum. As such, the saddle points of the deep RL tutor-executor network govern the learning trajectories (see saddle points visualized in Figures 5G–5J and 5M–5P), explaining their diverse yet systematic transitions between strategies and corresponding DA signals.

The saddle points of a deep network model substantially influence its learning dynamics. First, it governs the systematic transitions between strategies during learning, defining the relation between early and late behavior. Further, the multiple layers of the deep network give rise to saddle points that are critical for explaining learning trajectories, in particular 3R/L, that captures mice’s one-sided strategies. These saddle points emerged in our tutor-executor deep RL network and networks trained with standard gradient descent (Figures S8 and S9). In contrast, the saddle point structure of shallow models lack these one-sided saddle points and succinctly demonstrate why the shallow model cannot capture mice’s learning trajectories (Figures 6A, 6B, and S8L–S8O). Second, saddle points give rise to learning plateaus, where learning is slower. This can be observed in the dynamics of the model’s total RPE over learning, which exhibits plateaus in the vicinity of the saddle points (Figure 7C). Further, in the deep model, the 0 and 1 saddle points capture the initial plateaus in accuracy observed in mice (Figures S8M and S8O cf. Figure 1B). These plateaus also explain why some mice did not learn despite sufficient training (Figures S1O and S1P). Similar to these mice, trajectories of model simulations that did not learn stayed in close vicinity of 1L/R or 2L/R saddle points (Figures S11J and S11K).

## Discussion

In this work, we showed that mice learning from naive to expert display diverse yet systematic transitions through behavioral strategies. DA signals acted as teaching signals shaping learning trajectories. These signals reflected the intermediate strategies mice adopted from naive to expert, encoding the stimulus-choice associations determined by each individual’s strategy. Consistently, optogenetic manipulation of these DA signals altered each individual’s behavior, in a manner distinct from the effect of water reward. We tied these behavioral and neural results together through a deep neural network model trained using heterogeneous DA-like teaching signals, which reproduced the distribution of mouse learning trajectories, DLS DA signals and their optogenetic manipulations. The learning trajectories were qualitatively governed by saddle points and their connecting manifolds, providing a formal account for how a biological learning mechanism can steer and yield diverse yet systematic long-term learning trajectories.

### Individual diversity in long-term learning

Unlike conventional shaping methods that gradually change the task from easy to difficult, we maintained the full (i.e., relatively difficult) task throughout the experiment. Given that this procedure might have slowed down learning, many of our mice did not reach asymptotic expertise often used in studies of decision-making. Nevertheless, our results hold in animals with very high performance and graded levels of stimuli. Our training procedure allowed mice to explore and self-define their trajectories, facilitating individual diversity. This also allowed us to demonstrate that learning occurs in stage-like transitions. In fact, the substantial early side biases, and later one-sided strategies we observed could be due to the higher starting difficulty of our task. The effect of task difficulty in developing biases and one-sided strategies has been observed with other sensory modalities.^42^ Future studies can examine the effects of various shaping methods on learning trajectories.

Past studies have shown individual diversity in measures such as speed of learning across animals.^43,44^ Our results reveal that despite such individual diversity, learning trajectories can be highly systematic. In our data and model, the early variability in side bias emerges from uneven learning due to imbalances in the number of rewards after left and right choices, and not day-by-day variation in animals’ position. Consistently, analyses of eye movement and pupil size suggested that diversity in early side biases cannot be attributed to uneven detection of visual stimuli. However, these early side biases may be influenced by factors such as the mice’s initial position in the experimental rig and handedness. Beyond the effect of early side biases, different levels of confusion about the position of the stimuli in initial and early days might also contribute to one-sided learning trajectories.

### Dopaminergic mechanisms for long-term learning

We found that DLS DA reflected key characteristics of a teaching signal shaping individual trajectories. Two such characteristics were that it reflected both the evolving sequences of strategies and the diversity of learning trajectories across mice. Crucially, our model demonstrates that these DLS DA signals encode RPEs based on only a subset of task-relevant cues, i.e., the stimuli that animals use to make choices. This results in DLS DA reflecting the stimulus-choice associations corresponding to each strategy adopted throughout learning. This encoding persisted in highly trained mice and also guided trial-by-trial learning during asymptotic expert behavior, as our experiments with reward value manipulation showed (Figure S6H). The DLS DA signals differ from a total RPE that incorporates all task-relevant cues to compute reward predictions. The “partial” stimulus-based RPE we observe in DLS DA signals could arise from the heterogeneous topography of cortico-striatal connectivity (Figure 5B). If so, our model implies that there should be different DA signals across striatal regions, as demonstrated here and in previous studies.^22,23,30,45,46^ For instance, our DLS DA signals were bilateral, in contrast to DMS DA signals, which showed responses only to contralateral stimuli here and previously.^47^ This difference could be due to DLS receiving more bilateral inputs from frontal association cortical areas compared with DMS.^48^ The DLS DA signals we recorded modulate the strength of local cortico-striatal synapses.^32^ This could rapidly regulate the size of stimulus-evoked responses in striatal neurons which form a functional closed loop by projecting back to midbrain DA neurons^49–51^ (Figure 5B), causing the changes in DA stimulus and outcome responses we observed throughout learning.

Optogenetic experiments showed that DLS DA is necessary for learning the task and dissociated the effect of stimulus-based partial RPEs from total (i.e., classic) RPE on learning. Our behavioral and DLS DA results showed that a deep—not shallow—RL model is required to account for the trajectories but did not differentiate between learning from heterogeneous RPEs vs. a single total RPE. However, our optogenetic results distinguished these, showing that the deep model requires heterogeneous teaching signals to account for the distinct learning from DLS DA signals (i.e., partial RPE) compared with reward size manipulation (i.e., total RPE). Our results thus show that heterogeneous DA signals are at the service of learning. From the perspective of calculating RPEs based on a subset of inputs, our model is similar to a recent study.^52^ Critically however, unlike our model, their model sums heterogeneous RPEs to provide a “globally broadcasted” RPE (akin to our total RPE) for updating all weights into striatum. Other RL models, such as distributional RL, attribute DA heterogeneity to diverse sensitivities to positive and negative RPEs.^53^ Such a model does not capture our data because despite choices with associated and non-associated stimuli having similar reward distributions when accuracies are matched, DLS DA signals differed substantially in these trials. Lastly, our deep RL model implicates DA in discovering states relevant to rewards, reminiscent of other models of causal learning.^54^

Signatures of stimulus-choice association emerged in RTs before being evident in choice accuracy. DLS DA stimulus signals reflected this early signature, emerging precisely when mice showed signatures of using visual stimuli, i.e., when mice had a flat psychometric curve and choices for stimuli were becoming faster. Past studies have shown DA responses aligned to reward-seeking movements or even spontaneous movements.^55,56^ Although the precise temporal evolution of DA signals might depend on the task,^21,57^ our results suggest that DLS DA signals were better locked to stimulus onset than choices. Nevertheless, these pre-outcome DA signals could contribute to reducing RTs by invigorating action^26,58^ (Figure S6N). The DLS and DMS DA signals also differed from previously reported DA novelty signals observed during initial exposure to stimuli.^21,39,40,59^ These signals were absent in initial days and grew over time, with DMS DA showing slightly faster growth over days than DLS DA (Figures S12A and S12B). The absence of DA novelty signals could be because the visual stimuli we used were not salient enough in the initial stages of learning, before their task-relevance was discovered. DMS DA did not respond to reward delivery, and consistent with previous studies,^47^ did not reflect reward size (Figure S6H), suggesting that its role in learning differs from that of a RL RPE. Optogenetic manipulation of DMS DA signals at stimulus time led to contralateral biases developing over days (Figures S12C–S12E). However, the absence of DMS DA stimulus responses in initial days suggests that DMS DA might not contribute to the early biases we observed in mice.

### A mathematical framework for long-term learning

Our experiments highlighted two hallmarks of long-term learning: its systematic stage-like progression through strategies and its marked individual diversity. Many other well-studied abilities such as semantic cognition and navigation are characterized by similar structured transitions through strategies.^60–62^ However, the source of individual differences in learning has been difficult to understand. Our model reveals that both hallmarks could arise from a single dynamical process approaching saddle points in the reward landscape of a deep neural network. As the learning dynamics approach a saddle point, learning momentarily slows down until the saddle point’s unstable manifold is discovered.^63,64^ The dynamics then speed up as gradients grow, following the steepest path to the next saddle point in the sequence. This alternation between slow and fast learning along sequences of saddle points explains the systematic stage-like transitions during long-term learning. These transitions are evident in the average dynamics of the model’s total RPE (Figure 7C), which resembles mice RTs over learning (Figure S1I). Moreover, the divergence in the sequences of saddle points demonstrates how choices made early in learning influence strategies developed later on and explains individual diversity. Consistently, the trajectories of mice that failed to learn remained in the vicinity of early saddle points (1L/R or 2L/R). Finally, the model demonstrates that depth is a requirement of the circuit architecture without which saddle points, and hence the characteristic learning stages and their diversity, do not emerge. An intriguing feature of the tutor-executor learning dynamics is that, with extensive training, weight magnitudes transfer from *W*^1^ (cortical) to *W*^2^ (cortico-striatal) while maintaining the value of their product *W*^*2*^*W*^*1*^ (Figure S13). This resembles previously observed “transfer to striatum,” where cortex is crucial for early stages of learning.^65^ It also explains past results involving DLS in habits,^66^ as the decrease in *W*^1^ leads to lower learning flexibility. This work shows how saddle points are signatures of DA driven learning in “deep” cortico-striatal circuits, which could generalize beyond our task.

### Limitations of the study

The temporal resolution at which we observed systematic trajectories was on the order of hundreds of trials. However, there could also be behavioral strategy switches happening on faster timescales,^67^ which may be consistent with our observations. We did not measure DA signals in other DA-rich regions such as ventral or posterior striatum nor frontal cortex, but we speculate that they might encode other prediction errors of our model. Lastly, while other learning rules might account for some aspects of our data, our model explains the results using a simple gradient descent learning rule.

### Resource Availability

#### Lead contact

Further information and requests for methods and analyses should be directed to and will be fulfilled by the lead contact, Armin Lak (armin.lak@dpag.ox.ac.uk).

#### Materials availability

This study did not generate new unique reagents.

## Star★Methods

### Key Resources Table

**Table:** 

REAGENT or RESOURCE	SOURCE	IDENTIFIER
Bacterial and virus strains		
pAAV-hsyn-GRAB_DA2m	Addgene	Cat#140553; RRID:Addgene_140553
pAAV-Syn-FLEX-rc[ChrimsonR-tdTomato]	Addgene	Cat#62723; RRID:Addgene_62723
pAAV-hSyn1-SIO-eOPN3-mScarlet-WPRE	Addgene	Cat#125713; RRID:Addgene_125713
pAAV-Ef1a-DIO-ChRmine-mScarlet-WPRE	Addgene	Cat#130998; RRID:Addgene_130998
Deposited data		
Data to reproduce figures in paper	Figshare	https://doi.org/10.6084/m9.figshare.28877912
Experimental models: Organisms/strains		
Mouse: C57BL/6J	The Jackson Laboratory	#Cat000664;RRID:IMSR_JAX:000664
Mouse: B6.SJL-*S/c6a3*^*tm1.1(cre)Bkmn*^\J	The Jackson Laboratory	Cat#006660;RRID:IMSR_JAX:006660
Software and algorithms		
Code to reproduce figures in paper	Figshare	https://doi.org/10.6084/m9.figshare.28877942
MATLAB (2019a for Rigbox)	MathWorks	https://www.mathworks.com/
Rigbox (modified version)	CortexLab (modified by Armin Lak)	https://github.com/ArminLak/Rigbox
Python (3.12)	Python	https://www.python.org/
Bonsai (2.8.1)	Bonsai Foundation	https://bonsai-rx.org/
JAX (0.4.34)	The JAX Authors	https://docs.jax.dev/en/latest/
Pytorch (2.5.1)	Pytorch Foundation	https://pytorch.org/
Fiber localization based on allenCCF	CortexLab	https://github.com/cortex-lab/allenCCF
Other		
Fibre Photometry System (FP3002)	Neurophotometrics	https://neurophotometrics.com/fp3002
Mono Fiber-optic Patchcord	Doric Lenses	MFP_200/220/900-0.37_2m_FC-MF1.25
Low Noise Diode Laser	Shanghai Dream Lasers Technology	SDL-532-LN-100MFL
Optic Fiber Cannula (200um core)	Neurophotometrics	https://neurophotometrics.com/cannulae-and-sleeves
Stereotaxic drill robot	Neurostar	https://robot-stereotaxic.com/drill-robot/

### Experimental Model and Study Participant Details

#### Mice

The data presented in this paper was collected from 65 (30+10+5+7+7+6) male wild-type C57/BL6J mice and DAT-Cre mice, with their age ranging between 9 to 30 weeks. The first 30 mice were wild-type mice that were trained on the learning experiment from naÏve to expert (i.e., learner mice). The next 10 mice were also trained on the task, but did not reach expertise despite more than 4300 trials of training (i.e., non-learner mice). The next 5 were DAT-Cre mice where eOPN3 virus was expressed for the inhibition of dorsolateral striatum dopamine. The next 7 were DAT-Cre mice where ChrimsonR virus was expressed for the stimulation of dorsolateral striatum dopamine. The next 7 were DAT-Cre mice where ChrimsonR virus was expressed for the stimulation of dorsomedial striatum dopamine. The last 6 were wild-type mice that were trained with shaping, more contrast levels and more extensive training to higher accuracy levels. All experiments were conducted according to the UK Animals Scientific Procedures Act (1986) under appropriate project and personal licenses.

#### Surgical procedures

Animals were anaesthetized with isoflurane and were kept on a feedback-controlled heating pad (Stoelting 53810). Hair overlying the skull was shaved and the skin and muscles over the central part of the skull were removed. The skull was thoroughly washed with sterile saline. A head plate was attached to the bone posterior to bregma using dental cement (Super-Bond C&B). After the head plate fixation, we made craniotomies over the target areas and injected 300nl of AAV9-hsyn-DA2m (for recording dopamine, titer 5×10^12^ genome copies/ml) into the right and/or left DLS (AP: +0.5mm from bregma; ML: +/-2.5mm from midline; DV: 2.8mm from dura) or DMS (AP: +1.25mm; ML: +/-1mm; DV: 2.8mm). For the optogenetic experiments, either 400nl of 4.1pAAV-hSyn1-SIO-eOPN3-mScarlet-WPRE (inhibition experiment, titer ~4.67×10^12^ genome copies/ml) or 150nl of 3.2pAAV-Syn-FLEX-rc[ChrimsonR-tdTo-mato] (stimulation experiment, titer ~1.67×10^12^ genome copies/ml) was injected into the right and left SNc (AP: -3mm; ML: +/-1.5mm; DV: -4.3mm) of DAT-Cre mice. The injections were performed slowly over 20 minutes using Nanoject II (Drummond). This was followed by implantation of the optical fiber over the DLS or DMS (core = 200 um, Neurophotometrics Ltd), which was secured to the head plate and skull using dental cement. Mice recovered for at least seven days following the surgery. We waited for an additional 8 weeks for opsin expression in mice with eOPN3 injections and 4-5 weeks in mice with ChrimsonR injections.

## Method Details

### Behavioral task

We trained mice in a complete psychometric visual decision-making task from day 1 until expertise. Following surgery recovery, mice were first habituated to the experimenter for 2-3 days, followed by 2-3 days of habituation to the experimental rig and head-fixation. In each day of the experiment, mice were head-fixed with their body and hind-paws resting on a stable platform with a covering, and their forepaws resting on a steering wheel that could be rotated left and right. Each trial began after the wheel was held still for a short quiescence period (t1=0.7-0.8s) (Figure S1A). A sinusoidal grating stimulus of varying contrast (0%, 25% and 50%) was presented on either the left or right side of a screen 10cm in front of the mouse, followed by an auditory go cue t2=0.2s after visual stimulus onset. The go cue indicated the start of the interactive period, during which wheel movements were coupled with movement of the visual stimulus on the screen. The mouse was required to indicate the position of the stimulus within a limited response time (t3=30s) by steering the wheel in the correct direction to move the stimulus to the center of the screen, causing a water reward (3ul drop) to be delivered via a spout positioned close to the mouth. While the stimulus was held in the center of the screen for t4=1s, a variable ‘feedback delay’ (t5=0.1-0.3s) separated the time of choice completion from reward delivery. Subsequently, the next trial started following an ‘inter-trial delay’ (t7=2-3s). When an incorrect choice was made, the mouse was presented with a t6=0.5s white noise auditory stimulus feedback via speakers positioned near each ear and had a brief timeout period (t8=2s) before the next trial. When a mouse responded incorrectly to an ‘easy’ high-contrast stimulus (50% contrast), there was a 50% chance that the same stimulus was repeated in the next trials, until the mouse responded correctly. Mice were trained for approximately 30 minutes each day. In 6 mice (Figure S6A), we gradually included stimuli with lower contrasts throughout training to have stimuli with contrast of 0%, 6%, 12.5%, 50% and 100%, and trained them to high performance (~85-90%). The behavioral experiments were delivered by custom-made software written in MATLAB (MathWorks) which is freely available.^68^

### Reward size manipulation experiment

In a subset of trained mice with dopamine recordings in DLS/DMS, we investigated the effect of reward size on choices and DA signals with our behavioral task (Figure S6H), similar to previous studies.^58^ To do so, we provided twice as much water reward for correct choices after left or right stimuli (3ul vs. 1.5ul) in alternating days of testing. This alternation was performed for at least 6 days in each mouse (i.e. 3 days per condition).

### Imaging dopamine release

To measure dopamine (DA) release in the dorsolateral striatum (DLS), we employed fiber photometry. Photometry and behavioral data were collected simultaneously. We used chronically implanted optical fibers to deliver excitation light through patchcords (Doric Lenses) and collected emitted fluorescence (Neurophotometrics FP3002). We used multiple excitation wavelengths (470 and 415nm), delivered on alternating frames (sampling rate of 40 Hz), serving as target and isosbestic control wavelengths, respectively.

The recorded photometry signal was pre-processed following steps described previously.^69^ We began by de-interleaving the recorded signal at 470nm and 415nm wavelengths. Both signals were then de-noised to remove short-pulse artefacts using a median filter with kernel size 5 (medfilt from scipy.signal). Subsequently, the signals were detrended with a zero-phase low-pass filter with a 10Hz cutoff frequency (2nd-order butterworth filtfilt from scipy.signal). Next, a photobleaching correction was applied to remove slow changes in the signal likely coming from fluorophore degradation due to light exposure throughout the recording session. To do this, we used a scipy filtfilt zero-phase high-pass filter with a cutoff frequency of 0.001Hz, thus removing signals varying with a timescale slower than 16 minutes. We then corrected for motion signals by fitting the 415nm isosbestic to the 470nm signal with a least squares polynomial fit of degree 1 (linregress, scipy.stats) and the resulting fitted signal was then subtracted from the 470nm signal. Finally, this quantity (ΔF) was normalized through division by the baseline fluorescence (F, defined as a low-pass filtering of the denoised 470nm signal with a cutoff frequency of 0.001Hz) to obtain ΔF/F which was subsequently z-scored per session to enable more accurate comparisons across days of recording.

High data quality was ensured by removing sessions with weak DA signals. We plotted the relative amplitude of the raw 470nm and 415nm signal per session. If this ratio was smaller than 1 the session was discarded, since in such sessions most of the pre-processed ΔF/F fluctuations came from variation in the isosbestic signal instead of the informative 470nm channel. We also discarded sessions where the maximum fluctuations were smaller than one standard deviation (i.e., Z<1).

### Optogenetic manipulation of dopamine signals

#### DLS DA inhibition experiment

Mice expressing the inhibitory opsin eOPN3^41^ were trained on the same visual decision-making task described above, following the same recovery and habituation protocol. Throughout each day of the experiment, laser pulses (each 600ms, 532nm, ~12mW measured at the tip of the patch cord; Shanghai Dream Lasers Technology) were delivered through the optical fibers in random intervals of 6-14s, independent of task trials. Mice were trained for approximately 30 minutes each day for a total of at least 4300 ‘good’ trials (i.e., after preprocessing, see section below).

#### DLS DA stimulation experiment

After similar recovery and habituation protocols to those described above, we trained mice expressing the excitatory opsin ChrimsonR in the DLS dopamine terminals. Upon reaching a stable accuracy above 70%, we began the optogenetic experiment. In alternating days, laser pulses (25ms on/25ms off, 635nm, 10mW measured at the tip of the patch cord) were delivered through the optical fibers over a period of 490ms at the outcome time of incorrect left (day n) or right (day n+1) choices (including non-rewarded zero-contrast trials). This alternation was performed for at least 8 days in each mouse (i.e., at least 4 days for each condition). As a control, a similar experiment was performed in expert animals with or without ChrimsonR, replacing laser stimulation with the delivery of a water reward. This experiment included a baseline day between manipulation days where mice performed the original task. This was done to avoid mice from becoming too biased towards one side. The protocol was run for at least 12 days in each mouse (i.e. at least 3 days of each left/right condition). One mouse from the DLS dopamine stimulation cohort expressed the excitatory opsin pAAV-Ef1a-DIO-ChRmine-mScarlet (i.e., ChRmine). The surgery was performed similarly to the other mice used for optogenetics, injecting 200nl of the virus (titer ~9×10^12^ genome copies/ml) into SNc and then implanting optical fibers over DLS. For stimulation, we delivered laser pulses (25ms on/25ms off, 532nm, 0.25mW measured at the tip of the patch cord) over a period of 490ms.

#### DMS DA stimulation experiment

Following the recovery and habituation protocols defined above, we manipulated DMS dopamine levels from naïve while training mice expressing ChrimsonR with unilateral DMS fibers on the original task. In a random selection of 75% of trials with the stimulus contralateral to the DMS fiber, laser pulses (25ms on/25ms off, 635nm, 10mW measured at the tip of the patch cord) were provided over a period of 200ms through the optical fibers, locked to stimulus onset. This stimulation was provided every day of the experiment for at least the first 5 days of training.

### Video monitoring

The left eye was monitored with a camera (Teledyne Flir CM3-U3-13Y3M-CS) fitted with a zoom lens (Thorlabs MVL50M23) recording at 20 Hz. Front body movements were monitored with another camera (same model but different lens, Thorlabs MVL16M23) also recording at 20 Hz. Mice were illuminated with infrared light (850nm, BW BWIR48) for the recording of eye and front body movements. Moreover, the box was lit by dim visible light so that mouse pupil is moderately dilated.

### Histology and fiber track quantifications

Histology was performed after the experiments to confirm successful fiber positioning. Animals were deeply anaesthetized and perfused using 4% paraformaldehyde (PFA) and then decapitated. The brains were extracted, left in 4% PFA for 24h to post-fix in a refrigerator and then embedded in blocks of 1.5% agarose gel before collecting slices at 70 um thickness using a vibratome (Leica VT1000 S). Slices were then stained with DAPI for 15 min (1:1000 solution), mounted onto glass, coverslipped, and imaged using an epifluorescence microscope (Leica).

## Quantification and Statistical Analysis

### Behavioral data analyses

#### Behavioral data pre-processing

The behavioral data was pre-processed by removing the following trials: trials with response times more than 2 standard deviations above the mean per session, repeat trials (trials repeated after high contrast incorrect trials) and trials where mice did not make a choice in less than 30s.

#### Behavioral metrics

The main behavioral metrics we used to analyze the mouse trajectories were accuracy, psychometric slope and bias. Accuracy was defined as the proportion of rewarded choices in all trials except for those without stimuli (i.e., zero-contrast trials), where choices were rewarded randomly. Psychometric curves were calculated per session, and plotted the proportion of rightward choices (i.e., P(‘Right’)) for different stimulus positions (left, right) and contrast values (0, 0.25, 0.5). The value of psychometric slope we used in all our analyses is that of the simplified psychometric curve collapsing across contrast levels to give left stim, zero-contrast and right-stim x-axis values. Left (right) slope was defined as the absolute difference in P(‘Right’) for left (right) stimulus and zero-contrast trials. Bias was defined as the difference between the P(‘Right’) on zero-contrast trials and 0.5, thus representing the imbalance of choices on zero-contrast trials in left and right directions.

#### Learning Trajectories

Each mouse was assigned a color and cluster based on its learning trajectory. The rule for assigning colors used a weighted average of the difference between right and left per-session slopes over learning, where the weighting was equal to the sum of the left and right slopes on each session (Figure S1L). The resulting average slope asymmetry metric was used to determine a color for each mouse on a spectrum ranging from purple (for negative values) to orange (for values around 0) to green (for positive values).

The trajectories of the behavioral metrics were smoothed for better visualization, highlighting their slow variation over learning. To do this, we used scikit-learn’s Gaussian process regression package^70^ to fit a gaussian process with an RBF kernel (with tunable scaling and length-scale) to the session-by-session metrics. The predict method of the fit gaussian process could then be used to estimate the smoothed value of the metric at different time points over learning.

A cluster label was assigned to each mouse to obtain cluster averages that highlighted the main trends in the diversity across learning trajectories. A dynamic time warping clustering algorithm was used to obtain these clusters.^71^ This algorithm first looks for the time warping that best clusters the trajectories by shape. The cluster centers are then computed as the barycenters with respect to the time warped mouse trajectories, yielding cluster centers that are similar in shape to the individual trajectories, thus solving the problem of averaging across mice that learn at different speeds. This clustering was applied to the smoothed right vs. left slope trajectories in Figure 1J and the resulting cluster labels were used to compute the cluster averages in all other behavioral and neural plots throughout the paper. The same coloring, smoothing, and clustering methods were applied to the model simulations to obtain plots similar to those produced for the experimental data.

#### Pupil analysis

We used DeepLabCut^72^ to track several points on the mice’s left pupil throughout each task trial. We selected 4 points in the top, bottom, left and right portions of each mouse’s pupil and recorded the x and y coordinates of each point over time. For our pupil motion analysis (Figure S2B), we defined the average x and y coordinate of these 4 points as the position of the pupil and investigated its horizontal (Δx) and vertical (Δy) motion. For the pupil dilation analysis (Figures S2A and S2B), we defined the pupil diameter as the mean of the Euclidean distances between the top & bottom and left & right points. The average x and y coordinates, as well as the pupil diameter measure, were z-scored and smoothed using a low-pass filter per session to enable more accurate comparisons across days. To reduce noise, we excluded sessions where the standard deviation of any of the non z-scored x-coordinates, y-co-ordinates or pupil diameter were above their 75th percentile. The alignment to stimulus onset and time warping was subsequently performed as described in the neural analyses section.

#### Lick analysis

We used FaceMap^73^ to track the mouth and lower lip key points on videos of the mice’s front body throughout each task trial (Figures S2A and S2B). We recorded the x and y coordinate of each key point and defined a mouth-lip (Euclidean) distance measure that was used to detect licks. Licks were detected by applying the scipy.signal.find_peaks function to the mouth-lip distance with a minimum required prominence of 1. A lick rate measure was subsequently defined using a moving average with a window size of 4, and was smoothed per session using a low-pass filter. The alignment to stimulus onset and time warping was subsequently performed as described in the neural analyses section.

#### Wheel movement analysis

Wheel motion was recorded using a rotary encoder acting as the wheel’s axel. The wheel position was saved in mm with a frequency of 200Hz, increasing for clockwise motion and decreasing for counterclockwise motion. The recordings were aligned to trial events and time warped as described in the neural analyses section.

### Neural data analyses

#### Event alignment and time warping

DLS dopamine recordings were aligned to task events that caused significant DLS dopamine release, as determined by a linear deconvolution (see below). These events were visual stimulus onset, choice completion (i.e., correct trials: visual stimulus arriving in the center of the screen, and incorrect trials: stimulus moving out of the screen) and trial outcome (reward/no reward). To do this, a fixed time period around each event (-0.5s to +1s) was selected and a fixed number of elements for the resulting aligned neural trace were chosen (i.e., 100). The DA recording was then linearly interpolated to obtain a value for each of the desired time points in the chosen time period (using scipy.interp1d). The average value in the time period before the event (-0.5-0s) was used as a baseline and subtracted from the event-aligned traces.

Time warping was used to visualize DA signals in a single continuous trace including all trial events. This was achieved by warping the DA signals such that a fixed number of time points represented the time course between each event. In this way, varying time periods between events were accounted for by allowing different time intervals between data points. We chose to have 40 time points before stimulus onset, 30 between stimulus onset and choice completion, 12 between choice completion and outcome and 60 post-outcome. The recorded DA signal was then interpolated to obtain the fluorescence values at the corresponding time points. This time warping allowed us to compute and visualize average DA signals across trials with varying durations.

#### Normalization across sessions

In addition to the pre-processing steps described above, we further corrected for session-by-session variation in fluorescence levels by normalizing the DLS DA signals to the peak of the average DLS DA response to reward delivery in zero-contrast (i.e., no visual stimulus) trials. This was done for all analyses except those comparing DLS and DMS DA signals, due to lack of a suitable reference for normalizing the DMS signal. To do this, we computed the average time warped DA trace from zero-contrast trials per session, took the mean of the 3 largest values in the post-reward response (peak), and then subtracted the mean fluorescence levels 3 time points before and after the time of reward delivery (baseline) to obtain a single number (peak – baseline) that was used to divide the entire DA recording from that session by. This normalization assumes that DA reward responses in zero-contrast trials should not vary session-by-session, as there are no cues that can predict the random reward delivery on these trials, meaning that the degree of ‘surprise’ (i.e., reward prediction error) should remain fairly constant throughout learning. This yielded normalized ΔF/F values that ranged from 0–1, expressing the signal as a proportion of the zero-contrast reward response in each session, thus resulting in more accurate averages across sessions and mice and more interpretable fluorescence values that could be compared with the model-derived DA signals.

#### Analysis windows

We defined average DA responses to 3 events (stimulus onset, choice completion and reward) which we use in several analyses throughout the paper. These were defined as the average DA signal in a specific time window after each event, relative to the signal before the event.

For the ‘stimulus response’, we defined an analysis window from +0.2s to +0.35s post-stimulus onset and took the peak response of a moving average (window size = 10) of the signal in that time window. We then subtracted a baseline fluorescence value (defined as the mean ΔF/F in a short window around stimulus onset) from the peak estimate to obtain the ‘stimulus response’ on each trial.

For the DA response to choice completion (‘stim. center’ on correct trials), we used the time warped traces to define the analysis windows for the peak and baseline estimates. We did this because the times between stimulus, choice completion and reward delivery/absence are variable and can be short, so using time warping is an accurate method to obtain isolated estimates of DA release values uniquely around this event. To do this, we used the entire 12 elements of the time warped trace between choice complete and reward delivery/absence and found the peak value of its moving average (window size = 6). From this, we subtracted the mean of the time warped DA signal one time point before and after the choice complete event.

For the DA response to reward delivery/absence we used the same procedure as for the ‘stimulus response’. We only changed the analysis window from which we calculated the peak response, i.e., 0-1s after the reward time. The baseline was similarly defined using the average signal in a window -0.1s to +0.1s around the event.

For most neural analyses we defined a combined ‘outcome response’ which is the addition of DA responses to choice completion and reward delivery/absence. This was done because in rewarded trials, the ‘stim. center’ cue is a perfect predictor of upcoming reward and thus rapidly acquires value, causing the DA responses to reward to become smaller. We did not want to include this learning process in our analyses as it does not involve decision making (i.e., is Pavlovian). Hence, by adding the responses for the two events, we obtained an outcome signal that did not change until events before choice completion became associated with reward.

#### Linear deconvolution

To find the events that caused significant variation in DMS and DLS DA release levels, we used a linear deconvolution algorithm. This algorithm works by regressing binary variables indicating the time period around events onto full trial DA signals. The design matrix (*X*) has a column per time point around the events of interest (regressors) and a row per time point of the DA signal, which was concatenated for all the trials used in the regression to form the dependent variable (*y*). The algorithm then finds the optimal scaling (β*) of the regressors in *X* which produces a prediction of the concatenated DA signal (ŷ*) that minimizes the mean squared error Σ_i_ *(y*_*i*_ *– ŷ*_*i*_*)*^*2*^,

(Equation 1) $$\beta^{*}={\left({\text{X}}^{⊤}\text{X}\right)}^{-1}{\text{X}}^{⊤}\text{y},$$

(Equation 2) $$\hat{\text{y}}*=X\beta *$$

The resulting elements in β* compose the deconvolved signal for each event. The benefits of using linear deconvolution over an event-aligned average is that it isolates the effect of each event on the DA signal, removing the influence of other events occurring shortly before or after. This isolation is achieved as long as there is enough jitter between events across trials; if two events are separated by a fixed delay in all trials the regression will not find isolated signals due to correlated regressors.

To account for changing DA signals over learning in our linear deconvolution, we split all trials into 4 bins with increasing psychometric slope. We also performed separate regressions for rewarded and unrewarded trials. The events we considered were stimulus onset, choice start, choice completion and reward delivery/absence (Figures S3B and S3G). The explained variance for each event was computed by comparing the 5-fold cross-validated R^2^ of a ‘full’ model with all the events against that of a model without the event being assessed.

We used a custom implementation of this algorithm written in Python, which can be found in our analysis code repository.

### Deep linear RL model

#### Architecture

The model is a 3-layer deep neural network with linear activation functions (Figure 5A). We denote the weight matrix connecting the input layer to the hidden layer *W*^1^, and the matrix connecting the hidden layer to the output layer *W*^2^, such that the function computed by the network is given by

(Equation 3) $$y=W^{2}\;W^{1}\;x$$

where *x* is the vector of inputs and *y* is the vector of outputs. The weights in both *W*^1^ and *W*^2^ are constrained to be nonnegative, and *W*^1^ is constrained to be a diagonal matrix. These constraints were chosen for simplicity and are not strictly necessary to capture the data. The network has two binary input neurons which encode the presence or absence of the left and right visual stimulus and one input neuron that has an activation of 1 for every trial, representing any non-stimulus cues that the mice may use to make choices on each trial, e.g. the auditory go cue. Thus, the network receives three different input vectors depending on the trial type,

(Equation 4) $$\text{Left}\;\text{stimulus}\;:\;\left[\begin{array}{l} 1\\ 1\\ 0 \end{array}\right],\;\text{Zero-contrast}\;:\;\left[\begin{array}{l} 1\\ 0\\ 0 \end{array}\right],\;\text{Right}\;\text{stimulus}\;:\;\left[\begin{array}{l} 1\\ 0\\ 1 \end{array}\right]$$

We opted not to model the different contrast levels on different trials (25% and 50%) because in most mice we did not see a significant difference in accuracy between these two levels.

In every trial, the input vectors are multiplied by the weights in *W*^1^ and *W*^2^ to obtain the activation of the two output neurons which encode the learned value of left and right choices, *Q*_*L*_ and *Q*_*R*_. These action values are used to determine choice through a softmax function with inverse temperature *β* that determines the choice probabilities,

(Equation 5) $$\text{P}(‘\text{Right}’)=\frac{1}{1+e^{-\beta \left(Q_{\text{R}}-Q_{\text{L}}\right)}}\;\text{and}\;\text{P}(‘\text{Left}’)=1-\text{P}(‘\text{Right}’)$$

from which a choice is sampled on each trial.

For each simulation, the initialization of *W*^1^ was sampled from a Gaussian distribution centered on fit values of the initial constant input weight *μ*_*k*_ and stimulus input weights $\mu_{w_{0}}$ (identical for left/right) such that:

(Equation 6) $$W_{init\;}^{1}=\left[\begin{array}{ccc} k & 0 & 0\\ 0 & w_{0} & 0\\ 0 & 0 & w_{0} \end{array}\right]+10^{-4}N(0,1)$$

where *k* was sampled from *𝒩* (*μ*_*k*_ = 1, 0.1) and *w*_0_ was sampled from $N\left(\mu_{w_{0}}=0.05,0.05\right)$ for each tutor-executor simulation, and from *𝒩* (*μ*_*k*_ = 1, 0.01) and $N\left(\mu_{w_{0}}=0.05,0.001\right)$ for each single-loss simulation. The values of *k* and *w*_0_ were re-sampled if not strictly positive, and all weights were reset to their absolute value and the off-diagonal terms to 10^−5^ after each update step. This resetting was also applied when computing the losses on each step. When investigating the effect of the nonnegativity and connectivity constraints in Figure S10, this resetting was either not applied or only the constant-pathway off-diagonal terms were reset to 10^−5^ for the fully-connected stimulus-pathway model.

Similarly, the initialization of *W*^2^ was sampled from a Gaussian distribution centered on fit values of the constant pathway weights $\mu_{C_{0}}$ (Figure 5A, aqua – all identical) and stimulus pathway weights $\mu_{s_{0}}$ (Figure 5A, pink – all identical),

(Equation 7) $$W_{init\;}^{2}=\left[\begin{array}{l} \begin{array}{ccc} c_{0} & s_{0} & s_{0} \end{array}\\ \;\begin{array}{ccc} c_{0} & s_{0} & s_{0} \end{array} \end{array}\right]+10^{-4}N(0,1)$$

where *c*_0_ was set to $\mu_{c_{0}}=0$ and *s*_0_ was sampled from $N\left(\mu_{s_{0}}=0.2,0.05\right)$ for each tutor-executor simulation, and to $\mu_{c_{0}}=0$ and sampled from $N\left(\mu_{s_{0}}=0.7,0.01\right)$ for each single-loss simulation. These values were also re-sampled if not strictly positive. The *W*^2^ weights were similarly reset to their absolute value when computing the loss and after each update step to enforce the nonnegativity constraint.

The softmax inverse temperature parameter *β* and learning rate *α* were also sampled from a gaussian centered on a value fit to the experimental data. More specifically, *β* was sampled from *𝒩*(*μ*_*β*_ = 9, 1) and *α* from *𝒩*(*μ*_*α*_ = 0.0026, 0.001) for each tutor-executor simulation and from *𝒩*(*μ*_*β*_ = 13, 0.01) and *𝒩*(*μ*_*α*_ = 0.0015, 0.001) for each single-loss simulation. The fitting procedure is described in the corresponding subsection. Simulations were run for 10,000 trials and those that reached 70% accuracy in less than 8,500 trials (approximately highest number of trials required for mice to learn) were included in our analyses.

#### Tutor-executor gradient descent learning rule

We refer to the model presented in Figure 5 as the ‘tutor-executor’ model due to its learning rule, which uses different reward prediction errors (RPEs) to train the weights in *W*^1^ and *W*^2^. The updates minimize three different losses through stochastic gradient descent (SGD): the ‘cortical’ loss for the weights in *W*^1^, the ‘stimulus cortico-striatal’ loss for the weights in the stimulus pathway of *W*^2^ (Figure 5A, pink), and the ‘constant cortico-striatal’ loss for the weights in the constant pathway of *W*^2^ (Figure 5A, aqua). Each of these losses is a RPE^2^ comparing predictions based on different subsets of inputs against trial outcome,

(Equation 8) $$‘\text{Cortical}’\;\text{loss}\;:\;{ℒ}^{\text{tot}\;}=\frac{1}{2}\delta^{{\text{tot}}^{\text{2}}}=\frac{1}{2}{\left(\text{Rew}-{\text{Q}}_{\text{ch}}\right)}^{2},$$

(Equation 9) $$‘\text{Stimulus}\;\text{cortico-striatal}’\;\text{loss}\;:\;{ℒ}^{\text{stim}\;}=\frac{1}{2}\delta^{{\text{stim}\;}^{2}}=\frac{1}{2}{\left({\text{Rew}-\text{Q}}_{\text{ch}}^{\text{stim}}\right)}^{2},$$

(Equation 10) $$‘\text{Constant}\;\text{cortico-striatal}’\;\text{loss}\;:\;{ℒ}^{\text{const}\;}=\frac{1}{2}\delta^{{\text{const}\;}^{2}}=\frac{1}{2}{\left({\text{Rew}-\text{Q}}_{\text{ch}}^{\text{const}}\right)}^{2},$$

where *Rew* is a binary variable indicating whether the trial was rewarded or not, and the subscript *ch* indicates the choice made on each trial (left/right). Here *Q*_*ch*_ is the ‘total’ *Q*-value that uses all inputs to form its predictions, while $Q_{ch}^{stim\;}$ and $Q_{ch}^{const\;}$ are ‘partial’ *Q*-values based on the stimulus and constant inputs:

(Equation 11) $$Q_{ch}=W_{0,0}^{1}W_{ch,0}^{2}+VSL\;W_{1,1}^{1}W_{ch,1}^{2}+VSR\;W_{2,2}^{1}W_{ch,2}^{2},$$

(Equation 12) $$Q_{ch}^{stim}=VSL\;W_{1,1}^{1}W_{ch,1}^{2}+VSR\;W_{2,2}^{1}W_{ch,2}^{2}$$

(Equation 13) $$Q_{ch}^{const\;}=W_{0,0}^{1}W_{ch,0}^{2},$$

where *VSL* and *VSR* are the binary inputs indicating the presence or absence of the right and left stimulus respectively and *ch* used as a subscript for the weights is 0 for left and 1 for right choices.

Gradient descent on these losses yields updates which depend on the trial outcome and choice,

(Equation 14) $$\text{Δ}W^{1}=-\alpha \frac{\partial {ℒ}^{tot}}{\partial W^{1}}=\alpha \left[\begin{array}{ccc} W_{ch,0}^{2} & 0 & 0\\ 0 & VSL\;W_{ch,1}^{2} & 0\\ 0 & 0 & VSR\;W_{ch,2}^{2} \end{array}\right]\delta^{tot},$$

(Equation 15) $$\text{Δ}W^{2,stim}=-\alpha \frac{\partial {ℒ}^{stim\;}}{\partial W^{2}}=\{\begin{array}{l} \alpha \left[\begin{array}{ccc} 0 & VSL\;W_{1,1}^{1} & VSR\;W_{2,2}^{1}\\ 0 & 0 & 0 \end{array}\right]\delta^{stim\;},\;if\;ch=L,\\ \alpha \left[\begin{array}{ccc} 0 & 0 & 0\\ 0 & VSL\;W_{1,1}^{1} & VSR\;W_{2,2}^{1} \end{array}\right]\delta^{stim\;},\;if\;ch=R, \end{array}$$

(Equation 16) $$\text{Δ}W^{2,const}=-\alpha \frac{\partial {ℒ}^{const\;}}{\partial W^{2}}=\{\begin{array}{l} \alpha \left[\begin{array}{ccc} W_{0,0}^{1} & 0 & 0\\ 0 & 0 & 0 \end{array}\right]\delta^{const\;},\;if\;ch=L,\\ \alpha \left[\begin{array}{ccc} 0 & 0 & 0\\ W_{0,0}^{1} & 0 & 0 \end{array}\right]\delta^{const\;},\;if\;ch=R, \end{array}$$

where *α* is the learning rate and Δ*W*^2^ = Δ*W*^2,*stim*^ + Δ*W*^2,*const*^. Notice how the updates for *W*^1^ are proportional to the total RPE, *δ*^*tot*^; the updates for the stimulus pathway in *W*^2^ are proportional to the stimulus-based RPE, *δ*^*stim*^; and lastly, the updates for *W*^2^’s constant pathway are proportional to the constant-based RPE, *δ*^*const*^. Interestingly, the general tendency of the learning rule is to minimize the total ‘cortical’ loss, L^*tot*^, as the learning in *W*^1^ *tutors* downstream learning in *W*^2^ by determining the relative salience of the inputs and balancing updates in the *executor* pathways. Shallow versions of this learning rule set the *W*^1^ updates to 0.

#### Single-loss gradient descent learning rule

This learning rule updates *W*^1^ and *W*^2^ through stochastic gradient descent (SGD) to minimize a single total RPE^2^. This corresponds to the conventional method of training deep RL networks, where all parameters are updated to minimize a single loss and thus share the same objective.^74^ The loss we use for this learning rule is the same as the ‘cortical’ loss in the tutor-executor network,

(Equation 17) $${ℒ}^{tot}=\frac{1}{2}\delta^{tot^{2}}=\frac{1}{2}{\left(\;Rew\;-Q_{ch}\right)}^{2},$$

>where *Rew* is a binary variable indicating the outcome of a particular trial and *Q*_*ch*_ is a reward prediction calculated based on all inputs to the network. The updates for *W*^1^ and *W*^2^ can be written as follows

(Equation 18) $$\text{Δ}W^{1}=-\alpha \frac{\partial {ℒ}^{tot}}{\partial W^{1}}=\alpha \left[\begin{array}{ccc} W_{ch,0}^{2} & 0 & 0\\ 0 & VSL\;W_{ch,1}^{2} & 0\\ 0 & 0 & VSR\;W_{ch,2}^{2} \end{array}\right]\delta^{tot}$$

(Equation 19) $$\text{Δ}W^{2}=-\alpha \frac{\partial {ℒ}^{tot\;}}{\partial W^{2}}=\{\begin{array}{l} \alpha \left[\begin{array}{ccc} W_{0,0}^{1} & VSL\;W_{1,1}^{1} & VSR\;W_{2,2}^{1}\\ 0 & 0 & 0 \end{array}\right]\delta^{tot\;},\;if\;ch=L,\\ \alpha \left[\begin{array}{ccc} 0 & 0 & 0\\ W_{0,0}^{1} & VSL\;W_{1,1}^{1} & VSR\;W_{2,2}^{1} \end{array}\right]\delta^{tot\;},\;if\;ch=R, \end{array}$$

where *α* is the learning rate and *VSL* and *VSR* are binary variables indicating the presence or absence of the right and left stimulus respectively. Here, all the updates are proportional to the same total RPE, *δ*^tot^. Shallow versions of this learning rule set the *W*^1^ updates to 0.

#### Model-derived dopamine signals

Our neural network model captures empirical dorsolateral striatal dopamine (DLS DA) signals through the weights in its stimulus pathway. Over learning, the model reproduces DLS DA outcome responses with a stimulus-based reward prediction error (RPE),

(Equation 20) $$\;\text{Outcome-time}\;\text{DLS}\;\text{DA}\;\equiv \delta^{stim\;}=Rew-Q_{ch}^{stim\;}$$

This differs from classic temporal difference reward prediction errors (TD-RPEs) commonly used in DA studies in that it does not use the full reward prediction based on all inputs to define the RPE, instead comparing trial outcome with a prediction based only on the stimulus inputs. This was motivated by our matched accuracy analysis in Figures 2F and 2G, which showed that DLS DA at both stimulus and outcome time does not reflect reward predictions based on the constant input (i.e., the absence of a stimulus).

At stimulus time, in accordance with the TD-RPE hypothesis,^31^ the model captured DLS DA signals with a pre-choice reward prediction. Importantly, this prediction is also based only on the stimulus inputs,

(Equation 21) $$\;\text{Stimulus-time}\;\text{DLS}\;\text{DA}\equiv p_{L}^{stim\;}Q_{L}^{stim\;}+p_{R}^{stim\;}Q_{R}^{stim\;}$$

where $p_{L}^{stim\;}$ and $p_{R}^{stim\;}$ are the left and right choice probabilities determined by the stimulus-based *Q*-values passed through the choice function in Equation 5. Here, there is no need to subtract the value of the previous state as there is no cue before the stimulus that is predictive of reward.

To test whether the network could be trained with a learning signal analogous to our DLS DA recordings, the tutor-executor network uses a learning rule based on these ‘partial’ prediction errors to update its weights. Specifically, the updates of the *W*^2^ stimulus pathway weights are proportional to the stimulus-based RPE, *δ*^*stim*^ (Equation 15). Comparing the evolution of *δ*^*stim*^ with the DLS DA outcome response over learning shows a striking similarity (Figures 5P, S8F, and S8I–S8K), suggesting that a similar dopamine-based learning mechanism could be governing the learning process of the mice.

#### Simulation of the optogenetic experiment

We simulated the optogenetic experiment in the model to investigate the predicted effect on behavior of the tutor-executor and single-loss gradient descent learning rules undergoing similar manipulations. For the tutor-executor learning rule, the effect of DLS DA stimulation was modelled as a ‘reward’ signal provided only to the ‘stimulus cortico-striatal’ loss ℒ^*stim*^. However, for the single-loss learning rule the ‘reward’ was provided to the total loss ℒ^*tot*^ used to update weights in *W*^2^. As for mice, simulated DLS DA stimulation was provided to expert one-sided simulations for incorrect left or right choices on alternating days (i.e. 220 simulated trials) for 10 days (i.e. 5 of each condition). The ‘reward’ signal used to model the stimulation was x0.25 the size of the reward provided by water, and a learning rate boost of x10^3^ on incorrect trials was used to reproduce the empirical results. Networks were initialized using weights from right-associating average dynamics at expertise.

We also simulated the incorrect trial water delivery control experiment. As in correct trials, the effect of water delivery on incorrect trials was simulated as a global reward signal provided to all the losses used for weight updates in both the tutor-executor and single-loss networks. As for mice, incorrect trial water delivery was provided to expert one-sided simulations for incorrect left or right choices on alternating days, with a baseline day in between where simulations performed the original task. The alternation was performed for 20 days (i.e. 5 days of each left/right condition) for each simulation. A learning rate boost of x5 on incorrect trials with water delivery was used to capture the empirical results. Networks were initialized using weights from right-associating average dynamics at expertise.

#### Average dynamics

Deriving the average dynamics of the model allowed us to obtain an analytical description of its learning mechanism. We took the continuous time limit of the average gradient descent updates for *W*^1^ and *W*^2^ from the tutor-executor and single-loss learning rules, averaging over trial type (left stim, right stim, or zero-contrast) and choice (left or right). This yielded a 9-dimensional system of coupled differential equations describing the evolution of each weight in the network. For sufficiently small learning rates *α*, this ‘gradient flow’ limit provides a good description of the average dynamics for both learning rules. The resulting differential equations were numerically integrated to obtain average weight trajectories over training time. To capture the three main types of learning trajectory (i.e., left-associating, balanced and right-associating), we initialized the integration with a network configuration yielding different degrees of initial choice bias (i.e., imbalanced connections from const. to *Q*_*L*_ and *Q*_*R*_). We then overlayed the resulting trajectories (thick dashed lines in model figures) on those from trial-by-trial simulations.

The average dynamics of the network weights for the tutor-executor learning rule are governed by three main differential equations; one for each of the cortical, stimulus cortico-striatal and constant cortico-striatal weight subsets (black, pink, and aqua in Figure 5A). These can be derived by taking the average over trial-types and choices of the gradient descent updates in Equations 14, 15, and 16. Doing this for the cortical weights in *W*^1^ we obtain

(Equation 22) $$\begin{array}{l} \frac{1}{\alpha }\frac{d}{dt}W^{1}={\langle {\langle -\frac{\partial {ℒ}^{tot\;}}{\partial W^{1}}\rangle }_{ch\;}\rangle }_{trial\;}=-{\langle {\langle \delta^{tot\;}\frac{\partial \delta^{tot\;}}{\partial W^{1}}\rangle }_{ch\;}\rangle }_{trial\;}=-p_{\text{VSL}\;}(p_{\text{L},\;\text{VSL}\;}\left(1-Q_{\text{L,}\;\text{VSL}\;}\right)\frac{\partial \left(1-Q_{\text{L,}\;\text{VSL}\;}\right)}{\partial W^{1}}\\ \;+p_{\text{R},\text{VSL}}\left(0-Q_{\text{R},\text{VSL}}\right)\frac{\partial \left(0-Q_{\text{R},\text{VSL}}\right)}{\partial W^{1}})-p_{0}(0.5p_{\text{L},0}\left(1-Q_{\text{L},0}\right)\frac{\partial \left(1-Q_{\text{L},0}\right)}{\partial W^{1}}+0.5p_{\text{L},0}\left(0-Q_{\text{L},0}\right)\frac{\partial \left(0-Q_{\text{L},0}\right)}{\partial W^{1}}\\ \;+0.5p_{\text{R},0}\left(1-Q_{\text{R},0}\right)\frac{\partial \left(1-Q_{\text{R},0}\right)}{\partial W^{1}}+0.5p_{\text{R},0}\left(0-Q_{\text{R},0}\right)\frac{\partial \left(0-Q_{\text{R},0}\right)}{\partial W^{1}})-p_{\text{VSR}}(p_{\text{L},\text{VSR}}\left(0-Q_{\text{L},\text{VSR}}\right)\frac{\partial \left(0-Q_{\text{L},\text{VSR}}\right)}{\partial W^{1}}\\ \;+p_{\text{R,VSR}}\left(1-Q_{\text{R},\text{VSR}}\right)\frac{\partial \left(1-Q_{\text{R,VSR}}\right)}{\partial W^{1}}) \end{array}$$

where *t* is a continuous time variable counting the number of trials; *δ*^*tot*^ represents the total RPE; *p*_VSL_ = *p*_VSR_ = 0.45 and *p*_0_ = 0.1 are the probabilities of there being a left vis. stim., right vis. stim., and zero-contrast trial; and lastly *p*_A,B_ and *Q*_A,B_ indicate the choice probabilities and total *Q*-values for choice A in a trial of type B. The choice probabilities are calculated using the sigmoidal choice rule in Equation 5. The partial derivatives of the *Q*-values can then be expanded by writing them in terms of the network weights (Equations 11, 12, and 13) and differentiating w.r.t. the *W*^1^ matrix.

The same procedure can be followed to find the differential equations for the weights in the stimulus and constant pathways of *W*^2^, with gradient flow equations

(Equation 23) $$\frac{1}{\alpha }\frac{d}{dt}W^{2,\;stim\;}={\langle {\langle -\frac{\partial {ℒ}^{stim\;}}{\partial W^{2,\;stim\;}}\rangle }_{ch}\rangle }_{trial\;}=-{\langle {\langle \delta^{\text{stim}\;}\frac{\partial \delta^{\text{stim}\;}}{\partial W^{2,\;stim\;}}\rangle }_{ch}\rangle }_{trial\;}$$

(Equation 24) $$\frac{1}{\alpha }\frac{d}{dt}W^{2,const}={\langle {\langle -\frac{\partial {ℒ}^{const}}{\partial W^{2,const}}\rangle }_{ch}\rangle }_{trial}=-{\langle {\langle \delta^{const}\frac{\partial \delta^{const}}{\partial W^{2,const}}\rangle }_{ch}\rangle }_{trial}$$

The single-loss average dynamics can be derived in a similar fashion, where now all the weights are minimizing the same loss function (Equation 17), yielding the following gradient flow equations

(Equation 25) $$\frac{1}{\alpha }\frac{d}{dt}W^{1}={\langle {\langle -\frac{\partial {ℒ}^{tot\;}}{\partial W^{1}}\rangle }_{ch\;}\rangle }_{trial\;}=-{\langle {\langle \delta^{\text{tot}\;}\frac{\partial \delta^{\text{tot}\;}}{\partial W^{1}}\rangle }_{ch\;}\rangle }_{trial\;}$$

(Equation 26) $$\frac{1}{\alpha }\frac{d}{dt}W^{2}={\langle {\langle -\frac{\partial {ℒ}^{tot\;}}{\partial W^{2}}\rangle }_{ch}\rangle }_{trial\;}=-{\langle {\langle \delta^{\text{tot}\;}\frac{\partial \delta^{\text{tot}\;}}{\partial W^{2}}\rangle }_{ch}\rangle }_{trial\;}$$

which can be expanded as exemplified with the tutor-executor dynamics.

#### Saddle Points

Saddle points in the model’s learning dynamics provide an explanation for the systematic transitions between behavioral strategies and dopamine release patterns observed in the mice. We used the average dynamics derived in the sections above to demonstrate the existence of these saddle points. We first made informed guesses at stationary points by looking for network configurations where the average dynamics go to 0, and then investigated the dynamics around these points to verify their nature. These derivations can be found in the Mathematica notebooks tutor_executor_fixed_points.nb and single_loss_fixed_points.nb from the paper’s associated code repository.

We also provide evidence for heteroclinic orbits connecting the saddle points, represented by the arrows in Figures 7A and S9B. To do this, we used the string method^75^ to find the minimal energy path between each pair of saddle points. This allowed us to distinguish between points that are directly connected by such paths (i.e., heteroclinic orbits), and points that are only connected through another one of the saddle points. The orbits discovered by the string method are shown in the Mathematica notebooks, and their schematic form is shown in Figures 7A and S9B.

#### Fitting procedure

To reproduce the learning trajectories in the data with our model, we fit the mean value of the network weights at initialization (see Equations 6 and 7) and the *β* parameter of the choice function (Equation 5). Specifically, we fit the initial mean *W*^1^ weights for the constant *μ*_*k*_ and stimulus inputs $\mu_{W_{0}}$ (equal for left/right), the initial mean *W*^2^ weights for the constant $\mu_{C_{0}}$ and stimulus pathways $\mu_{s_{0}}$ (all equal within each pathway) and the mean value of the inverse temperature *μ*_*β*_.

To do so, we took advantage of our expressions for the average dynamics of both the tutor-executor and single-loss learning rules. We integrated the average dynamics of networks initialized with different values of the mean initial weights, of the inverse temperature *μ*_*β*_ and with a small amount of left, right or no initial bias *ε* in the constant-pathway *W*^2^ weights (i.e. $W_{1,0}^{2}=W_{0,0}^{2}+\varepsilon$, *ε* was also fit when non-zero) until 73% accuracy. We then compared the resulting behavioral and neural trajectories with the left-assoc., right-assoc. and balanced trajectory clusters from the data (from Figures 1J–1M and 2D).

The fitting procedure minimized the mean squared error between each trajectory measure for the three behavioral and neural clusters emerging from integrating the average dynamics and the three trajectory clusters from the data using momentum-based gradient descent on the parameters. The optimization was performed using a chained optimizer from the JAX-based optax module with optax. clip(1), optax.adabelief(learning_rate=1e-3) and optax.keep_params_nonnegative(). We started the optimizer with 5 different initial parameter settings sampled randomly from a suitable range for each parameter. We then ran the optimization for 1000 iterations, finally selecting the parameter combination with the lowest mean squared error. This method using the average dynamics proved faster than with simulations. Lastly, the learning rate *α* was fine-tuned by hand to obtain learning trajectories that were stable and learned in a similar number of trials as the mice.

The resulting parameter values were used as the means of Gaussians (with variances set manually to reproduce similar patterns of diversity as observed in mice) from which the parameters of each simulated network were sampled for the simulations shown in the figures across the paper (see Equations 6 and 7). Further, the resulting parameters (including *ε*) were also used as initialization for the trajectories derived from integrating the average dynamics shown as dashed thick lines across the paper.

#### Generalization to larger networks

To investigate whether the learning principles we identified in small networks generalize to larger, more conventional neural networks, we trained such networks on our behavioral task (Figure S10). First, we improved the faithfulness of the model to the mice’s perceived environment by replacing the one-hot encoding of the stimulus inputs with a pixel-level representation of the screen presented to the mice. We used a downsampled version of the 1280x1024 gray screen with a Gabor stimulus presented at an x-offset of +/-423 pixels from the center with a frequency of 0.01348 cycles/pixel and a Gaussian envelope with variance 98.69 pixels^2^ in both the x- and y-directions. Second, we increased the number of neurons in each layer and the number of layers while maintaining the connectivity pattern of the smaller network (i.e., one-to-one input channels, fully-connected stimulus pathways or a completely fully-connected network). Third, we introduced a non-linear activation function (i.e. ReLU) to further improve the faithfulness of the model to brain networks. The output of the large networks remained as the same as the small network: two Q-values represented by single neurons, which were subsequently fed to a softmax function to determine the probability of each choice. A complete depiction of the large network architectures can be found in Figures S10G–S10I. The networks were trained with the single-loss gradient descent learning rule, i.e., minimizing the total loss *ℒ*^*tot*^, as implementing the tutor-executor learning rule in deeper multi-layer networks is outside the scope of this study. We kept the nonnegativity constraint as we found it to work best for the smaller network.

For each simulation, the large networks were initialized with a similar effective network to the smaller one-to-one network (set to the fit values of *W*^1^ and *W*^2^ from before). More specifically, the weights in the first and last layers were sampled from Gaussians with variances set to the fit values from the small network (negative weights clipped to 0), and weights in intermediate layers were initialized small. For fully-connected networks, the cross-connections were also initialized small (except where we explicitly investigated manipulating their magnitude). The softmax inverse temperature parameter *β* and learning rate *α* were also sampled from a gaussian centered on the value fit to the experimental data for the smaller network. The fitting procedure is described in the corresponding subsection. Simulations were run for 10,000 trials and those that reached 70% accuracy in less than 8,500 trials (approximately highest number of trials required for mice to learn) were included in our analyses.

#### Single-state Q-value model

To investigate the origin of the diverse early biases across mice, we fit a simple State-Action-Reward-State-Action (SARSA) model to mouse choices in the first 5 days of training (Figure S1Q). The model had two *Q*-values for the left and right choices (*Q*_L_ and *Q*_R_) independent of trial type (i.e., with a single state). Each *Q*-value was defined as the sum of an innate (fixed per mouse) choice tendency for each of the choices (*Q*^innate^), a choice tendency that changes day-by-day (*Q*^day^, one for each day), and a choice tendency that is shaped by reward history (*Q*^rew^):

(Equation 27) $$Q_{L/R}=Q_{L/R}^{innate\;}+Q_{L/R}^{day\;(n)}+Q_{L/R}^{rew\;}$$

where *n* indicates the day (from 1 to 5) corresponding to each trial. The choice probabilities on each trial were determined by passing the resulting left and right *Q*-values through a softmax function with inverse temperature parameter *β*, as for the paper’s main model. However, unlike the models above, the choice on each trial was taken from the mice’s choice history. Based on this choice, *Q*^rew^ was updated on each trial using the regular SARSA update with two learning rates α^+/-^:

(Equation 28) $$\text{Δ}Q_{ch}^{rew\;}=\alpha^{+/-}\left(Rew.\;-Q_{ch}^{rew\;}\right)$$

where the *Q*-value corresponding to the choice (*Q*_ch_) was updated using α^+^ and a reward signal *Rew.=1* on trials where the mice were rewarded, and α^-^ with *Rew.=0* for when they were not.

We performed a nested model comparison by removing different combinations of the *Q*-value components (innate, day and rew) and fitting each model variation to each mouse’s choice and reward history data. The values of *Q*^innate^, *Q*^day(1-5)^, *β* and α^+/-^ were fit per mouse for each version of the model by simulating the *Q*-value trajectories for each parameter setting, and minimizing the resulting log-likelihood of the mouse’s choices (based on the model-derived choice probabilities on each trial) with momentum-based projected gradient descent (optax.adam, run for 5000 iterations with learning rate 0.001 and parameters clipped to 0 on each update step if they go negative). Finally, the Bayesian Information Criterion (BIC) for each model variation fit to each mouse’s data was computed.

### Software packages

All data analyses were performed using custom code written in Python 3 using standard analysis and plotting libraries: numpy, scipy, scikit-learn, matplotlib and seaborn. For the model, the JIT compilation and automatic differentiation capabilities of JAX were used to accelerate and simplify gradient calculations. Fitting was performed using the JAX-based optax module. PyTorch was used to define and train the large networks. A modified version of Rigbox (CortexLab) running on MATLAB 2019a was used for recording behavioral data and Bonsai was used for neural recordings and optogenetic stimulation. Fiber tracts were estimated from histology using the allenCCF toolbox (CortexLab) for MATLAB.

### Statistics

The sample sizes are higher than sample sizes typically used in the field, due to the study’s emphasis on individual variability. No statistical methods were used to determine sample size. Trial types (i.e., stimulus side and contrast level) were randomly determined by a computer program during behavior. Details of all statistical analyses are provided in the figure legends. Analyses were performed using custom code written in Python 3 using standard analysis libraries: numpy, scipy, statsmodels. The statistical parameters of each analysis are reported in figure captions.

## Supplementary Material

Supplementary Material

## Data Availability

Behavioral, neuronal, and modeling data have been deposited at Figshare and are publicly available as of the date of publication at https://doi.org/10.6084/m9.figshare.28877912. All original code has been deposited at Figshare and is publicly available at https://doi.org/10.6084/m9.figshare.28877942 as of the date of publication. Any additional information required to reanalyze the data reported in this paper is available from the lead contact upon request.

## Highlights

- Individuals form diverse yet systematic learning trajectories from naive to expert
- Dorsal striatal dopamine acts as a teaching signal shaping the trajectories
- Deep but not shallow RL with heterogeneous teaching signals accounts for mice data
- A mathematical framework explains diversity and systematicity of long-term learning
