# Supplementary material for: Dopamine encodes deep network teaching signals for individual learning trajectories
Source: Cell. Author manuscript; Available in PMC 2026 Aug 11. (PMC7619352; doi:10.1016/j.cell.2025.05.025)
Supplement: Supplementary file 1 [file EMS217539-supplement-Supplementary_Material.pdf]

CellPress
OPEN ACCESS

Supplemental figures

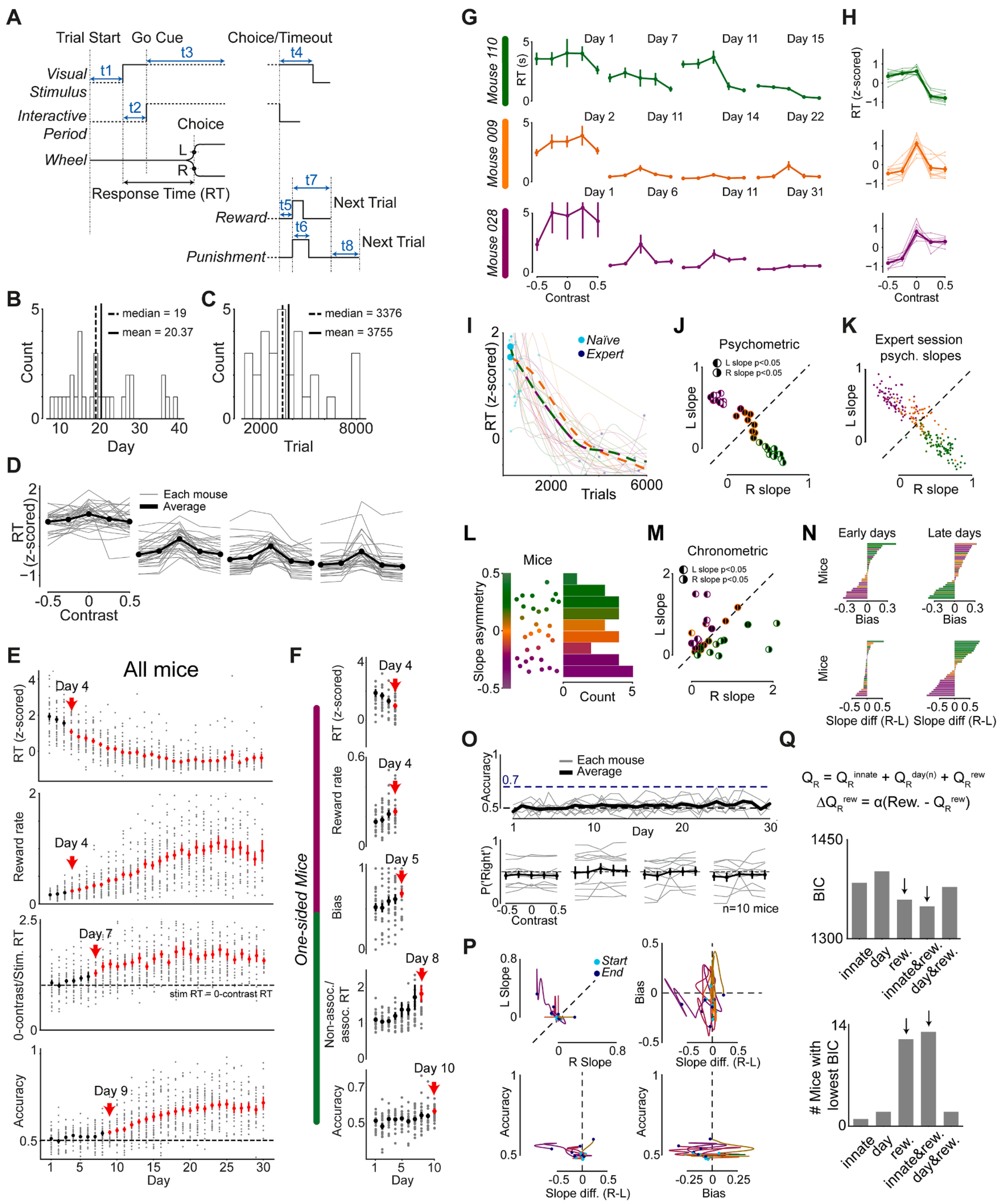

Figure S1. Learning a visual decision task from naive to expert, related to Figure 1

(A) The temporal structure of the task within a trial. Words in regular font indicate trial events, words in italics are labels for the traces in the timing diagram, and the solid vs. dashed style of the traces indicate fixed vs. variable time periods respectively. The duration of each labeled time period is provided in the STAR Methods.

(B) Histogram of the number of days that mice required to reach 70% accuracy.

(C) Histogram of the number of trials that mice required to reach 70% accuracy.

(legend continued on next page)

(D) Chronometric curve over quartiles per mouse (gray) and averaged across all mice (black). RT (*Z* scored) indicates the mean *Z* scored RT (time from stimulus onset to choice completion) per contrast value. For each mouse, the *Z* score was computed by first finding the mean RT per contrast across trials in each day, these values were subsequently standardized across days and contrast values and finally averaged across days in each quartile. Error bars indicate ± SEM across mice in all panels, unless specified differently.

(E) Measures of general RTs (averaged across trials independent of stimulus contrast and side), reward rate, the ratio of RTs on zero-contrast trials and trials with stimuli, and accuracy over the first 30 days of learning. First plot, *Z* scored RTs (across all trial types) over training days per mouse (gray) and averaged across all mice (black/red). Red dots indicate data points significantly different from day 1 averages ($p < 0.05$, estimated via two-sided t test), and the red arrow indicates the first day this occurs. *Z* scoring was performed by standardizing each mouse's mean RT per day using data from all its days. Second plot, reward rate over training days, calculated as the choice accuracy divided by the mean (not *Z* scored) RT for each day. Influenced by the decrease in RTs, the reward rate also became significantly different from the first day on day 4. Third plot, the ratio of the average (non-*Z* scored) RTs measured in zero-contrast trials to the average RTs in left and right stimulus trials. On day 7, the RTs were significantly faster in trials with stimuli, compared with zero-contrast trials. Fourth plot, accuracy over training days. The accuracy only significantly surpasses chance level on day 9. For all measures, only days with more than 90 trials were considered in the analysis.

(F) Similar analysis to (E), visualized selectively for one-sided mice over the first days of learning until significance for each variable was reached. A measure of the average zero-contrast bias over days was also included. Further, the RT ratio was changed to plot the ratio of RTs on trials with non-associated stimuli (including zero-contrast) vs. associated stimuli. This demonstrates that choice biases emerged before mice learned to associate stimuli with choices to make faster decisions.

(G) Chronometric curves for the same mice and days as in Figure 1D. Error bars indicate ± SEM across trials in each day.

(H) Expert day chronometric curves per mouse (thin) and averaged per trajectory cluster (thick). Cluster labels for each mouse were obtained from Figure 1J. The *Z* scored RT was defined as in (D).

(I) *Z* scored RT over trials per mouse (thin) and for the 3 clusters from Figure 1J with left- and right-associating mice combined into a single group (thick dashed). The *Z* scored RT was defined as in (E). Number of trials limited to 6,000.

(J) Scatter of average right vs. left psychometric slopes in expert days for each mouse. Stroke color indicates the average slope asymmetry of each mouse. The fill of the left/right half of each circle (black/white) indicates the statistical significance of the left/right slopes (i.e., whether the mean P(''Right'') for left/right stimulus trials is significantly different from that of zero-contrast trials, assessed with a Bernoulli statistic, black means significant at 95% i.e., Bernoulli statistic >1.96 or <−1.96).

(K) Scatterplot of expert day right vs. left psychometric slopes, colored by the average slope asymmetry of the mouse trajectory they belong to.

(L) Color scheme (left), scatter (middle), and histogram (right) of average psychometric slope asymmetry per mouse. Slope asymmetry was calculated as 0.5 times the weighted average of the difference in R-L slopes over all days in a mouse's learning trajectory. The weightings in the average are the sum of both R+L slopes per day.

(M) Scatter of average right vs. left chronometric slopes in expert days for each mouse. Chronometric slopes are defined as the difference between the median RT on zero-contrast trials and trials with right/left stimuli.

(N) Bar plots showing the average bias and slope difference in early (4–8) and late (final 5) days for each mouse, colored by average psychometric slope asymmetry.

(O) Top, accuracy over days per non-learner mouse (gray) and averaged across all non-learner mice (black). Dashed lines indicate chance level (black) and 70% accuracy level (blue). Number of days limited to 30. Bottom, psychometric curve over quartiles per mouse (gray) and averaged across all mice (black). In all panels, error bars indicate ± SEM across mice.

(P) In clockwise order from top left, right vs. left slope; difference in right and left (R-L) slope vs. bias; bias vs. accuracy; and R-L slope vs. accuracy across days per non-learner mouse. Trajectory colors obtained from the average slope asymmetry as in (L).

(Q) Results from a simple SARSA model assessing the effect of innate biases, day-by-day tendencies, and reward-driven promotion of each choice. The model was fit to choice data from the first 5 days of training. Left, the BIC of each model in a nested model comparison selectively removing different combinations of model parameters. Arrows indicate the two model variations with the lowest BIC. Right, for each model variation, a count of the number of mice whose choice data had the lowest BIC for that variation compared with others. Arrows indicate the two model variations with the largest number of mice.

CellPress
OPEN ACCESS

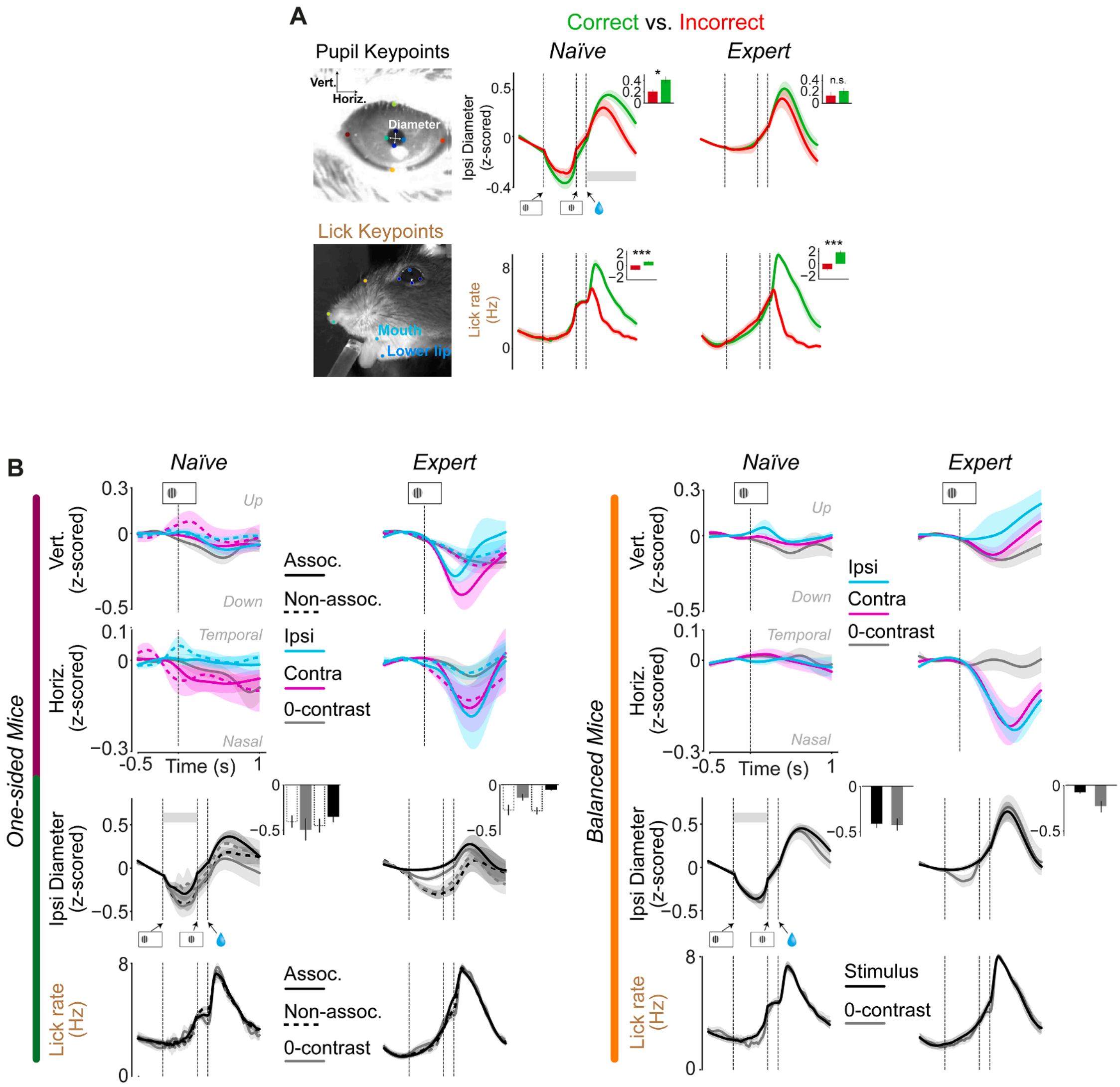

Figure S2. Eye movement and lick analyses, related to Figure 1

(A) Left, example video frames showing the key points used to analyze eye and lick data. Right, time warped measures of ipsilateral pupil diameter (top) and lick rate (bottom) on correct (green) vs. incorrect (red) trials of balanced mice where the previous trial was rewarded. Naive days are defined as the first 5 days of each mouse, and expert days have accuracy n.s. smaller than 0.7. Shaded bar shows the analysis window used for the bar plot insets. *p* values calculated using two-sided t test.

(B) Top, average vertical and horizontal eye movements (pupil motion) aligned to stimulus onset in one-sided and balanced mice for correct trials with stimuli ipsilateral (cyan) and contralateral (fuchsia) to the pupil, as well as on zero-contrast rewarded trials (gray). In one-sided mice, solid lines indicate pupil motion in response to "associated" stimuli and dashed lines to "non-associated" stimuli. Bottom, time warped measures of ipsilateral pupil diameter (top) and lick rate (bottom) for correct trials with associated (black solid) and non-associated (black dashed) stimuli, as well as rewarded zero-contrast trials where the associated (gray solid) and non-associated (gray dashed) choice was made. Only trials that directly follow a rewarded trial are plotted. For one-sided mice, naive days are defined as the first 3 days and for balanced mice as the first 5 days (like in A). Expert days have accuracy n.s. smaller than 0.7 for both groups. Shaded bar shows the analysis window used for the bar plot insets. n.s., $p > 0.05$, *$p < 0.05$, **$p < 0.005$, and ***$p < 0.0005$, respectively.

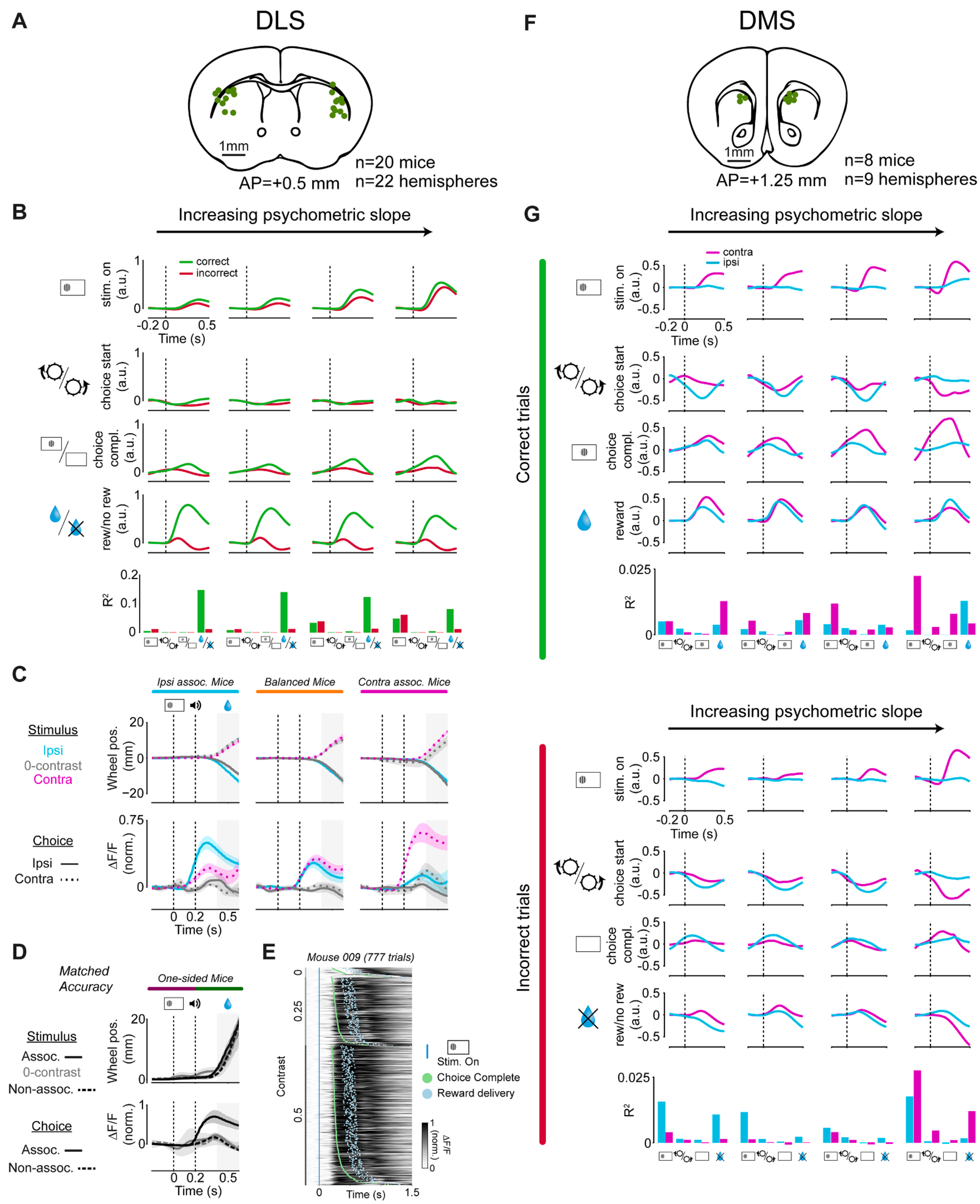

Figure S3. DA signals in DLS and DMS during task learning, related to Figure 2

(A) The locations of DLS optic fiber implants estimated from post-mortem histological examinations overlaid on a brain slice schematic.

(B) Top, regression coefficients from a linear deconvolution of DLS DA signals for different events in a trial: stimulus onset, choice start, choice completion, and outcome (reward/no reward). The deconvolution uses the time points around each event as categorical regressors and then finds the least squares regression coefficients predicting the original signal. 25% and 50% contrast trials were pooled together, as done for analyses throughout the paper. The data were divided into correct and incorrect trials and were grouped into four bins of increasing psychometric slope. The linear deconvolution was performed independently for each subset of data. The resulting deconvolved signals were baselined using the average signal in the 0.2 s before each event. Bottom, R^2 for each trial event used in the linear deconvolution, calculated independently for correct and incorrect trials and for each level of psychometric slope.

(C) Wheel position (positive for contralateral and negative for ipsilateral movements) and simultaneous DLS DA recordings aligned to stimulus onset in expert days (accuracy n.s. smaller than 0.7) of one-sided mice with fiber implants ipsilateral (left column) and contralateral (right column) to the associated stimulus, as well as balanced mice (middle column). Data are shown for correct trials with $0.4 < RT < 0.6$ s with stimuli ipsilateral (cyan) and contralateral (fuchsia) to the recording

(legend continued on next page)

CellPress
OPEN ACCESS

fiber, as well as rewarded zero-contrast trials. Trials were further split by whether the choice made was ipsilateral (solid) or contralateral (dashed) to the fiber. Shaded region indicates the time period in which rewards were delivered. In all panels, error bars indicate ± SEM across mice.

(D) Wheel position (mm, absolute value i.e., non-directional) and simultaneous DLS DA recordings aligned to stimulus onset in expert one-sided days with matched accuracy on trials with associated and non-associated stimuli. Data are shown for correct trials with $0.4 < RT < 0.6$ s with associated (black solid) and non-associated (black dashed) stimuli, as well as rewarded zero-contrast trials where the associated (gray solid) or non-associated (gray dashed) choice was made. As in (C), shaded region indicates the time period in which rewards were delivered.

(E) Trial-wise stimulus-aligned DLS DA recordings from 4 example expert days of an example balanced mouse (only correct trials with reward delivery <1.5 s after stimulus onset), grouped by the contrast of the stimulus presented on the trial (pooling left and right stimuli) and sorted by RT within each contrast group. Blue line indicates the time of stimulus onset, green and light blue dots indicate the time of choice completion and reward delivery respectively.

(F) The locations of DMS optic fiber implants estimated from post-mortem histological examinations overlaid on a brain slice schematic.

(G) Same linear deconvolution analysis as in (B), now applied to DMS DA signals across learning. For the DMS DA deconvolution analysis, trials were further subdivided into whether the stimulus was presented ipsilateral (cyan) or contralateral (fuchsia) to the recording fiber due to the pronounced effect of stimulus laterality on this signal.

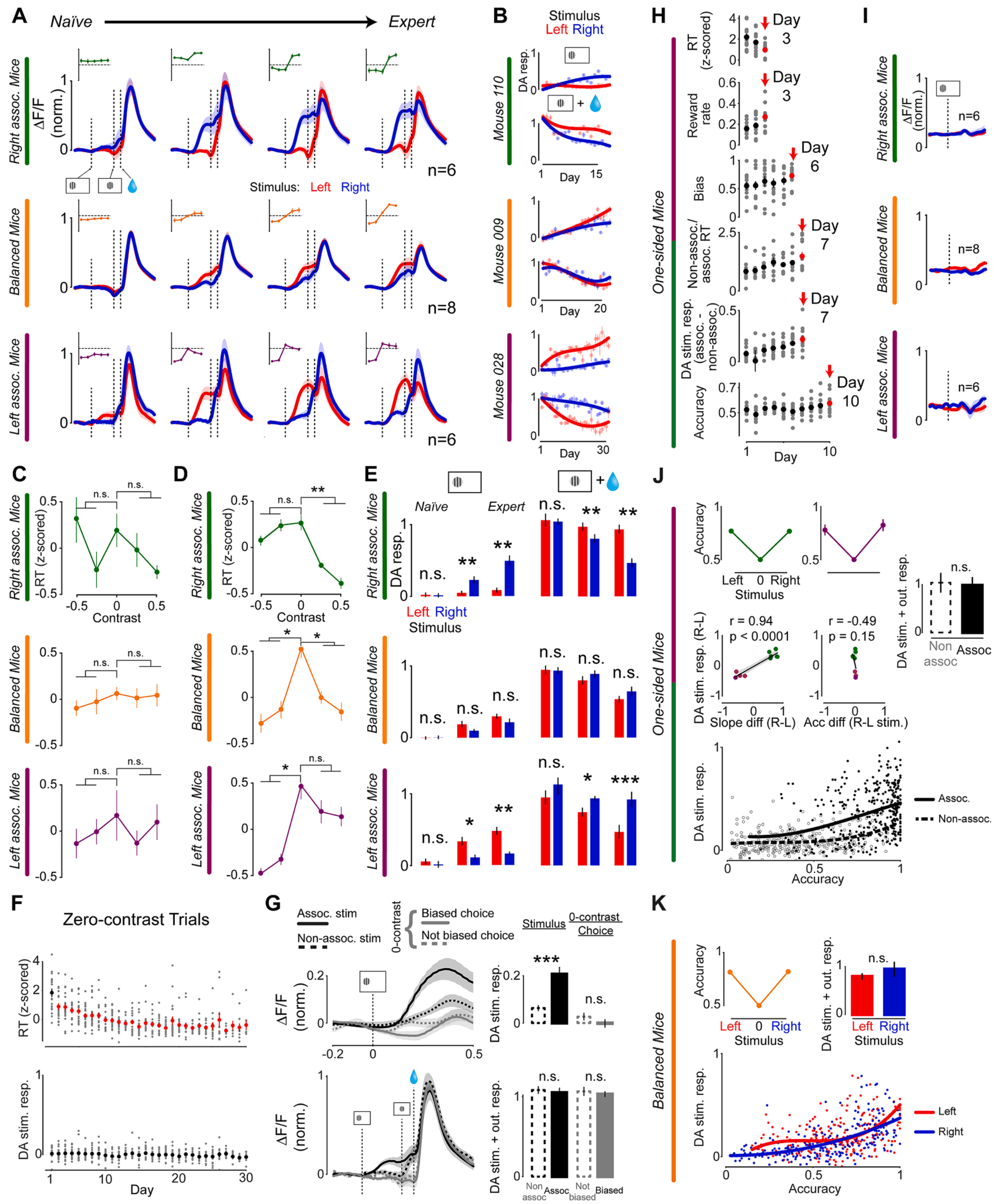

Figure S4. DLS DA signals during learning across mice, related to Figure 2

(A) Average time warped DLS DA signals across mice in each cluster, plotted over quartiles for correct trials with stimulus on the left (red) and right (blue), cf. Figure 2C. In order from left to right, vertical dashed lines indicate stimulus onset, stimulus-in-the-center, and reward delivery time. Insets show average psychometric curves across mice in each cluster for every quartile. Unless specified otherwise, error bars ± SEM across mice in all panels.

(B) Average stimulus and outcome DLS DA responses over days in correct trials for the three example mice from Figures 1D and 2C. Error bars indicate ± SEM across trials. Data points fit with a 3rd degree spline to visualize trend (scipy.interpolate.UnivariateSpline), cf. Figure 2D.

(C) Average chronometric curve across days 1–3 averaged across mice in each cluster. The *Z* score was computed per mouse by computing the mean RT per contrast across trials in each day, standardizing these values across contrast values and days, and finally averaging across days. *p* values calculated using two-sided paired t test.

(D) Average chronometric curve across days > 3 with accuracy n.s. greater than 0.5, averaged across mice in each cluster. *p* values calculated using two-sided paired t test.

(legend continued on next page)

CellPress
OPEN ACCESS

(E) Quantification of DLS DA signals from Figure 2E. Left, bar plots showing average DLS DA stimulus responses across mice in each cluster for correct trials in days 1–3, days >3 with accuracy n.s. greater than 0.5 and expert days (accuracy n.s. smaller than 0.7). p values calculated using two-sided paired t test. Right, same as left but for outcome responses.

(F) Top, average Z scored RTs in zero-contrast trials over first 30 days of training per mouse (gray) and averaged across all mice (black/red). Red dots indicate data points significantly different from day 1 averages ($p < 0.05$, estimated via two-sided t test). Z scoring was performed by standardizing each mouse's mean zero-contrast RT per day using data from all its days. Bottom, average DLS DA stimulus responses in zero-contrast trials (average 0.2–0.35 s after the stimulus would have been presented, i.e., 0.2 s before the auditory go cue) over days.

(G) Top left, average stimulus-aligned DLS DA signals in correct trials from days with accuracy n.s. greater than 0.5 of one-sided animals. Solid and dashed black lines show signals in trials with associated and non-associated stimuli respectively. Solid and dashed gray lines show signals on zero-contrast trials (aligned to the time when the stimulus would have been presented, i.e., 0.2 s before the auditory go cue) for choices in (solid) and opposite (dashed) to the direction of each mouse's early bias. Bottom left, average time warped DLS DA signals. Top right, quantification of average DLS DA stimulus responses. Bottom right, the sum of average DLS DA responses to stimulus and outcome. p values calculated using two-sided paired t test.

(H) Measures of general RTs, reward rate, zero-contrast bias, the ratio of RTs on trials without and with associated stimuli, the difference in the average DLS DA stimulus responses with and without associated stimuli and the accuracy over the initial days of learning for one-sided mice with DLS DA recordings. Data points are shown per mouse (gray) and averaged across all mice (black/red). Red dots indicate data points significantly different from day 1 averages ($p < 0.05$, estimated via two-sided t test). Data points for each measure are shown until significance was reached, indicated with a red arrow.

(I) Stimulus-aligned DLS DA signals in correct trials of the first day of training. No stimulus responses were significantly greater than 0 ($p > 0.05$; one-sample two-sided t test).

(J) Top, for one-sided mice in days with matched accuracy for trials with left and right stimulus, in clockwise order from top left, average accuracy across trial types; bar plots with sum of average DLS DA stimulus and outcome responses in correct trials; regression of difference in R-L psychometric slopes (left) and choice accuracy in R-L stimulus trials (right) against difference in DLS DA responses to R-L stimuli in correct trials. Each point in the regressions represents a matched accuracy day, and the p value is calculated from the exact distribution of r. Shaded region indicates 95% confidence interval. Bottom, for one-sided mice, scatterplot of average DLS DA stimulus responses against accuracy in associated (solid) and non-associated (dashed) stimulus trials. Each point represents a day. Data points fit with a 3rd degree spline to visualize trend (scipy.interpolate.UnivariateSpline).

(K) Similar analysis to that in (J) reproduced for balanced mice. n.s., $p > 0.05$, *$p < 0.05$, **$p < 0.005$, and ***$p < 0.0005$, respectively.

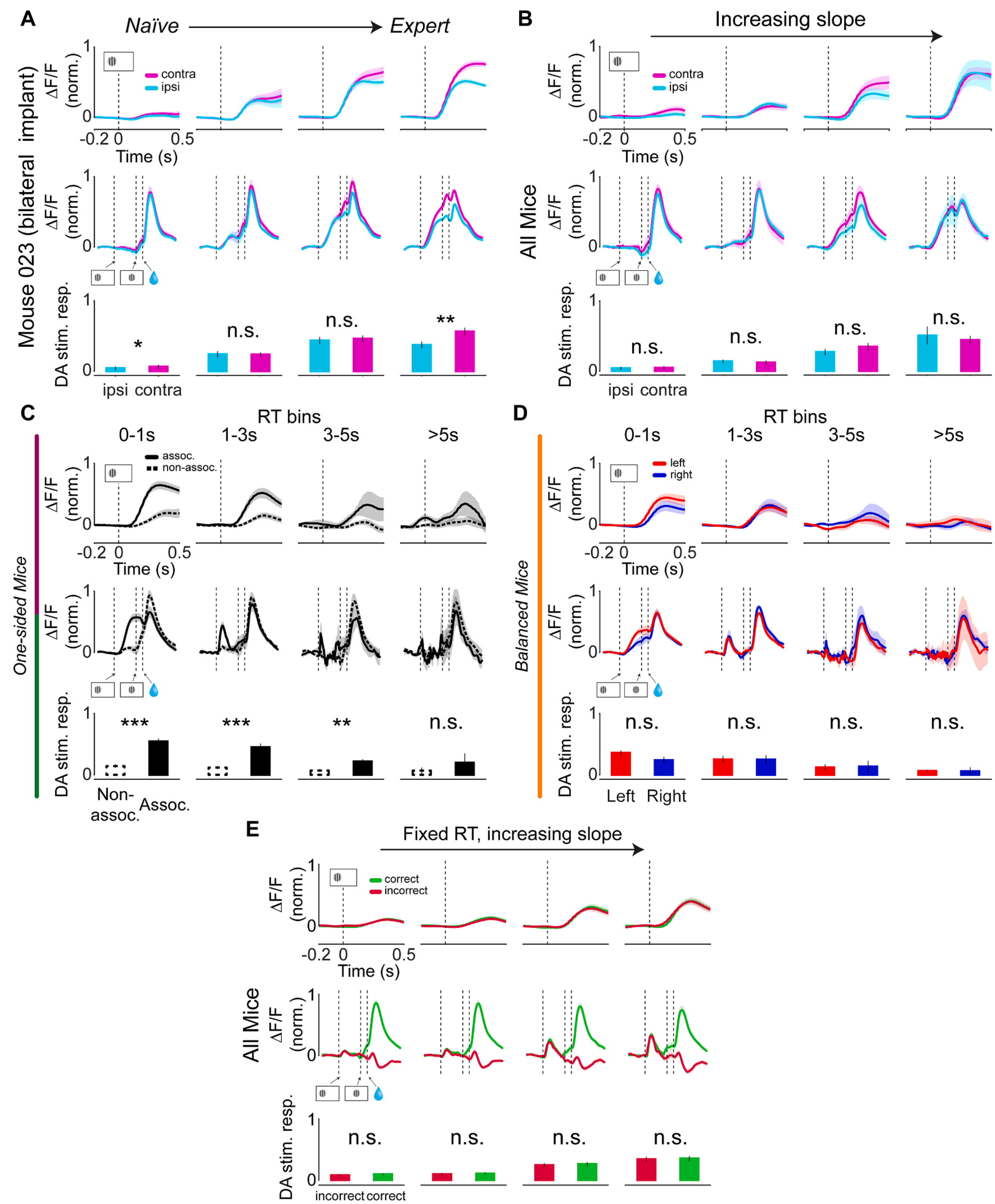

Figure S5. DA signals during learning, split by stimulus type, RTs, and trial outcome, related to Figure 2

(A) Average DLS DA stimulus-aligned signals (top), time warped signals (middle), and bar plots with average DLS DA stimulus responses (bottom) for rewarded trials from each quartile of an example mouse with bilateral implants, split by stimulus laterality with respect to the implanted fiber. Error bars indicate ± SEM across days. *p* value calculated from two-sided paired t test.

(B) Same as (A) for increasing psychometric slope values of all mice, also split by stimulus laterality with respect to the implanted fiber. In all panels, error bars indicate ± SEM across mice, unless specified differently.

(C) Average DLS DA stimulus-aligned signals (top), time warped signals (middle) and bar plots with average DLS DA stimulus responses (bottom) in rewarded trials for increasing RT values in expert days (accuracy > 0.7) of one-sided mice split by trials with the associated (solid) or non-associated stimulus (dashed). *p* value calculated from two-sided paired t test.

(legend continued on next page)

(D) Same as (C) for balanced mice.

(E) Average DLS DA stimulus-aligned signals (top), time warped signals (middle), and bar plots with average DLS DA stimulus responses (bottom) across all mice for increasing psychometric slope and a fixed RT range (1–3 s) split by correct and incorrect trials. n.s., $p > 0.05$, *$p < 0.05$, **$p < 0.005$, and ***$p < 0.0005$, respectively.

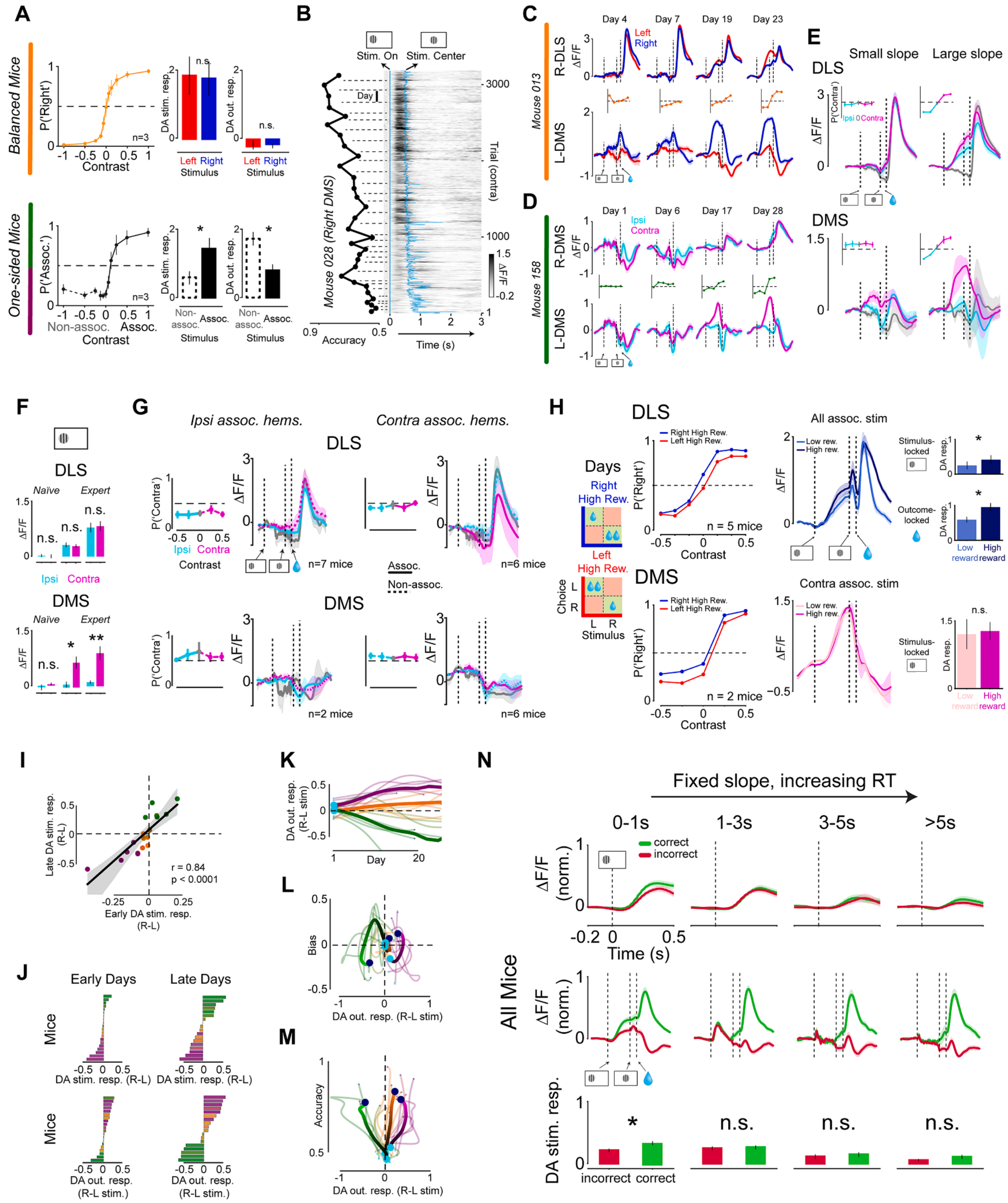

Figure S6. DA signals in highly expert mice and quantification of DLS and DMS DA responses to task events during learning, related to Figures 2 and 3

(A) Psychometric curves, average DLS DA stimulus responses and outcome responses in correct trials of highly expert (accuracy 80%–95%) balanced (top) and one-sided (bottom) mice trained with shaping, more contrast levels and more extensive training.

(B) Cf. Figure 2B, accuracy over days and simultaneous trial-wise stimulus-aligned DMS DA recordings from an example mouse (only correct trials with stimulus contralateral to the recording fiber and reward delivery <5 s after stimulus onset). Blue lines indicate the time of stimulus onset (left) and a 5-trial moving average of the time when the stimulus is brought to the center (i.e., choice completion, right).

(C) Average time warped DLS and DMS DA signals in correct trials with stimulus on the left (red) and right (blue) for 4 example days of an example balanced mouse with bilateral DLS/DMS recordings. Average psychometric curves on each day shown as insets. Vertical dashed lines indicate stimulus onset, stimulus center, and reward delivery time. Error bars indicate ± SEM across trials.

(D) Average time warped DMS DA signals in correct trials with stimulus ipsilateral (cyan) and contralateral (fuchsia) to the recording fiber for 4 example days of an example one-sided mouse with bilateral DMS recordings.

(legend continued on next page)

(E) Average time warped DLS and DMS DA signals on days with small (<0.05) and large (>0.2) psychometric slope in correct trials with stimulus ipsilateral (cyan) and contralateral (fuchsia) to the recording fiber, as well as zero-contrast trials (gray).

(F) Cf. Figure 2I, bar plots showing average DLS and DMS DA stimulus responses across mice for correct trials in days 1–3, days >3 with accuracy n.s. greater than 0.5 and expert days (accuracy n.s. smaller than 0.7). In all panels, error bars indicate ± SEM across mice, unless specified differently. *p* values calculated using two-sided paired t test.

(G) First day of training time warped DLS and DMS DA signals in correct trials with contralateral/ipsilateral stimulus, as well as zero-contrast trials. Average first-day psychometric curves shown as insets. No stimulus responses were significantly different from zero-contrast trial responses ($p > 0.05$; one-way ANOVA).

(H) Schematic, behavioral and DLS (top) and DMS (bottom) DA results of the reward size manipulation experiment (STAR Methods). Left, schematic showing the alternating day structure of the experiment. Middle left, average psychometric curves on days with high reward in correct right stimulus (blue) and left stimulus (red) trials. Middle right, average time warped DLS and DMS DA signals in correct trials with associated stimulus (only contralateral for DMS) separated by reward size. Right, bar plots quantifying stimulus and outcome DA responses. *p* values calculated using two-sided paired t test. Error bars indicate ± SEM across mice. DMS stimulus responses for low and high reward are n.s. different both when two-sided t test is performed over mice and over days.

(I) Regression of early difference in DLS DA responses to R-L stimuli (average across days 4–8) against late difference in DLS DA responses (average across final 5 days of training). Each point represents a mouse. *p* value calculated from the exact distribution of r. Shaded region indicates 95% confidence interval.

(J) Average difference in DLS DA responses to right and left stimuli (R-L) and difference in DLS DA rewarded outcome responses after R-L stimuli in early days (4–8) and late days (final 5) for each mouse, cf. Figure S1N for behavioral data. Colors come from average psychometric slope asymmetry (see Figure S1L).

(K) Difference in DLS DA responses to rewarded outcomes after right and left stimulus (R-L) over days per mouse (thin) and for the 3 clusters from Figure 1J (thick). Number of days limited to 25, cf. Figure 3A.

(L) Difference in DLS DA responses to rewarded outcomes after R-L stimulus vs. bias, cf. Figure 3D.

(M) Difference in DLS DA responses to rewarded outcomes after R-L stimulus vs. accuracy, cf. Figure 3E.

(N) Average DLS DA stimulus-aligned signals (top), time warped signals (middle) and bar plots with average DLS DA stimulus responses (bottom) across all mice for increasing RT values and a fixed psychometric slope range (>0.325), split by correct and incorrect trials. *p* values calculated using two-sided paired t test. Error bars indicate ± SEM across mice. n.s., $p > 0.05$, $^{*}p < 0.05$, $^{**}p < 0.005$, and $^{***}p < 0.0005$ in all panels.

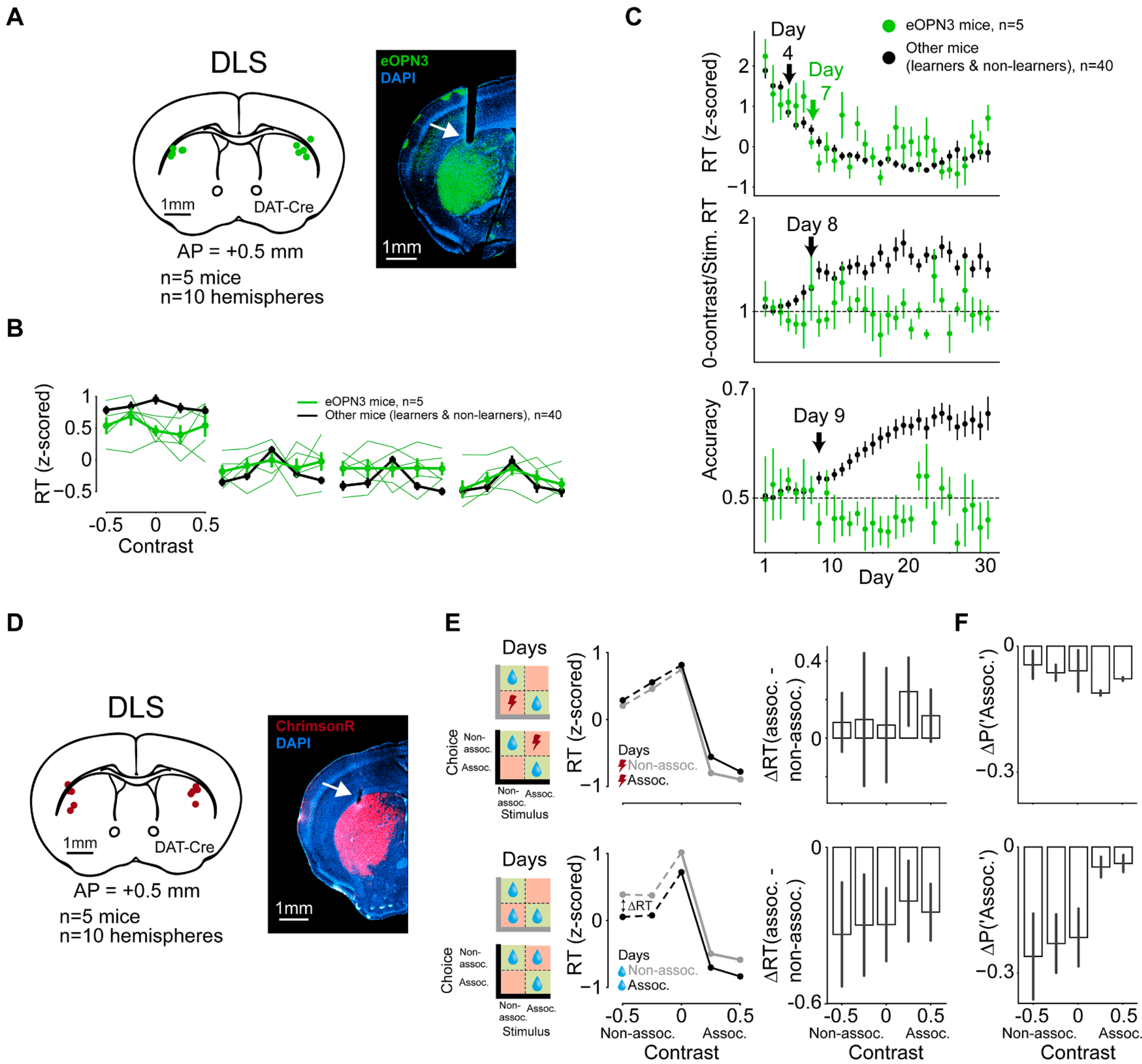

Figure S7. Further quantification of DLS DA optogenetic results, related to Figure 4

(A) Left, the locations of DLS optic fiber implants of eOPN3 mice estimated from post-mortem histological examinations overlaid on a brain slice schematic. Right, example histology slice showing fiber tract (white arrow) and eOPN3 opsin expression (STAR Methods).

(B) Chronometric curve over quartiles for individual eOPN3 mice (green, thin), averaged across eOPN3 mice (green, thick) and averaged across all the other mice that were trained on the task for at least 4,300 trials (black). In all panels, error bars indicate ± SEM across mice. For each mouse, the *Z* score was computed by first finding the mean RT per contrast across trials in each day, these values were subsequently standardized across days and contrast values, and finally averaged across days in each quartile.

(C) Measures of general RTs, the ratio of RTs on zero-contrast trials and trials with stimuli and accuracy over the first 30 days of training. First plot, *Z* scored RTs (across all trial types) over training days averaged across eOPN3 (green) and all other trained mice (black). Arrows indicate the first data points that were significantly different from day 1 averages ($p < 0.05$, estimated via two-sided t test). *Z* scoring was performed by standardizing each mouse's mean RT per day using data from all its days. Second plot, the ratio of the average (non *Z* scored) RTs measured in zero-contrast trials to the average RTs in left and right stimulus trials. Third plot, accuracy over training days. For all measures, only days with more than 50 trials were considered in the analysis.

(D) Left, the locations of DLS optic fiber implants of ChrimsonR mice estimated from post-mortem histological examinations overlaid on a brain slice schematic. Right, example histology slice showing fiber tract (white arrow) and ChrimsonR opsin expression (STAR Methods).

(E) Cf. Figure 4G, average chronometric curves (left) and a bar plot of their differences (right) for the mice and manipulations used in the ChrimsonR optogenetic (and water delivery) experiment. Z scored RTs defined as in (B). Only trials with RT < 12 s were analyzed for the stimulation experiment, and RT < 5 s were analyzed for the water delivery experiment.

(F) Cf., Figure 4G, average difference in psychometric curves with stimulation/water delivery in incorrect trials with the associated and non-associated stimuli (i.e., assoc. − non-assoc.) for mice in which both the ChrimsonR DLS stimulation and reward delivery experiments were performed ($n = 3$).

CellPress
OPEN ACCESS

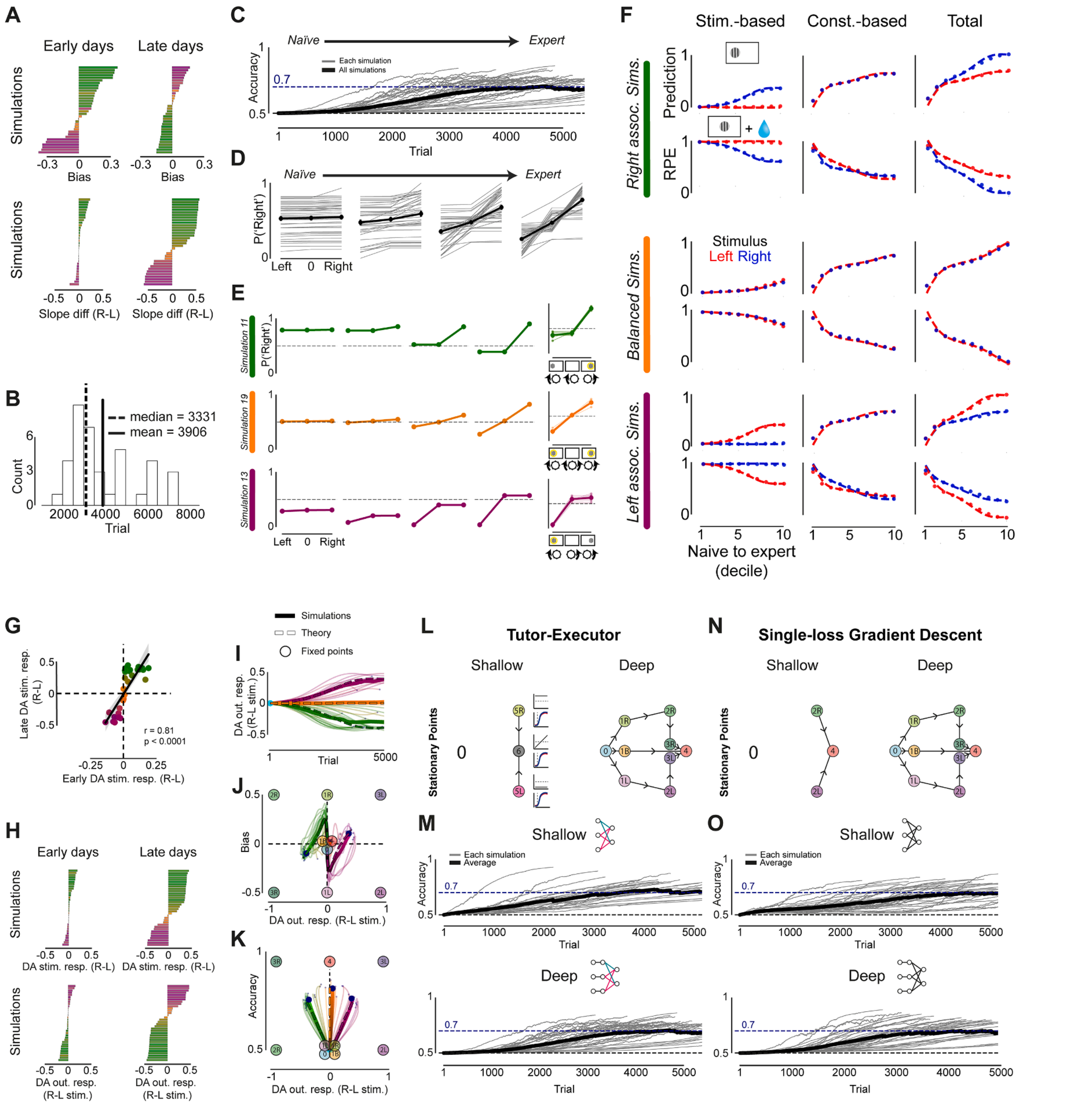

Figure S8. The deep tutor-executor network accounts for mouse behavior and DA signals throughout learning, related to Figures 5 and 6

(A) Cf. Figure S1N, the model's average bias and slope difference early in training (average across trials 1,000–2,000) and late in training (average across final 1,000 trials) for each simulation. Colors come from average psychometric slope asymmetry of each simulation (see Figure S1L).

(B) Cf. Figure S1C, histogram of the number of trials that simulations required to reach 70% accuracy.

(C) Cf. Figure 1B, accuracy over trials per simulation (gray) and averaged across all simulations (black). Dashed lines indicate chance level (black) and 70% accuracy level (blue). Number of trials limited to the average simulation length.

(D) Cf. Figure 1C, psychometric curves over quartiles per simulation (gray) and averaged across all simulations (black). In all panels, error bars indicate ± SEM across simulations unless specified differently.

(E) Cf. Figure 1D, left, psychometric curves from 3 example simulations over quartiles throughout learning. Right, per simulation (thin) and average expert psychometric curves (acc. > 70%) clustered by trajectory type (thick). Cluster labels for each simulation obtained from Figure 5G, colors obtained from average psychometric slope asymmetry.

(F) Cf. Figure 2D, in order from left to right columns: average stimulus-based (i.e., model-derived DLS DA signals), constant-based and total predictions and RPEs over deciles in correct trials with stimulus on the left (red) and right (blue) for the three clusters from Figure 5G. Points show simulation averages and thick dashed lines show average dynamics.

(G) Cf. Figure S6I, regression of early difference in model-derived DLS DA responses to R-L stimuli against late difference in model-derived DLS DA stimulus responses. Each point represents a simulation. *p* value is calculated from the exact distribution of r. Shaded region indicates 95% confidence interval.

(legend continued on next page)

(H) Cf. Figure S6J, the average difference in model-derived DLS DA responses to right and left stimuli (R-L) and difference in model-derived DLS DA rewarded outcome responses after R-L stimuli early in training (average across trials 1,000–2,000) and late in training (average across final 1,000 trials) for each simulation. Colors come from average psychometric slope asymmetry.

(I) Cf. Figure S6K, difference in model-derived DLS DA responses to rewarded outcomes after R-L stimuli over trials per simulation (thin) and for the 3 clusters from Figure 5G (thick). Number of trials limited to 5,000.

(J) Cf. Figure S6L, difference in model-derived DLS DA responses to rewarded outcomes after R-L stimulus vs. bias. Stationary points here and in (K) are plotted using the average behavior and model-derived DLS DA arising from their corresponding weight configurations.

(K) Cf. Figure S6M, difference in model-derived DLS DA responses to rewarded outcomes after R-L stimulus vs. accuracy.

(L) Comparison between the stationary points of a shallow (left column) and deep (right column) version of the tutor-executor network. Note that 0 is not a stationary point of the shallow network and is placed in the left panel for reference. The shallow network has three stationary points which have different network configurations to those of the deep network. They are hence labeled with the increasing numbers 5L, 5R, and 6, accompanied by schematics of their associated behavior and neural predictions. These correspond to states where the shallow network is only making left choices (5L), right choices (5R), and the global optimum (6).

(M) Comparison of the accuracy over trials for simulations from a shallow (top) and deep (bottom) version of the tutor-executor network. Thin gray lines show the accuracy curves for each simulation, and the thick black line indicates the average across simulations.

(N) Comparison between the stationary points of a shallow (left column) and deep (right column) version of the single-loss gradient descent network.

(O) Comparison of the accuracy over trials for simulations from a shallow (top) and deep (bottom) version of the single-loss gradient descent network. The learning curve of both deep models better captures mice data (cf. Figure 1B) than the shallow models.

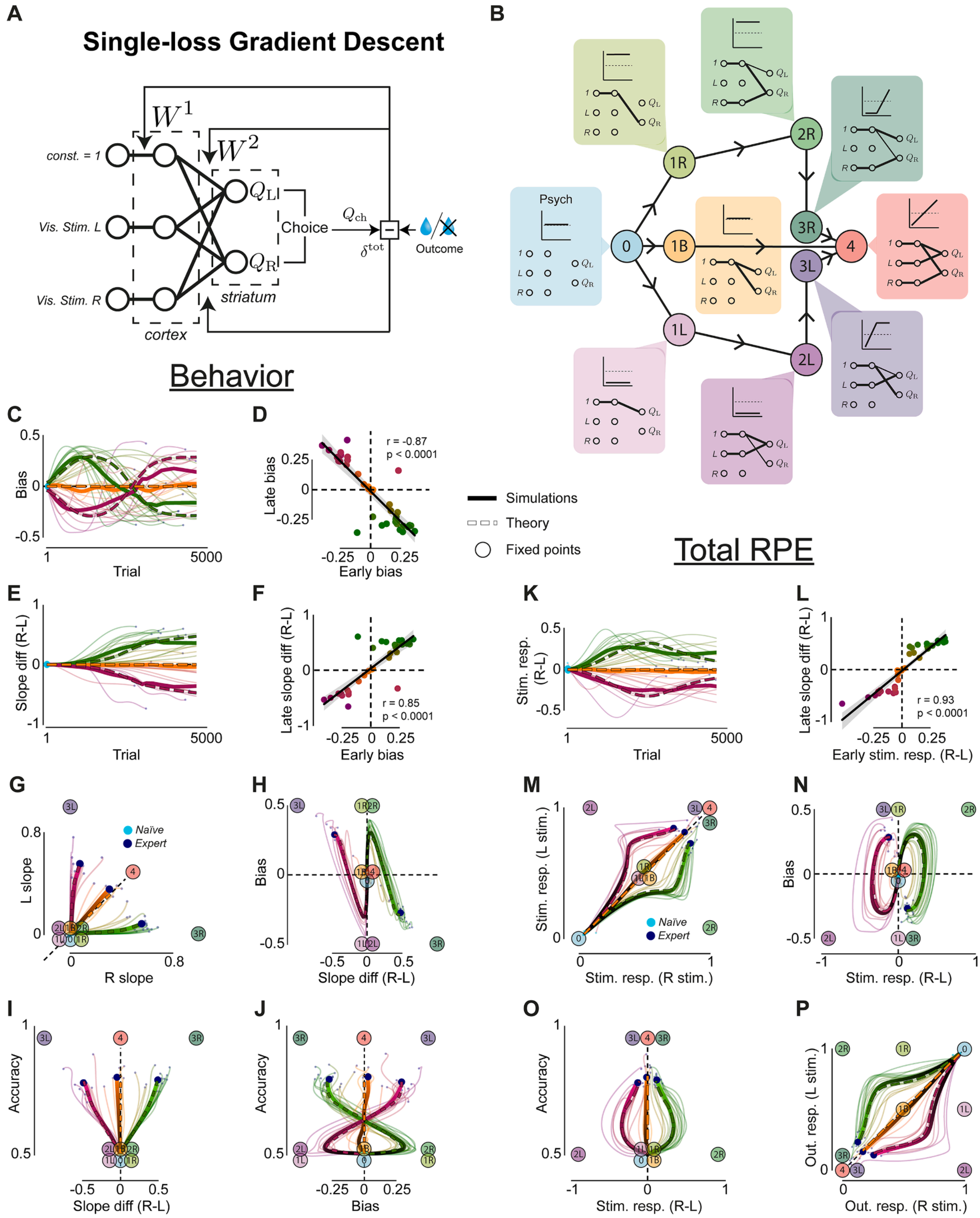

Figure S9. Deep RL network trained with single-loss gradient descent captures behavioral trajectories with a teaching signal different from DLS DA, related to Figure 7

(A) Schematic of the deep linear "single-loss" deep RL network architecture and learning rule (STAR Methods).

(B) Schematic of the stationary point structure with behavioral predictions as well as corresponding network weight configurations. The connecting lines with arrows represent the steepest heteroclinic orbits into/out of each stationary point (STAR Methods). All the stationary points are saddle points except for 4, which is the global minimum.

(C) Cf. Figure 1F, bias over trials per simulation (thin), for the 3 clusters from (G) (thick), and for the average dynamics (thick dashed). Here, and in (E) and (K), the number of trials is limited to 4,667. Thick dashed lines in all panels indicate analytical trajectories derived from the average dynamics (STAR Methods).

(D) Cf. Figure 1G, regression of early bias (average across trials 1,000–2,000) against late bias (average across final 1,000 trials). Each point represents a simulation. p value calculated from the exact distribution of r. In all panels, shaded regions indicate 95% confidence interval across mice.

(legend continued on next page)

(E) Cf. Figure 1H, difference between right and left psychometric slopes over trials per simulation (thin) and for the 3 clusters from (G) (thick).

(F) Cf. Figure 1I, regression of early bias against late slope difference. Each point represents a simulation. p value calculated from the exact distribution of r.

(G) Cf. Figure 1J, right vs. left slope over trials per simulation (thin) and for 3 clusters (thick). Clusters and colors obtained using the same procedure as for the behavioral data in Figure 1J. The clusters from this analysis are used in all other panels. Stationary points here and in (H)–(J) are plotted using the average behavior arising from their weight configurations.

(H–J) Cf. Figures 1K–1M, in order, difference in right and left (R-L) slope vs. bias, R-L slope vs. accuracy and bias vs. accuracy over trials per simulation (thin) and for the 3 clusters from (G) (thick).

(K) Cf. Figure 3A, difference in total RPE signals after right and left stimuli (R-L) over trials per simulation (thin) and for the 3 clusters from (G) (thick).

(L) Cf. Figure 3B, regression of early stimulus-evoked total RPE (average across trials 1,000–2,000) against late slope difference (average across final 1,000 trials). Each point represents a simulation. p value calculated from the exact distribution of r.

(M–P) Cf. Figures 3C–3F, in order, right vs. left stim.-evoked total RPEs; difference in total RPE evoked by right and left stimuli (R-L) vs. bias; difference in total RPE evoked by right and left stimuli (R-L) vs. accuracy; and outcome-evoked total RPE signals after right stimulus vs. after left stimulus per simulation (thin) and for the 3 clusters from (G). Stationary points are plotted using the average total RPEs arising from their weight configurations.

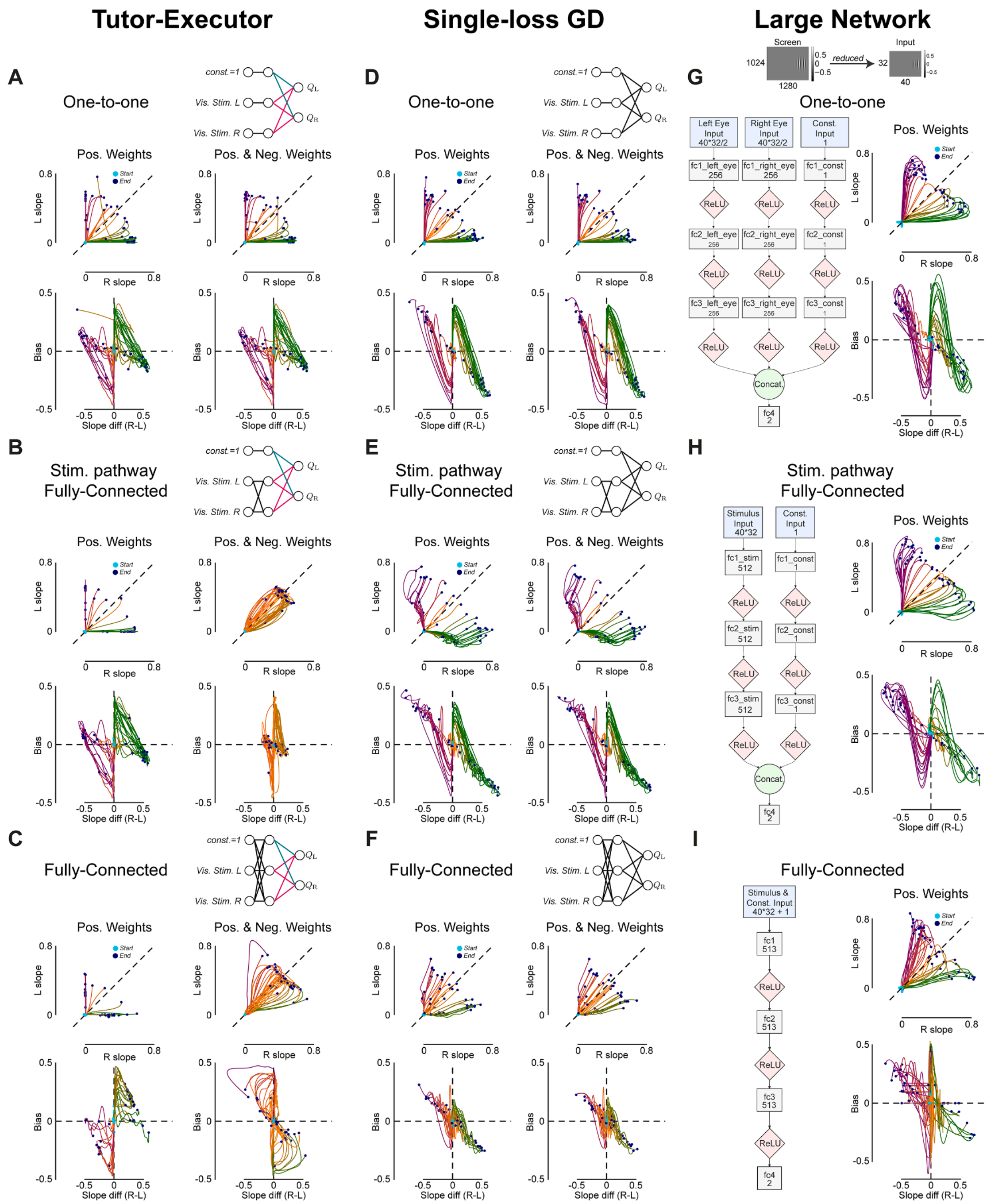

Figure S10. Exploring model constraints and generalization to larger networks, related to Figure 5

(A) Top, schematic of a tutor-executor network with one-to-one connections in W^1. Bottom, right vs. left slope (cf. Figure 1J) and difference in right and left slope (R-L) vs. bias (cf. Figure 1K) over trials per simulation for simulations with (left) and without (right) the nonnegativity constraint applied to the network weights. Trajectory colors obtained from the average slope asymmetry as in Figure S1L. All simulations (n = 40) are depicted regardless of whether they reach expertise (i.e., acc. > 70%). Simulations were initialized as in Figure 5 (see STAR Methods).

(B) Same as (A) for a tutor-executor network with cross-connections between the stimulus inputs in W^1.

(C) Same as (A) and (B) for a fully connected tutor-executor network.

(D–F) Same analysis as in (A)–(C) applied to a network trained with the single-loss gradient descent learning rule. Simulations were initialized as in Figure S7 (see STAR Methods).

(G) Left, mermaid diagram of a scaled-up non-linear version of the one-to-one network with segregated fully connected channels for each input which merge only in the final layer. Right, right vs. left slope and difference in right and left slope (R-L) vs. bias over trials per simulation trained with the single-loss gradient descent

(legend continued on next page)

rule and the nonnegativity constraint applied to the network weights. Trajectory colors obtained from the average slope asymmetry as in Figure S1L. Only simulations that reached 70% accuracy in less than 8,500 trials are depicted. Simulations were initialized such that the effective weights in each channel were as in Figure S7 (see STAR Methods).

(H) Same as (G) for a scaled-up non-linear version of the network with cross-connections only between the stimulus inputs.

(I) Same as (G) and (H) for a scaled-up version of the fully connected network.

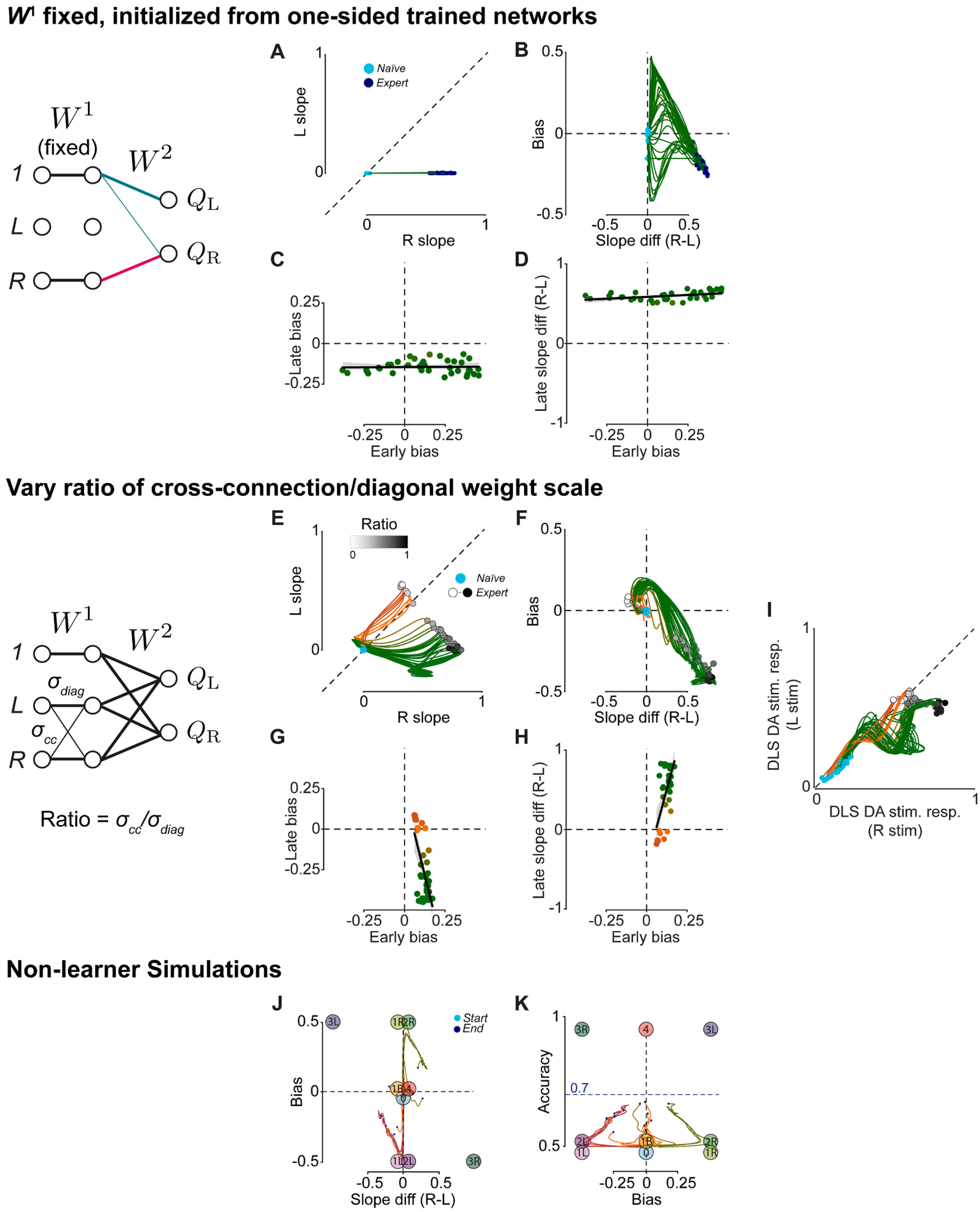

Figure S11. Effect of non-uniform input representations, stimulus pathway cross-connections, and the trajectories of simulations that did not reach expertise, related to Figures 5 and 7

(A–D) Effect of fixing W^1 to values from the average network configuration of right-associating simulations from Figure 5. Only simulations that reached expertise (i.e., acc. > 70%) from n = 40 simulations are shown. Simulations are from tutor-executor networks where the W^1 update is set to 0. Trajectory colors obtained from the average slope asymmetry as in Figure S1L.

(A) Cf. Figure 1J, right vs. left slope over trials per simulation.

(B) Cf. Figure 1K, difference in right and left slope (R-L) vs. bias.

(C) Cf. Figure 1G, regression of early bias (average across trials 1,000–2,000) against late bias (average across final 1,000 trials). Each point represents a simulation. In all panels, shaded regions indicate 95% confidence interval across simulations.

(D) Cf. Figure 1I, regression of early bias against late slope difference. Each point represents a simulation.

(legend continued on next page)

(E–H) Effect of varying the relative magnitude of W^1 stimulus pathway cross-connection to diagonal weights at initialization. Same figures as in (A)–(D) for simulations with fixed seeds for the generated stimulus/reward sequence, softmax choice rule and initialization weights, but with different variances for the Gaussians from which the cross-connection weights are sampled.

(I) Cf. Figure 3C, right vs. left model-derived DLS DA stimulus responses over trials per simulation. Only simulations that reached expertise (i.e., acc. > 70%) are shown. Simulations are from large single-loss gradient descent networks.

(J and K) Cf. Figure S1P, slope difference vs. bias and bias vs. accuracy over trials of tutor-executor simulations that did not reach accuracy $\geq$70% in less than 8,500 trials from a total of n = 120. Simulations were initialized as in Figure 5. Numbered circles represent stationary points of the learning dynamics (see Figure 7) plotted using the average behavior arising from their corresponding weight configurations.

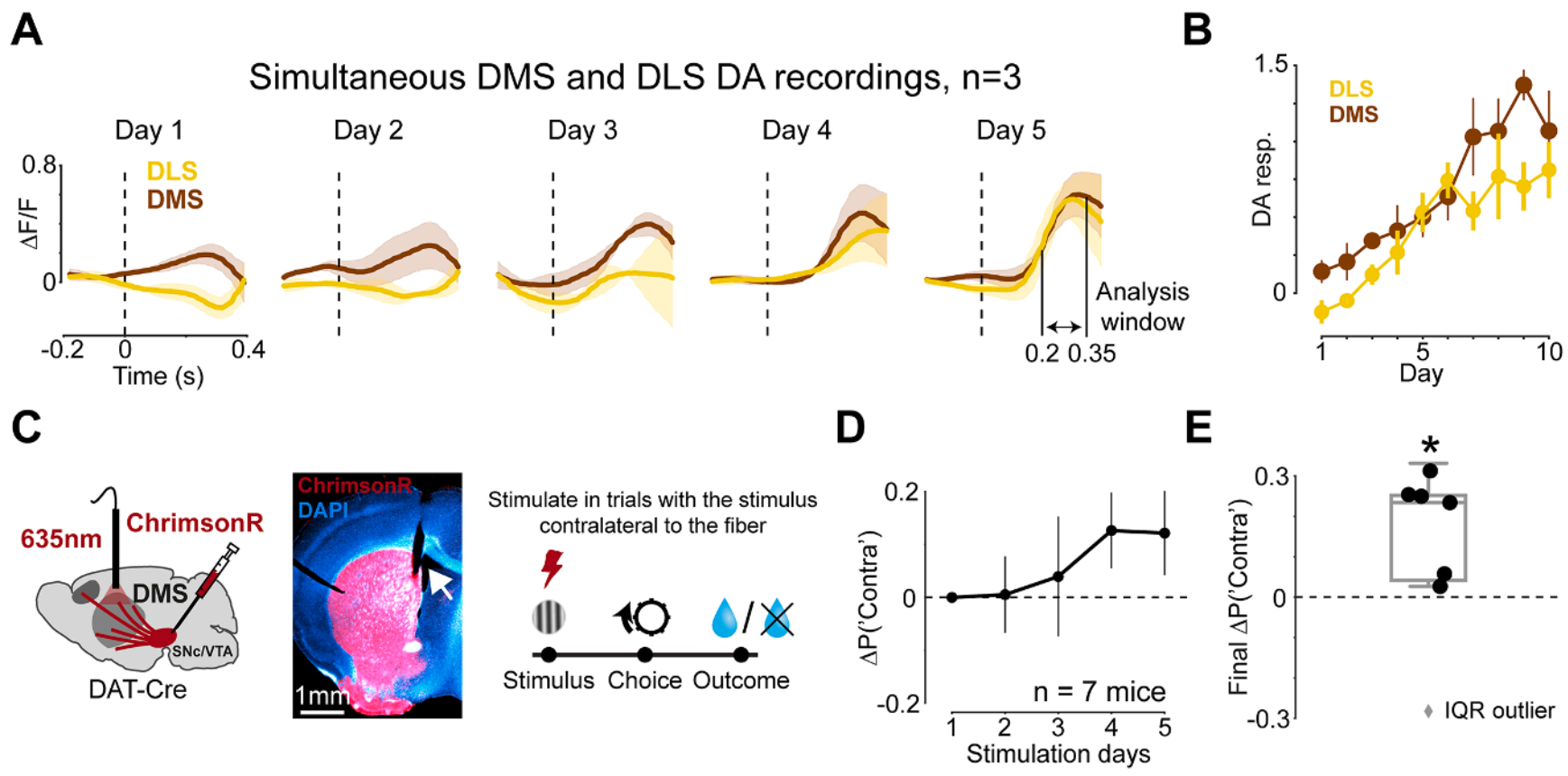

Figure S12. Comparison of DMS and DLS DA signals and effect of DMS DA optogenetic stimulation, related to Figures 2 and 4

(A) Average stimulus-aligned DA signals for mice with simultaneous DLS and DMS recordings that associated the stimulus contralateral to the DMS fiber. Average is across correct trials with the stimulus contralateral (ipsilateral) to the DMS (DLS) fiber.

(B) Average stimulus response over first 10 days of learning for the same trials and mice as in (A) (see analysis window).

(C) Schematic explaining the optogenetic experiment using the excitatory opsin ChrimsonR in DMS, alongside example histology slice showing fiber tract (white arrow) and opsin expression (STAR Methods).

(D) Change from day 1 in the probability of making the choice contralateral to the stimulation fiber, i.e., P(“Contra”), over 5 stimulation days.

(E) Boxplot of day 5 ΔP(“Contra”). Each point is a mouse. *p* value calculated using two-sided one-sample t test against 0, excluding interquartile range (IQR) outlier.

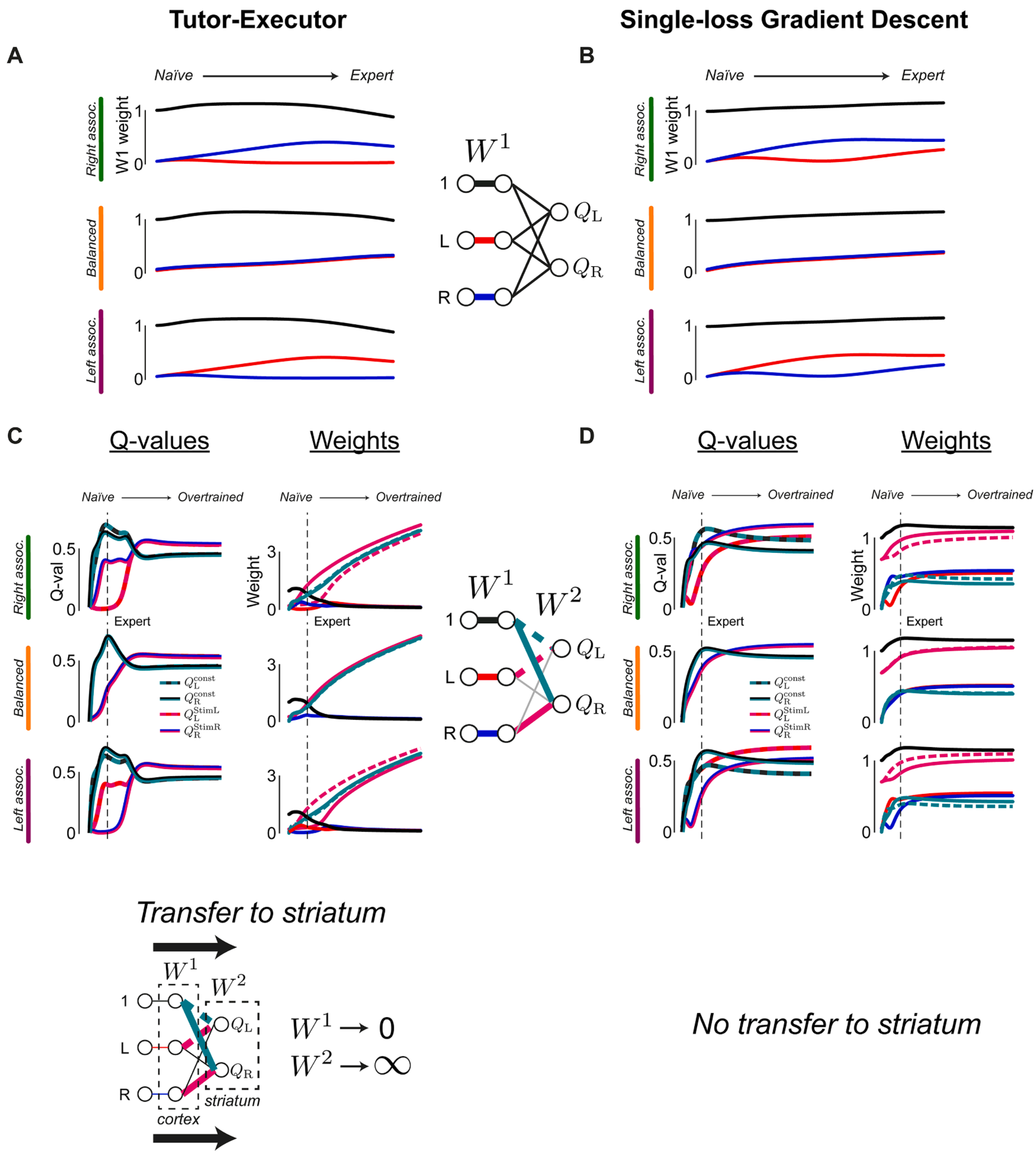

Figure S13. Tutor-executor learning rule causes transfer to striatum, related to Figure 7

(A) W^1 weights from naive to expert in examples of right- (top), balanced (middle), and left-associating (bottom) average dynamics of the tutor-executor network (same as dashed trajectories in Figure 5). Right- and left-associating trajectories were obtained by initializing the average dynamics with a small degree of left and right bias, whereas the balanced trajectory comes from a network initialized without bias (STAR Methods).

(B) W^1 weights from naive to expert from the average dynamics of the single-loss gradient descent network (same as dashed trajectories in Figure S9).

(C) Left, stimulus- and constant-pathway Q-values (derived from the product of elements in W^1 and W^2, see STAR Methods) of the average dynamics of the tutor-executor network from naive to overtrained. Overtrained: trained 8 times longer than the training used for naive to expert. We do not plot the Q-values of the "incorrect" associations (i.e., left stimulus with right choice and right stimulus with left choice) as these remain around 0. Right, W^1 and W^2 weights from naive to overtrained. Again, we do not plot the weights that connect the inputs with the wrong choices as these remain around 0.

(D) Similar to (C) but for the single-loss gradient descent network.
